# Single-cell RNA sequencing reveals developmental heterogeneity of blastomeres during major genome activation in bovine embryos

**DOI:** 10.1038/s41598-018-22248-2

**Published:** 2018-03-06

**Authors:** Ilaria Lavagi, Stefan Krebs, Kilian Simmet, Andrea Beck, Valeri Zakhartchenko, Eckhard Wolf, Helmut Blum

**Affiliations:** 1 Laboratory for Functional Genome Analysis (LAFUGA), Gene Center, LMU Munich, Munich, Germany; 2 Graduate School of Quantitative Biosciences Munich (QBM), Gene Center, LMU Munich, Munich, Germany; 3 Chair of Molecular Animal Breeding and Biotechnology, Gene Center and Department of Veterinary Sciences, LMU Munich, Munich, Germany

## Abstract

Embryonic development is initially controlled by maternal RNAs and proteins stored in the oocyte, until gene products gradually generated by the embryo itself take over. Major embryonic genome activation (EGA) in bovine embryos occurs at the eight- to 16-cell stage. Morphological observations, such as size of blastomeres and distribution of microvilli, suggested heterogeneity among individual cells already at this developmental stage. To address cell heterogeneity on the transcriptome level, we performed single-cell RNA sequencing of 161 blastomeres from 14 *in vitro* produced bovine embryos at Day 2 (n = 6) and Day 3 (n = 8) post fertilization. Complementary DNA libraries were prepared using the Single-Cell RNA-Barcoding and Sequencing protocol and sequenced. Non-supervised clustering of single-cell transcriptome profiles identified six clusters with specific sets of genes. Most embryos were comprised of cells from at least two different clusters. Sorting cells according to their transcriptome profiles resulted in a non-branched pseudo-time line, arguing against major lineage inclination events at this developmental stage. In summary, our study revealed heterogeneity of transcriptome profiles among single cells in bovine Day 2 and Day 3 embryos, suggesting asynchronous blastomere development during the phase of major EGA.

## Introduction

During early stages of embryonic development, maternal RNAs and proteins are gradually degraded, while embryonic transcripts are synthesized. This process is called maternal-to-embryonic transition (MET) and involves embryonic genome activation (EGA) (reviewed in)^1^. EGA occurs in distinct waves, which are species-specific. Major EGA occurs at the two-cell stage in mouse embryos, at the four- to eight-cell stage in human and pig embryos, and at the eight- to 16-cell stage in bovine embryos (reviewed in)^2^. Recently, time-lapse microscopy was used to study lineage specification in early bovine embryos by tracing the allocation of blastomeres^3^. In the majority of embryos, cells intermingled between the third and fourth cell cycle, yielding a random allocation pattern. Single-cell RNA sequencing (scRNA-seq) is increasingly used to investigate mechanisms regulating the formation of the three cell lineages (trophectoderm, epiblast and primitive endoderm) during embryo development. The transcriptomes of these cell lineages have already been investigated in mouse^4,5^ and human embryos^6,7^, and in differentiating human embryonic stem cells^8^. In bovine, the transcriptome of whole embryos has been studied at different developmental stages^9,10^. More recently, transcript profiling of single embryonic cells for a set of candidate genes has been performed for different stages from zygote to blastocyst^11,12^, providing new insight into lineage specification events in bovine embryos. However, holistic single-cell transcriptome analysis has not been performed in bovine embryos during major EGA (eight-cell to 16-cell stage) yet. Our study applied scRNA-seq on these developmental stages to provide a refined view into the timing of major EGA, developmental heterogeneity, and potential early lineage inclination events in bovine embryos.

## Results

### Selection of developmentally competent *in vitro* produced embryos

The kinetics of early embryo development *in vitro* is strongly associated with the potential to form a blastocyst and to establish pregnancy^13^. Therefore, we studied a total of 541 bovine embryos for 168 hours after fertilization by time-lapse microscopy. The timing and duration of the first, second and third cleavages and their effects on blastocyst rate were analysed in order to select embryos with high developmental potential. The highest blastocyst rate (75%) was detected, when the first embryonic cleavage occurred between 25.6 and 27.1 hours post fertilization (hpf). The optimal time ranges for the second and third cleavages were 33.4 to 36.2 hpf and 41.6 to 43.7 hpf, respectively. The optimal duration of the two-cell stage was 7.7 to 8.6 hours, resulting in blastocyst rates of 77 to 81% (Supplementary Fig. S1)^14^. For the present study, six Day 2 and eight Day 3 embryos were selected to fit most closely into the optimal developmental kinetics (Table 1). Single cells were prepared and processed for sequencing. In total, six to 9 cells per Day 2 embryo and 13 to 17 cells per Day 3 embryo were analysed.

**Table 1:** Cleavage timing, embryo collection time and number of cells in Day 2 and Day 3 embryos used for single-cell transcriptome profiling.

Embryo designation	1^st^ cleavage (hpf)	2^nd^ cleavage (hpf)	3^rd^ cleavage (hpf)	Embryo collection (hpf)	Total cell number
Day2-E1	25:45	33:51	41:15	44:00	8*
Day2-E2	27:01	34:06	42:50	44:00	9
Day2-E3	27:01	34:26	39:45	44:00	9
Day2-E4	27:11	35:11	42:10	45:00	8
Day2-E5	27:35	34:25	45:25	45:30	8
Day2-E6	27:45	37:30	45:04	45:04	6
Day3-E1	29:18	38:45	47:07	67:00	15
Day3-E2	26:08	34:23	41:17	67:00	16
Day3-E3	27:48	35:53	44:02	67:00	16
Day3-E4	25:53	33:45	40:47	67:00	13
Day3-E5	29:56	37:16	45:20	69:00	17
Day3-E6	29:51	37:31	45:15	69:00	14
Day3-E7	27:15	36:20	44:15	71:00	16
Day3-E8	25:35	33:45	39:56	71:00	16

*In vitro* developing embryos were observed by time-lapse microscopy, and embryos with high developmental potential were selected based on the timing (hours post fertilization; hpf; shown as hours:minutes) of the first three cleavage divisions. *1 cell was lost during the cell collection.

### Filtering and Quality Control of RNA-Seq Data

Transcriptome profiles of 170 single cells were generated by Single-Cell RNA Barcoding and Sequencing (SCRB-Seq)^15^. On average, 1,896,797 reads per library were obtained. Subsequently, the unique molecular identifiers (UMI) were counted as a measure for the complexity of the sequencing libraries and used for further analyses to exclude PCR duplicates. On average, 45,000 UMI per library were obtained. The numbers of generated reads, UMI and detected genes per library are reported in Supplementary Table S1. Sequencing data of nine cells were excluded from further analyses because their UMI count was below the empirical threshold of 2,000 (Supplementary Fig. S2). In total, 10,772 genes were captured by combining the transcriptome profiles of 161 cells. Saturation plots are shown in Supplementary Figs S3–S5.

### Cluster Analysis of Single-Cell Transcriptome Profiles

**Figure 1:**
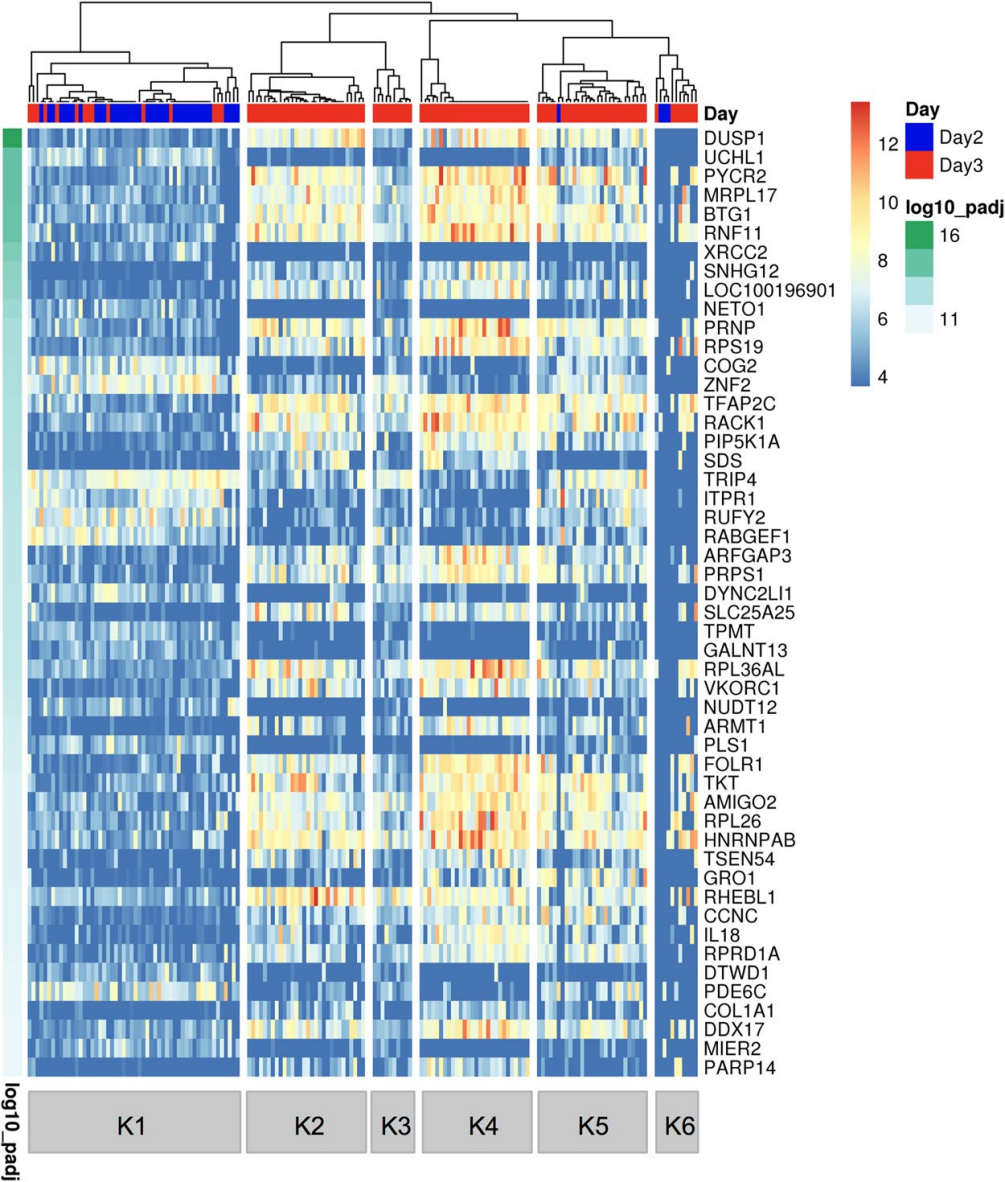
Single-cell Consensus Clustering (SC3). Single-cell transcriptome profiles of 161 blastomeres from six Day 2 embryos and eight Day 3 embryos were analysed with the SC3 tool^16^. Differentially abundant transcripts (DAT) were identified with the non-parametric Kruskal-Wallis test. DAT were clustered with a pre-set number of six clusters and the results for the 50 top genes are shown. The adjusted p-value is shown on the left. A blue or red colour label in the embryo ID row indicates blastomeres collected from Day 2 or Day 3 embryos, respectively.

In order to search for cell populations present in sampled embryos, cluster analyses were performed with two different unsupervised tools for single-cell sequencing data sets. For the SC3 R package tool^16^, the number of clusters for calculation of the consensus matrix was set to six. This value had been obtained using the Tracy-Widom theory on random matrices to estimate the optimal number of clusters k^17^. The SC3 pipeline was used to cluster the single cells, and 2,494 differentially abundant transcripts (DAT; p < 0.01) were identified (Supplementary Table S2). Figure 1 shows the assignment of the 161 cells to the six clusters and plots the colour-coded abundance levels of the 50 most significant DAT sorted according to their p-value. Most embryos were comprised of cells from at least two different clusters. Three clusters (K1, K5 and K6) contained cells of both Day 2 and Day 3 embryos, the other three clusters (K2, K3 and K4) exclusively cells from Day 3 embryos (Table 2).

**Table 2:** Distribution of the cells collected from Day 2 and Day 3 bovine embryos through the clusters identified by the SC3 tool.

Embryo designation	K1	K2	K3	K4	K5	K6	No. cells not analyzed	No. embryo cells
Day2-E1	4	—	—	—	—	2	2	8
Day2-E2	9	—	—	—	—	—	—	9
Day2-E3	7	—	—	—	—	—	2	9
Day2-E4	6	—	—	—	—	1	1	8
Day2-E5	7	—	—	—	1	—	—	8
Day2-E6	6	—	—	—	—	—	—	6
Day3-E1	13	—	—	—	—	1	1	15
Day3-E2	14	—	—	—	—	—	2	16
Day3-E3	2	—	—	—	13	1	—	16
Day3-E4	—	—	—	11	—	2	—	13
Day3-E5	—	6	9	—	—	2	—	17
Day3-E6	—	13	1	—	—	—	—	14
Day3-E7	—	11	—	3	—	1	1	16
Day3-E8	—	—	—	14	—	1	1	16

The original number of cells in each embryo is reported in the last column. Some cells were lost at the time of collection or filtered out because of low quality of their transcriptome; the number of these cells is reported in the penultimate column.

**Figure 2:**
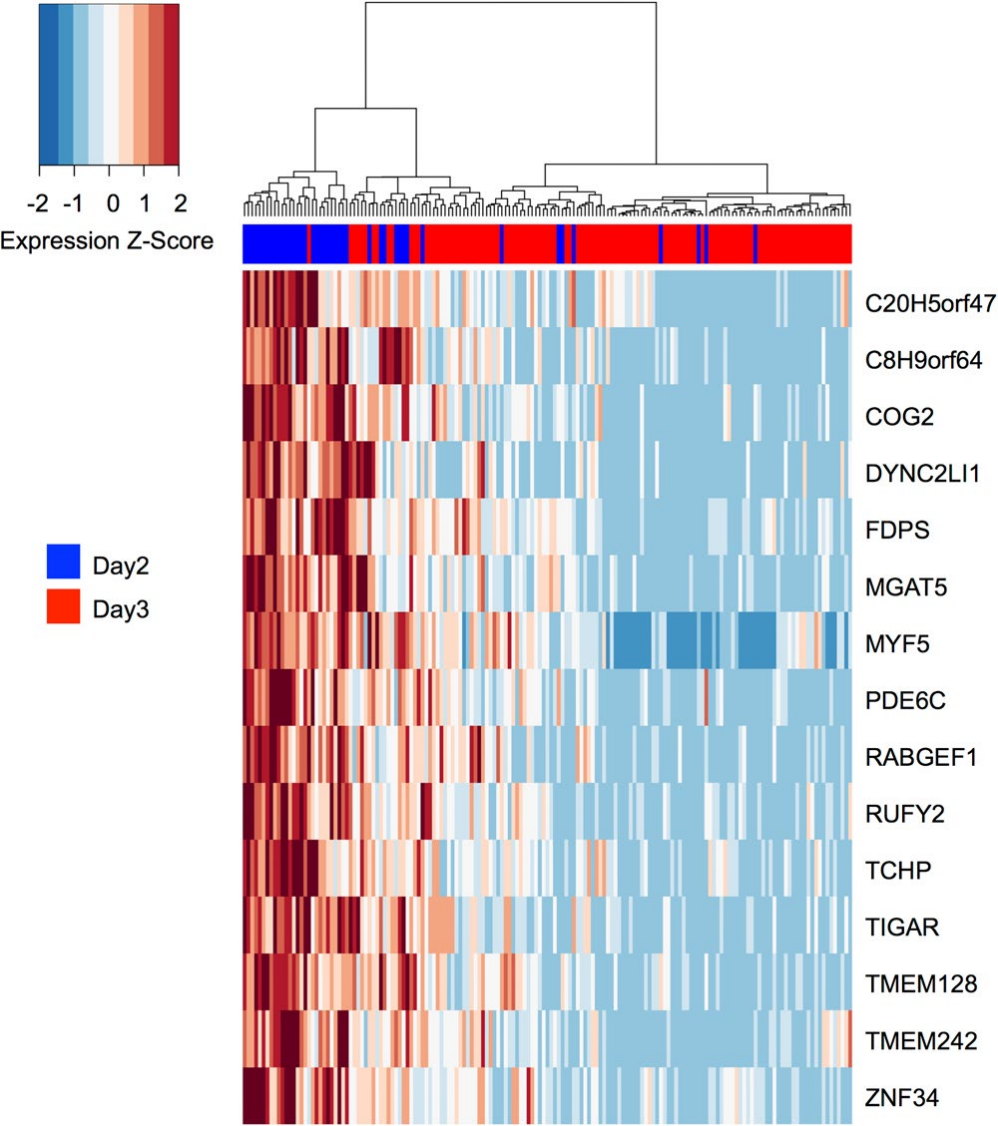
Michaelis-Menten Modelling of Dropouts (M3Drop). The single-cell transcriptome dataset of 161 blastomeres from six Day 2 and eight Day 3 embryos was analysed with the M3Drop tool^18^. Genes with detectable levels of transcripts in all blastomeres (genes not affected by dropouts; n = 15) were identified with the Z-test and hierarchical clustering was performed only on these genes. Expression values are displayed as Z-scores of log2 transformed expression data (adding a pseudo-count of 1). A blue colour label in the embryo ID row marks cells collected from Day 2 embryos; cells from Day 3 embryos are marked in red. Note that cells cluster independently of embryo age.

In order to study the influence of dropouts (zero read counts for certain genes, due to failure of reverse transcription or low read counts) on the clustering and number of DAT, the dataset was analysed with the M3Drop R package tool^18^. This tool identified only 15 genes not affected by dropout (Fig. 2), which were a subset of the 2,494 DAT identified by the SC3 approach (Supplementary Fig. S6).

### Cluster Specific Marker Genes

Cluster specific marker genes were identified using the SC3 pipeline^16^. Threshold criteria were: adjusted p-value < 0.01; area under the ROC curve (AUROC) >0.85. In cluster K1, 12 marker genes were identified. These genes encode proteins belonging to diverse protein classes, such as serine/cysteine protease, membrane traffic protein, and DNA strand-pairing protein hydrolase. In cluster K2, *RHEBL1*, a gene involved in TORC1 signalling^19^, was found. Cluster K3 showed no statistically significant marker genes. In cluster K4, 88 cluster specific marker genes were identified. One of them, *NANOG*, is involved in the maintenance of pluripotency^20^. Another marker gene of cluster K4 was *FOLR1*, which is expressed in murine embryos from the two-cell stage^21^. Several other K4 marker genes encode 40 S (*RPS19*, *RPS27*, *RPS29*, *RPS4Y1*) or 60 S (*RPL37*, *RPL38*) ribosomal proteins. Another interesting candidate among the K4 marker genes was *KLF5* that is involved in self-renewal of mouse embryonic stem cells^22^. The other marker genes encode proteins belonging to different functional classes, such as kinases, transcription factors, proteins involved in membrane trafficking, and translation initiation factors. Cluster K5 showed the chemokine coding gene *CXCL1* (also known as *GRO1*) as cluster specific gene. Cluster K6 showed no statistically significant marker genes (Supplementary Table S3).

### Gene Set Enrichment Analysis of the Cluster Specific Genes

Gene set enrichment analysis was performed for the cluster specific genes by using the ClueGO^23^ plugin of Cytoscape. This tool was used with the downloadable *Bos taurus* genome, and a p-value < 0.01 was set for filtering the pathways. Statistically significant gene ontology (GO) terms were only found in cluster K4, where “ribosome biogenesis”, “ribosome assembly”, “ribosomal large subunit biogenesis”, “nucleobase biosynthetic process”, “translational elongation” and “cellular amino acid biosynthetic process” were over-represented (Supplementary Table S4).

### Biological Pseudo-Order and Identification of Gene Topics

**Figure 3:**
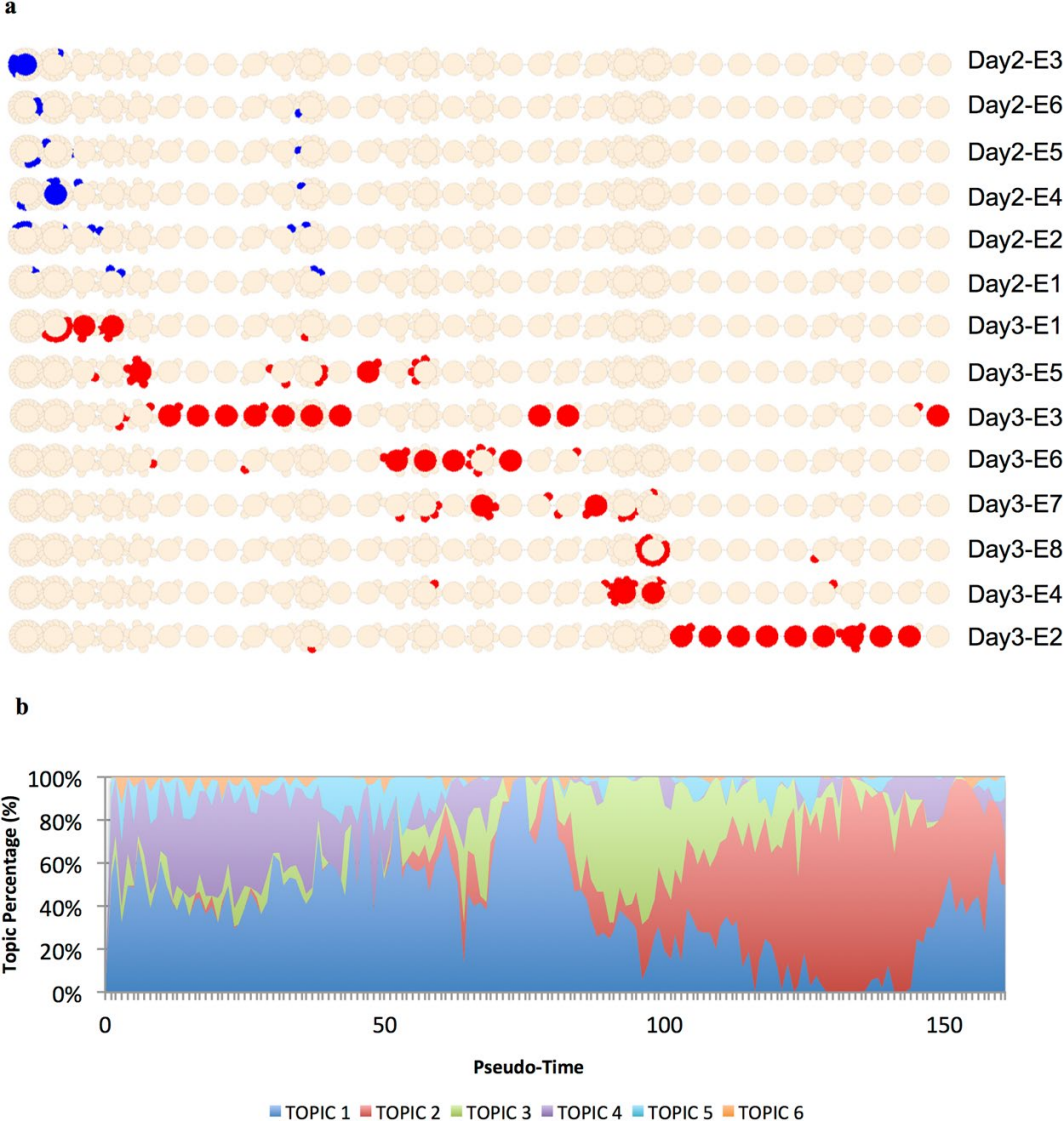
Biological pseudo-time. The single-cell transcriptome dataset of 161 blastomeres from six Day 2 and eight Day 3 embryos was analysed with the CellTree tool^24^. (**a**) Backbone tree. A backbone tree is built by computing a matrix of pairwise distances. This shows the hierarchical relationship between all blastomere transcriptome profiles and aligns the blastomeres in a pseudo-time line. The backbone trees of individual embryos are sorted according to the median position of their cells. Blastomeres from Day 2 embryos are coloured in blue, blastomeres from Day 3 embryos in red. **(b)** Identification of latent groups of genes (topics), which characterise the steps of the development. The number and distribution of topics along the pseudo-time line was obtained by using the Latent Dirichlet Allocation (LDA). To each topic, gene ontology terms were associated. Topic 1 = “translation”, “cell division”; Topic 2 = “translation”, “regulation of translational initiation”, “mRNA splicing, via spliceosome”, “cytoplasmic translation”, “rRNA processing”, “spliceosomal complex assembly”, “negative regulation of mRNA splicing, via splicing”, “cell division”, “regulation of alternative mRNA splicing, via spliceosome”; Topic 3 = “translation”; Topic 4 = “ATP synthesis coupled proton transport”; Topic 5 = “mitochondrial translational elongation”; Topic 6 = “organic hydroxyl compound transport”.

The R package CellTree^24^ was used to order the cells according to their developmental stage. CellTree identifies cells that are good representatives of major steps in development and uses them to construct a backbone of big circles sorted according to developmental progress (biological pseudo-time line). All other cells are aligned as smaller circles branching from the most similar representative cell. Big and small circles constitute a backbone tree. Based on the overall pseudo-time line, embryos were sorted from top to bottom according to the median position of their cells (Fig. 3a).

All single cells from Day 2 embryos were located in the left third of the backbone tree, whereas the cells from Day 3 embryos were found over the whole length of the backbone tree except for the first circle. Cells of some embryos were concentrated on two major circles (e.g. Day 2-E3 or Day 2-E6), while cells of other embryos were distributed over a broad range of circles (e.g. Day 3-E3 or Day 3-E2).

CellTree assumes that transcriptomes of cells contain a mixture of “topics” with per-topic gene distributions. It uses a Bayesian mixture model – the Latent Dirichlet Allocation (LDA) – to identify different topics. In our dataset, we identified six different topics present along the developmental pseudo-time line (Fig. 3b). Topic 1 was the most prominent, but was nearly absent in the penultimate phase of the pseudo-time line. Topic 2 was absent in the early phase, but predominantly present in the penultimate phase of the pseudo-time line. Topic 3 dominated the middle of the developmental pseudo-time line. Topics 4, 5 and 6 were less prominent and mainly observed in the first phase of the pseudo-time line. The biological roles of the six topics were also analysed by the CellTree tool. Gene set enrichment analysis was performed based on the org.Bt.eg.db genome-wide annotation with GO mapping^25^ and Bonferroni’s correction. The following GO terms were over-represented: Topic 1: “translation”, “cell division”; Topic 2: “translation”, “regulation of translational initiation”, “mRNA splicing, via spliceosome”, “cytoplasmic translation”, “rRNA processing”, “spliceosomal complex assembly”, “negative regulation of mRNA splicing, via splicing”, “cell division”, “regulation of alternative mRNA splicing, via spliceosome”; Topic 3: “translation”; Topic 4: “ATP synthesis coupled proton transport”; Topic 5: “mitochondrial translational elongation”; and Topic 6: “organic hydroxyl compound transport” (Supplementary Table S5).

### Major Embryonic Genome Activation (EGA) at the Single-Cell Level

In order to investigate when major EGA occurs in each blastomere, we analysed transcript levels of 129 genes that are actively transcribed at the eight-cell stage and whose mRNA is not present in earlier embryonic stages or oocytes^10^. In our dataset, transcripts of only 20 of these genes were detected. Each of these genes showed a unique expression pattern in blastomeres along the pseudo-time line (Supplementary Fig. S7) and blastomeres of individual embryos showed different transcript abundances (Supplementary Table S6). Interestingly, five Day 2 embryos and two Day 3 embryos had one blastomere each without UMI counts for any of these genes 20 genes.

### Analysis of Candidate Genes Inducing or Reflecting Cell Fate Decisions

**Figure 4:**
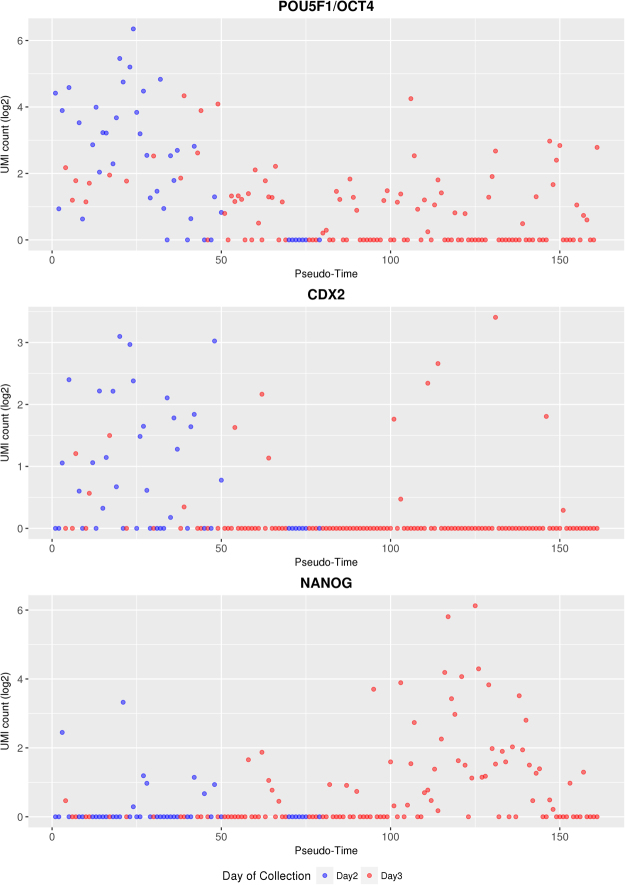
Changes in the transcript abundance of *POU5F1*/*OCT4*, *CDX2*, and *NANOG* along the pseudo-time line. Cells from Day 2 embryos are shown as blue symbols, cells from Day 3 embryos as red symbols.

**Figure 5:**
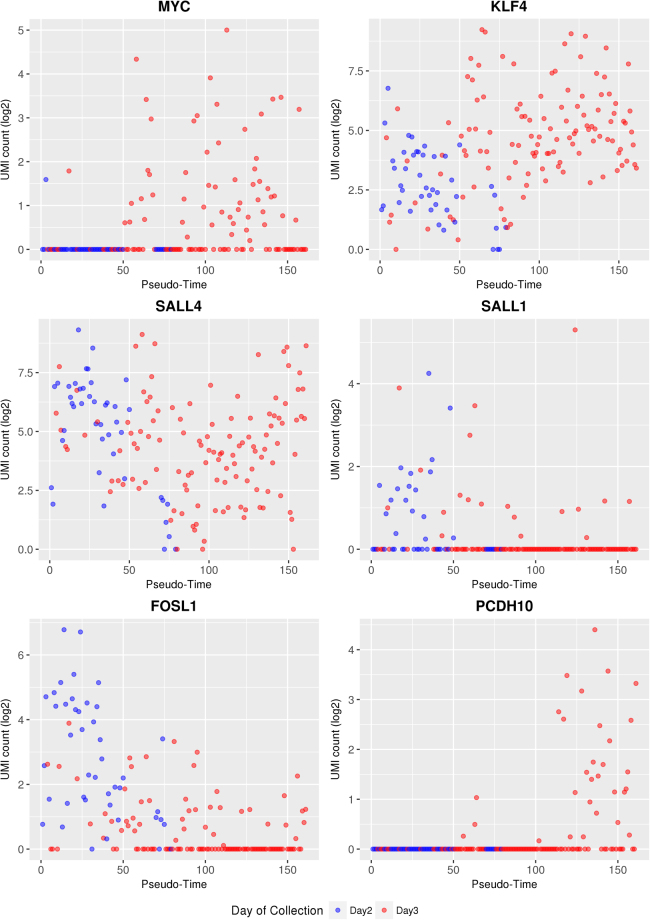
Changes in the transcript abundance of *MYC*, *KLF4*, *SALL4*, *SALL1, FOSL1*, and *PCDH10* as development proceeds. The UMI values of these genes are shown along the pseudo-time line. Cells from Day 2 embryos are shown as blue symbols, cells from Day 3 embryos as red symbols.

In order to study potential early cell lineage inclination events, we investigated the abundance of transcripts of genes known to be involved in early cell fate decisions. For each cell – ordered according to the pseudo-time line – the UMI counts for selected genes provided by the Drop-Seq pipeline were plotted (Figs 4, 5). A detailed list of the transcript abundance levels is provided in Supplementary Table S7.

The transcription factor POU5F1/OCT4 is involved in maintaining cell pluripotency of the inner cell mass in mouse embryos^26^. In bovine embryos, mRNA expression of *POU5F1/OCT4* was found in both the ICM and TE at the late blastocyst stage^27^, and its knockout was observed to be lethal at the second lineage differentiation in bovine embryos^28^. The homeobox gene *CDX2* regulates multiple trophoblast genes in bovine blastocysts^29^, but - in contrast to the situation in mouse embryos - CDX2 does not suppress *POU5F1/OCT4* expression^30^, but only down-regulates its level^29^*. NANOG* and *GATA6* are two key genes involved during the second lineage segregation in epiblast or primitive endoderm, respectively (reviewed in)^31^. *NANOG* transcripts were first observed at the eight-cell stage in the bovine embryo^10^, and its expression is required for the bovine embryonic development^28^. *GATA6* was observed to have acquired a species-specific ability to control trophoblast-specific gene expression in ruminant ungulates^32^.

In our study, *POU5F1*/*OCT4* transcripts were detected in more than 70% of the cells from Day 2 embryos and in about 50% of the cells from Day 3 embryos. *CDX2* transcripts were also detected in about 53% of the Day 2 blastomeres, but in a markedly lower proportion (12%) of the Day 3 blastomeres, although the positive cells contained relatively high levels of *CDX2* mRNA. *NANOG* transcripts were found in roughly 20% of the cells aligned in the first half of the pseudo-time line, and in a higher proportion (55%) and at higher levels in the more advanced half of the cells. *GATA6* mRNA was not detected in our dataset.

In addition to these key genes regulating the specification of the first embryonic cell lineages, we investigated additional candidate genes relevant for the maintenance of pluripotency or early differentiation events. UMI counts of the proto-oncogene *MYC*, which contributes to the selection of the epiblast cell pool^33^, were found in only one cell of Day 2 embryos, but in about half of the cells from Day 3 embryos. The proportion of *MYC* expressing blastomeres in individual Day 3 embryos ranged from 13% (2/15) to 100% (14/14). Transcripts of Krüppel like factor 4 (*KLF4*), which was observed to prevent differentiation of mouse ES cells and to regulate the expression of *Nanog*^34^, but not to be essential for early development^35^, were found in nearly all cells of Day 2 and Day 3 embryos with increasing abundance towards the end of the pseudo-time line. Similarly, transcripts of Sal-like 4 (*SALL4*), which is important for cell fate decision and required to maintain pluripotency of the inner cell mass in early mouse embryos^36^, were found in nearly all cells of Day 2 and Day 3 embryos, in various levels of abundance. Transcripts of Sal-like 1 (*SALL1*) were found in all embryos except for one Day 3 embryo, but only in a proportion of the blastomeres. In mouse ES cells, SALL1 is expressed in a differentiation-dependent manner and physically interacts with NANOG and SOX2 to regulate transcription^37^. Transcripts of *FOSL1*, which is required for development of the trophoblast lineage^38^, were detected in nearly all cells of the first third of the pseudo-time lime. In more advanced stages, the UMI count as well as the number of cells with detectable levels of *FOSL1* transcripts declined.

Moreover, we looked specifically at genes that were described to be predominantly expressed in either ICM or TE of bovine blastocysts^39^ and intersected this gene set with transcripts not detected before the eight-cell stage to exclude carry-over of maternal transcripts^10^. The intersection contained the predominantly ICM-expressed protocadherin-10 (*PCDH10*) gene. In our data set, *PCDH10* transcripts were found in 30 blastomeres of seven Day 3 embryos at the advanced end of the pseudo-time line (Fig. 5).

## Discussion

Single-cell RNA sequencing enables the study of heterogeneity in cell populations and paves a way for unprecedented analyses of developmental processes. Our study provides a comprehensive insight into developmental heterogeneity of blastomeres in bovine embryos at the time of major EGA (eight- to 16-cell stage). Previous studies of these developmental stages were performed with pools or individual bovine embryos^9,10^, while individual cells have not been analysed yet. In the present study, we used the SCRB-Seq approach to study the transcriptome of individual blastomeres derived from Day 2 and Day 3 embryos. The analysis itself destroys the embryos and therefore the developmental potential of the dissected embryo remains uncertain. A correlation between a combination of kinetic and morphological parameters and the rate of successful blastocyst formation was previously described for *in vitro*-fertilized human embryos^40^. A similar study described a novel system for selection of bovine IVF blastocysts for transfer to recipient animals by tracing the development of individual embryos with time-lapse cinematography and analysing embryo metabolism. This approach includes several kinetic and morphological prognostic factors, that span from the zygote to the blastocyst stage, and facilitate prediction of pregnancy success^13^. In the present study, we followed the development of 541 embryos by time-lapse microscopy in order to find parameters predictive for blastocyst formation. We found strong correlations between the timing of the first, second and third cleavages and the blastocyst formation rate^14^. These parameters were subsequently used for selecting developmentally competent embryos and excluding their low-grade counterparts.

SCRB-Seq^15^, a sophisticated procedure to construct 3′ specific UMI containing libraries from single cells, was used to sequence the transcriptomes of blastomeres of the selected embryos. cDNA reads were mapped to the bovine reference genome btau7 with the STAR tool^41^. Subsequently, data were normalized without using exogenous spike-ins, because technical variations do not affect spike-ins and endogenous transcripts uniformly, thus causing poorly normalized data^42^. We used UMI count^43^ instead of read count in order exclude duplicates originating from PCR amplification. Genes involved in the cell cycle^44^ were not excluded from our analysis as the variation between cells is largely explained by the sum of log expression values over all genes in a cell, rather than by cell cycle stage^45^.

Transcriptome data were used to cluster the cells and sort them along a pseudo-time line (from position 1 to position 161).

The clustering tool SC3^16^ identified six different clusters, and three of them contained cells from both Day 2 and Day 3 embryos. This finding indicates that at least some blastomeres of an embryo develop asynchronously. The number of 2,494 DAT hints to enormous changes of the transcriptome in that developmental period. This order of magnitude is comparable to the number of 2,940 DAT described by Graf *et al*.^10^ when comparing pools of ten eight-cell and 16-cell embryos. In addition to the DAT, the SC3 tool identified cluster specific markers genes. *FOLR1, NANOG* and *KLF5* were revealed as marker genes specific for cluster K4. In a previous study^10^, transcripts of the first two genes were not detected before the eight-cell stage, while *KLF5* transcripts are already present in the oocyte and embryonic transcription of this gene was detected at the four-cell stage. In contrast, transcripts of *RHBL1* (marker gene of cluster K2) and *CXCL1* (marker gene of cluster K5) were not detected in the previous study by Graf *et al*.^10^. Collectively, the results of this analysis suggest the presence of six different cell populations in early bovine embryos (Day 2 to Day 3). These cell populations are characterised by specific transcriptome signatures and comprise blastomeres of different embryos.

It is known that so-called dropouts (zero read counts for certain genes, due to failure of reverse transcription or low read counts) hamper single-cell transcriptome analyses. The clustering tool M3Drop^18^ focuses on genes not affected by dropouts, but cannot distinguish between technically caused dropouts and mRNA-species that naturally occur only in a certain proportion of the blastomeres. In our dataset, the M3Drop tool identified 15 genes unaffected by dropouts and clustered the cells based on these genes. In line with the clusters generated by the SC3 tool, some clusters contained cells from both Day 2 and Day 3 embryos.

In addition, the CellTree tool^24^ was used to sort cells based on their transcriptome in a time line (called “pseudo-time line”) and to build, based on the obtained time line, a backbone tree. Within the time line, blastomeres of some embryos (e.g. Day 2-E3: 9 cells) were located either in close vicinity or distributed over a broad range (e.g. Day 3-E3: 16 cells). This finding hints to an asynchronous development of blastomeres within an embryo. The linear structure of the backbone tree suggests that the first lineage differentiation towards ICM and TE has not occurred yet or is ongoing but below the detection level of single-cell RNA sequencing. Along the obtained pseudo-time line, different over-represented GO terms were identified. Their order suggested an orchestrated process of early development, starting with the GO terms “translation” and “cell division”. The GO term “cell division” then gradually disappeared, while the GO term “translation” and later also GO terms related to “RNA processing” became more prominent.

In order to study major EGA at the single-cell level, we investigated the abundance of 129 different transcripts that were first detected at the eight-cell stage in pooled embryos^10^, and detected 20 of these transcript species in our single-cell study. Five Day 2 embryos and two Day 3 embryos did not have detectable levels of any of these transcripts. This suggests that the timing of major EGA is neither synchronous among different embryos of the same stage nor among all blastomeres of one embryo.

Among the genes not transcribed before the eight-cell stage was *NANOG* that is involved in preventing differentiation of pluripotent cells. *NANOG* transcripts were detected in only 19% (8/43) of the Day 2 blastomeres, but in 43% (51/118) of the Day 3 blastomeres, reflecting a gradual and asynchronous activation of this gene in individual blastomeres. In contrast, *POU5F1*/*OCT4* and *CDX2* transcripts were revealed in 74% and 53% of the Day 2 blastomeres, while these proportions decreased to 50% and 12% in the Day 3 blastomeres, respectively. This is most likely due to degradation of maternal RNA that is apparently more pronounced for *CDX2* than for *POU5F1*/*OCT4*. The relatively high mRNA levels of *CDX2* in a proportion of the Day 3 blastomeres may hint to lineage inclination towards trophectoderm, although this was not evident from the backbone tree generated by the CellTree tool. An alternative explanation would be impaired maternal RNA degradation in a proportion of the blastomeres. Transcripts of the primitive endoderm marker gene *GATA6* were not detected in our study.

Transcripts of *MYC* that is involved in selecting the epiblast cell pool are already present in the oocyte^10^, but were detected in only one blastomere from a Day 2 embryo and in ~45% of the Day 3 blastomeres. This suggests rapid degradation of maternal *MYC* transcripts and embryonic activation of *MYC* towards the end of major EGA in about half of the blastomeres.

*KLF4* (necessary for preventing differentiation) and *SALL4* (involved in maintenance of pluripotency) are also present in oocytes and are thus detected before the eight-cell stage^10^. In the present study, blastomeres located at the end of the pseudo-time line showed higher transcript abundance of *KLF4*, suggesting increased embryonic transcription of this gene. The abundance levels of *SALL4* transcripts were high at the beginning and at the end of the pseudo-time line, but lower in the middle. This finding hints to initial degradation of maternal *SALL4* transcripts followed by active embryonic transcription of *SALL4*.

Embryonic transcription of *SALL1* (involved in pluripotency) and *FOSL1* (involved in TE development) is known to start at the 16-cell stage, although maternal transcripts of these genes were detected at earlier stages^10^. This explains the higher abundance of transcripts of the *SALL1* and *FOSL1* at the beginning of the pseudo-time line. Compared to *FOSL1*, the abundance of *SALL1* transcripts was on average lower and detected in a smaller proportion of blastomeres. Blastomeres with detectable levels of both transcripts were frequently found at the beginning of the pseudo-time line. Interestingly, in mouse embryonic stem cells, over-expression of the *Sall1* gene was observed to positively regulate the *Nanog* expression and thus prevent differentiation^37^. *FOSL1* is known to be important for invasive placentation, e.g. in human and mouse^46^. In our study of bovine embryos, the abundance of *FOSL1* transcripts was highest at the beginning and decreased towards the end of the pseudo-time line, which may be related to the late implantation and non-invasive, synepitheliochorial placentation in ruminants.

As an approach to detect potential early lineage inclination events in Day 2 to Day 3 embryos, we analysed genes that are known to be predominantly expressed in the ICM or TE of bovine blastocysts^39^. From this gene set, we selected the predominantly ICM-expressed protocadherin-10 (*PCDH10*) gene since its transcripts were not detected before the eight-cell stage^10^, thus avoiding confounding effects of maternal transcripts. *PCDH10* transcripts were detected in 30 blastomeres of 7 Day 3 embryos at the advanced end of the pseudo-time line, raising the possibility that these blastomeres may be determined towards ICM. However, the non-branched backbone tree revealed by the CellTree analysis of our data set argues against major lineage inclination events at the developmental stages investigated. Elegant aggregation experiments of labelled TE cells with blastomeres from 8-cell embryos revealed that TE cells can contribute to the ICM and its derivatives^30^, arguing against early lineage commitment in bovine embryos. In contrast, early lineage commitment and its relation to cell allocation have been observed in mouse embryos (reviewed in)^47,48^, thus underscoring the need for comparative embryological studies.

In summary, our study revealed heterogeneity of transcriptome profiles among single cells in bovine Day 2 and Day 3 embryos, suggesting asynchronous blastomere development during the phase of major embryonic genome activation.

## Material and Methods

*In vivo* procedures were conducted according to the German Animal Welfare Act (Tierschutzgesetz). Bull semen was donated by Bayern Genetik GmbH, Grub, Germany. Oestrous cow serum was donated by BFZF GmbH, Oberschleißheim, Germany. Bovine ovaries were obtained from a slaughterhouse (Münchner Schlachthof Betriebs GmbH, Munich, Germany). *In vitro* produced embryos were obtained from an EU approved bovine embryo collection and production centre at the Chair for Molecular Animal Breeding and Biotechnology of the LMU Munich (Moorversuchsgut Badersfeld, Oberschleißheim, Germany; approval number DE ETR 006 EWG).

### *In vitro* embryo production and single cell collection

Embryos were produced *in vitro* according to a standard procedure including *in vitro* maturation (IVM) and fertilization (IVF)^49^. Briefly, follicles from slaughterhouse ovaries were aspirated and obtained cumulus-oocyte complexes (COCs) were matured for 23 hours in modified Parker medium (MPM) supplemented with luteinising hormone (LH), follicle-stimulating hormone (FSH), and 5% oestrous cow serum (ECS). Matured COCs were co-incubated with sperm selected by the swim-up method after thawing. For IVM and IVF, COCs were incubated at 39 °C in a maximum humidified atmosphere of 5% CO_2_ in air. After 20 hours of co-incubation, presumptive zygotes were vortexed to remove remaining cumulus cells and transferred to synthetic oviductal fluid (SOF) supplemented with 5% ECS, 400 µl BME, 100 µl MEM under mineral oil and cultured at 5% CO_2_, 5% O_2_, 90% N_2_ and 39 °C in humidified air. At the time of collection (Table 1), embryos were transferred to drops of TALP-HEPES-PVP (THP)^50^ under oil for handling outside the incubator. All embryo manipulations were performed on a heated microscope plate set to 36 °C. The zona pellucida (ZP) was removed by treatment with 5 mg/ml pronase (Sigma Aldrich) for 1 min. Enzyme reaction was stopped by washing embryos in THP supplemented with 10% foetal calf serum (FCS) and the dissolved ZP was completely removed by gentle pipetting. Embryos were incubated in drops of PBS without Mg^2+^ and Ca^2+^ supplemented with 4 mg/ml polyvinylpyrrolidone under oil for 5–10 minutes and blastomeres were subsequently disaggregated by gentle pipetting^51^. Single cells were transferred individually to 0.5-µl drops of lysis buffer (Buffer A of Prelude Direct Lysis Module, NuGEN) under mineral oil, collected in a 384-well plate, and stored at −80 °C.

### Timing of early cleavages as a predictive parameter for blastocyst formation

A total of n = 541 zygotes generated by *in vitro* fertilization as described before were transferred into an embryo monitoring system (Primo Vision, Vitrolife, Gothenburg, Sweden) and images were recorded every 5 minutes for 168 hours and analysed with Primo Vision Analyzer software. Timing of the first, second and third cleavage, i.e. development until eight-cell stage, was evaluated and correlated to the formation of a blastocyst with logistic regression analysis. Statistical analysis were performed with SPSS 18.00 and p-values less than 0.05 were considered as significant^14^.

### Single-cell RNA-seq library preparations

Sequencing libraries were constructed according to the Single-Cell RNA Barcoding and Sequencing (SCRB-Seq) protocol^15^. Briefly, the 384-well plate containing the lysed blastomeres was thawed and an RT master mix supplemented with 0.1 µl of diluted (1:10^6^) ERCC RNA Spike-In Mix (Life Technologies) was added to each well. A polyT anchor containing a cell barcodes (6 nt) and the Unique Molecular Identifiers (UMIs; 10 nt) was used to prime cDNA synthesis in a template switching reaction with Maxima H Minus Reverse Transcriptase (Thermo Scientific). After cDNA synthesis, samples were pooled and unused barcode primers were removed by digestion with exonuclease I (New England Biolabs). Full-length cDNA amplification was performed with the KAPA HiFi HotStart polymerase (KAPA Biosystems). Nextera XT libraries were constructed from 1 ng of pre-amplified cDNA according to the instruction of the manufacturer and finally amplified with a custom P5 primer (IDT). The libraries were sequenced paired-end with 16 cycles to decode cell barcodes and UMI from read 1 and 50 cycles for read 2 to sequence the cDNA fragment.

### Basic Data Processing and Sequence Alignment

SCRB-Seq libraries were demultiplexed based on Nextera barcodes and cell barcodes. All reads were mapped to Bos tau7 (UCSC) and ERCC spike-in reference. Alignments were calculated using STAR 2.5.2b with default parameters. UMI tables were generated using the published Drop-seq pipeline^52^.

### Data Filtering and Normalization

Cells with less than 2,000 UMI were removed (Supplementary Fig. S2). Genes that showed no expression more than 10% of the cells were removed. Data were then normalised to account for differences in efficiency of transcript recovery between wells: gene specific UMI counts were divided by the total number of UMI counts per blastomere and then multiplied by the median of total UMI counts across all blastomeres.

### Clustering analysis

Two unsupervised hierarchical clustering analyses were performed on the filtered and normalised data. In order to investigate the transcripts in single cells, Single-Cell Consensus Clustering (SC3) version 1.4.2, R package^16^ was used. The required number of clusters was calculated after testing the significance of the eigenvalues of the matrix of covariance, by using the Tracy-Widom test^17^. The default parameters were used. A second hierarchical clustering tool was used that excludes genes affected by dropouts. The Michaelis-Menten Model (M3Drop) R package^18^ relies on the Michaelis-Menten equation to model the relationship between the frequencies of dropouts and the expression level of genes. Significant outliers from the Michaelis-Menten equation are identified after performing a Z-test between the estimated K (mean expression level required for a gene to be detected in 50% of the cells) and the fitted K_M_ (FDR = 1%, p = 0.05). The significant outliers, also called differentially expressed genes, are the genes not affected by drop-outs and are then used for identifying cell sub-populations by using Ward’s hierarchical clustering. The default parameters were used.

### Biological Pseudo-Time

In order to align the blastomeres according to their developmental progress, rather than by the time they were collected, we used the R package CellTree^24^ on the log2(UMI + 1) transformed data. Briefly, CellTree assumes that cells belong to a temporal continuum and assigns each cell a biological “pseudo-time” to form a “pseudo-time line” along which they can be ordered. This is performed by computing a matrix of pairwise distances (chi-square distance) and assuming that intragroup variance increases as development proceeds. According to such order, CellTree produces tree structures showing the hierarchical relationship between single-cell samples. It identifies groups of genes (called “topics”), by using the Bayesian mixture model - the Latent Dirichlet Allocation (LDA), and the topic-associated gene ontology terms.

### Data availability

The dataset generated for the current study is available in GEO repository under accession number GSE99210.

## Electronic supplementary material

Supplementary Information

Supplementary Table S1

Supplementary Table S2

Supplementary Table S3

Supplementary Table S4

Supplementary Table S5

Supplementary Table S6

Supplementary Table S7
