# Supplementary material for: Single-cell RNA sequencing reveals developmental heterogeneity of blastomeres during major genome activation in bovine embryos
Source: Sci Rep. 2018 Mar 6;8:4071. doi: 10.1038/s41598-018-22248-2 (PMC5840315; doi:10.1038/s41598-018-22248-2)
Supplement: Supplementary file 1 — Supplementary Information [file 41598_2018_22248_MOESM1_ESM.pdf]

Supplementary Materials

Single-cell RNA sequencing reveals developmental heterogeneity of blastomeres during major genome activation in bovine embryos

Ilaria Lavagi, Stefan Krebs, Kilian Simmet, Andrea Beck,
Valeri Zakhartchenko, Eckhard Wolf*, Helmut Blum*

*equal contribution

**Content**	**Page**
Supplementary Figure S1	2
Supplementary Figure S2	3
Supplementary Figure S3	4
Supplementary Figure S4	5
Supplementary Figure S5	6
Supplementary Figure S6	7
Supplementary Figure S7a	8
Supplementary Figure S7b	9
Supplementary Table S1	10
Supplementary Table S2	14
Supplementary Table S3	64
Supplementary Table S4	66
Supplementary Table S5	66
Supplementary Table S6	67
Supplementary Table S7	76

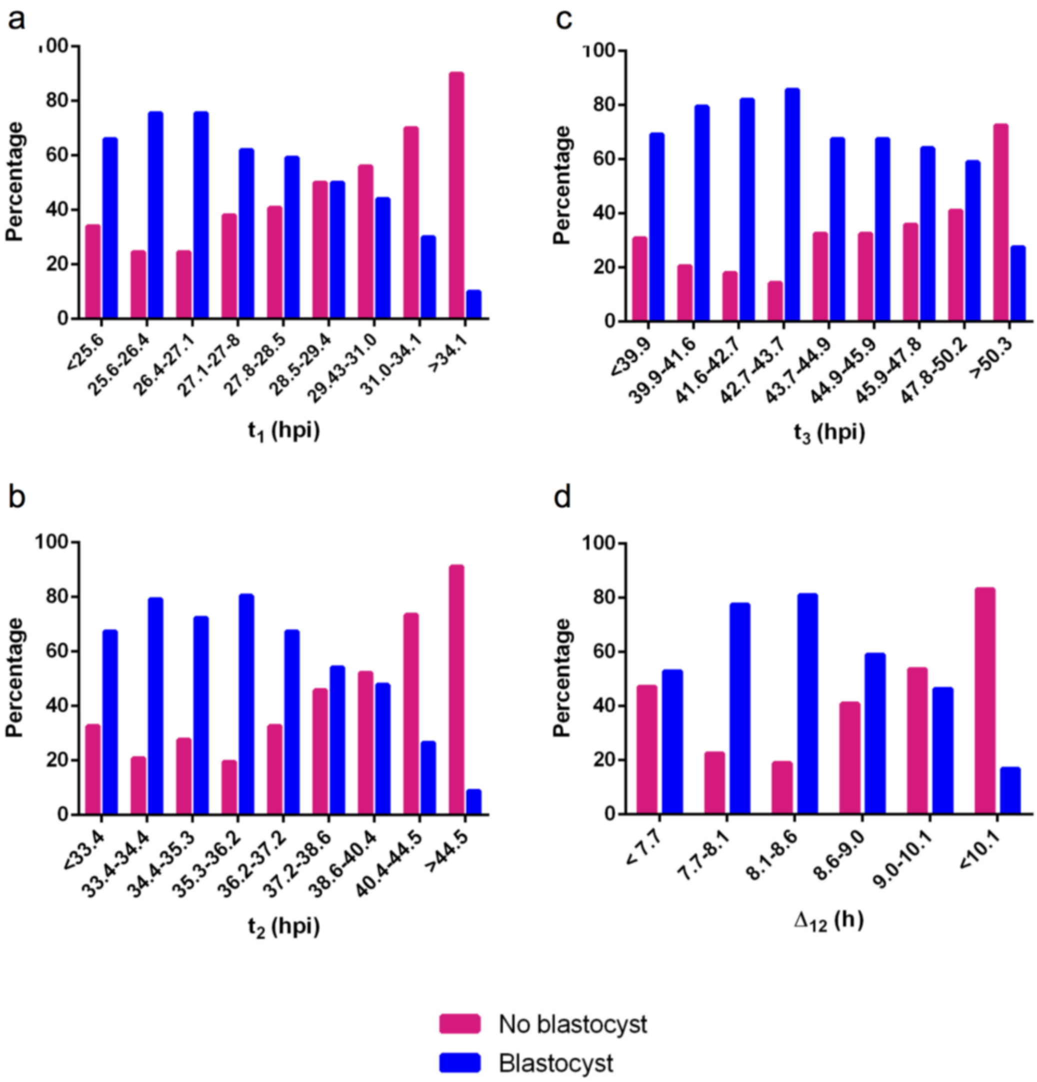

Supplementary Figure S1. (a) Early timing of the first cleavage had a positive effect on the blastocyst rate (optimal time range: 25.6-27.1 hpf). **(b)** Early timing of the second cleavage had a positive effect on the blastocyst rate (optimal time range: 33.4-36.2 hpf). **(c)** Early timing of the third cleavage had a positive effect on the blastocyst rate (optimal time range: 34.0-43.7 hpf). **(d)** Very short and very long time between the first and second cleavage had a negative effect on the blastocyst rate (optimal time range: 7.7-8.6 hpf). hpf = hours post fertilization.

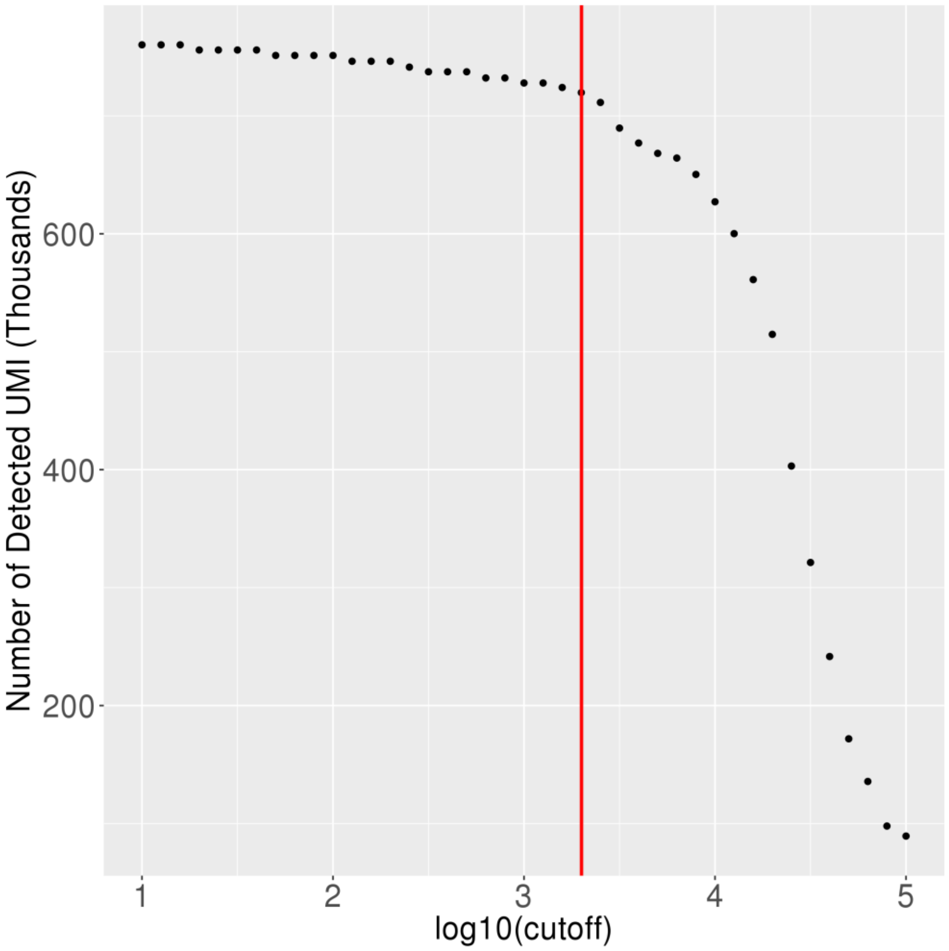

Supplementary Figure S2. Sum of the number of UMI counts detected across all cells with respect to the total UMI cut-off. The UMI cut-off is represented in log10 scale. The cut-off was set to 3.4 (~ 2,000 UMI).

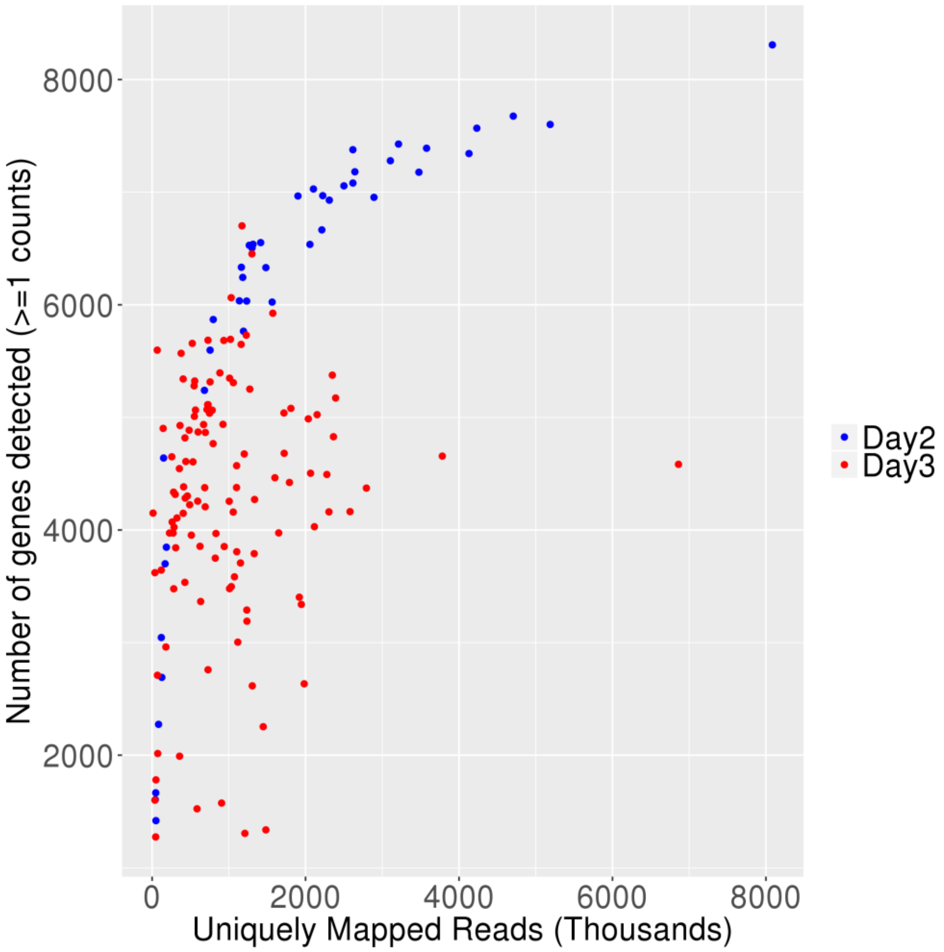

Supplementary Figure S3. Saturation Plot. Number of uniquely mapped reads vs. number of genes with detected transcripts (>=1 counts). Data points are represented in blue for cells from Day 2 embryos and in red for cells from Day 3 embryos.

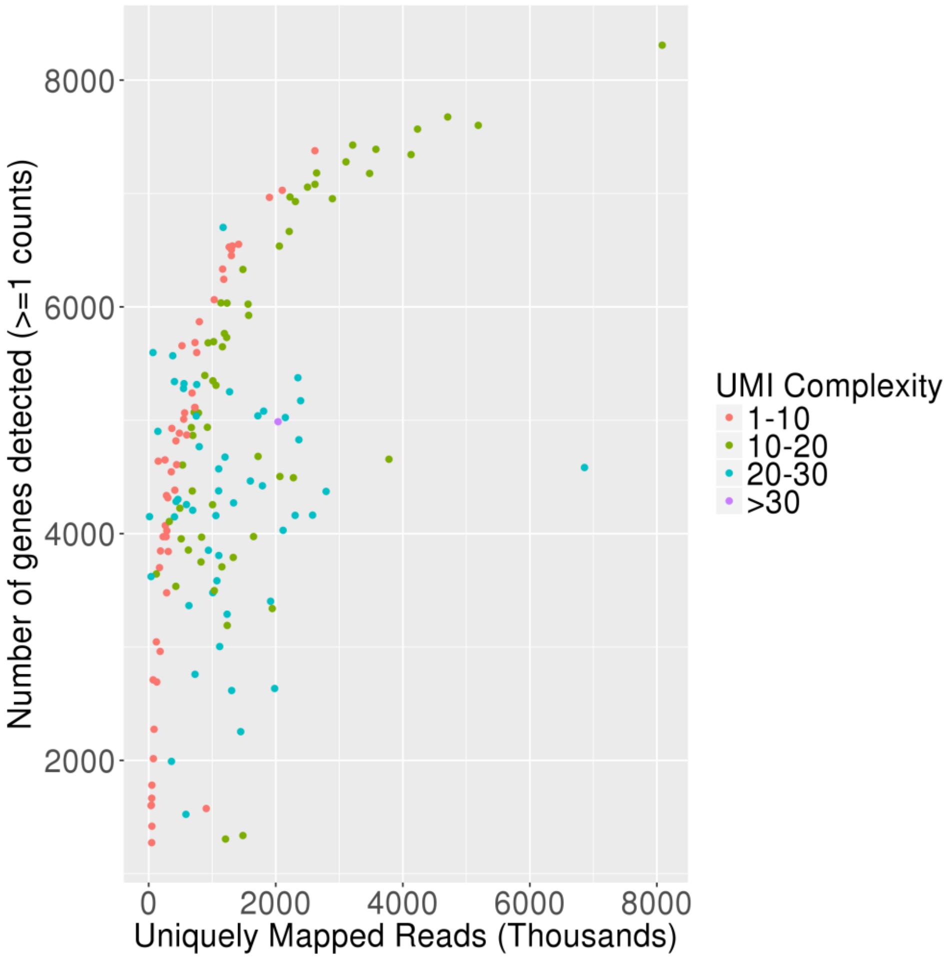

Supplementary Figure S4. Saturation Plot. Number of uniquely mapped reads vs. number of genes detected (>=1 counts). Data points are represented in different colours according to the UMI complexity. The UMI complexity corresponds to the ratio between all counted molecular identifiers (MIs) and the number of unique molecular identifiers (UMIs).

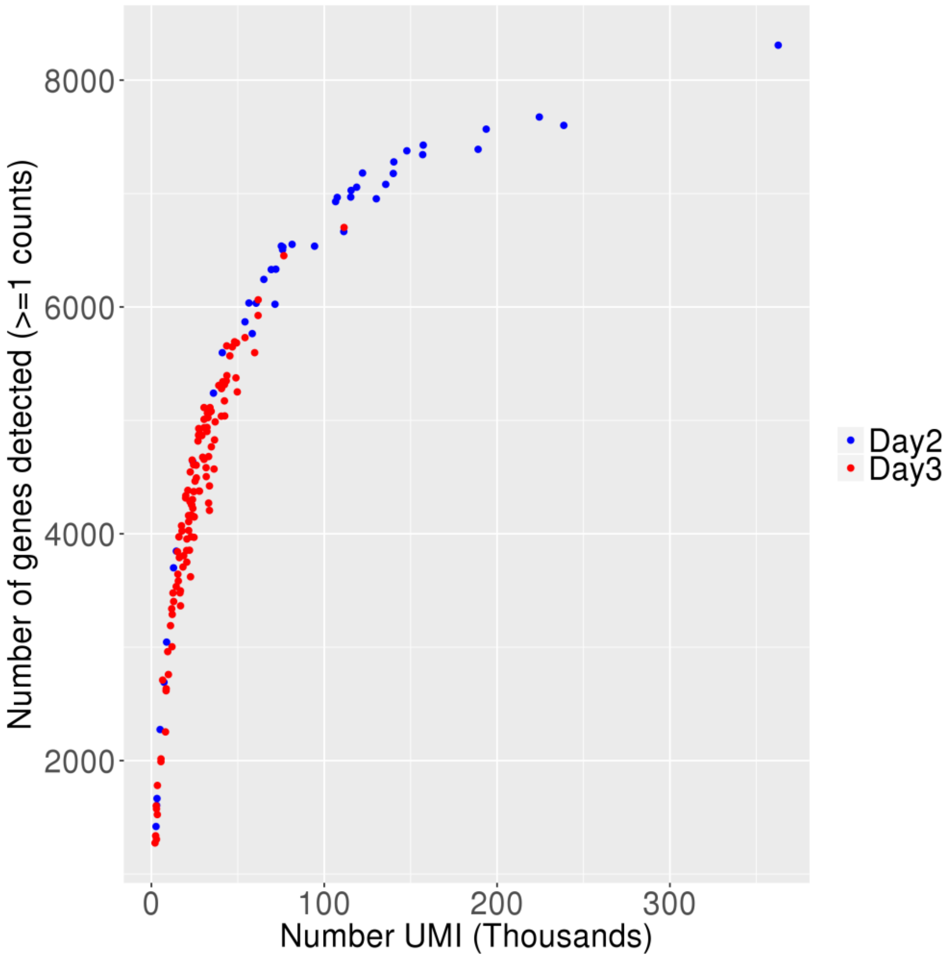

Supplementary Figure S5. Saturation Plot. Number of UMI vs. number of genes with detected transcripts (>=1 counts). Data points are represented in blue for cells from Day 2 embryos and in red for cells from Day 3 embryos.

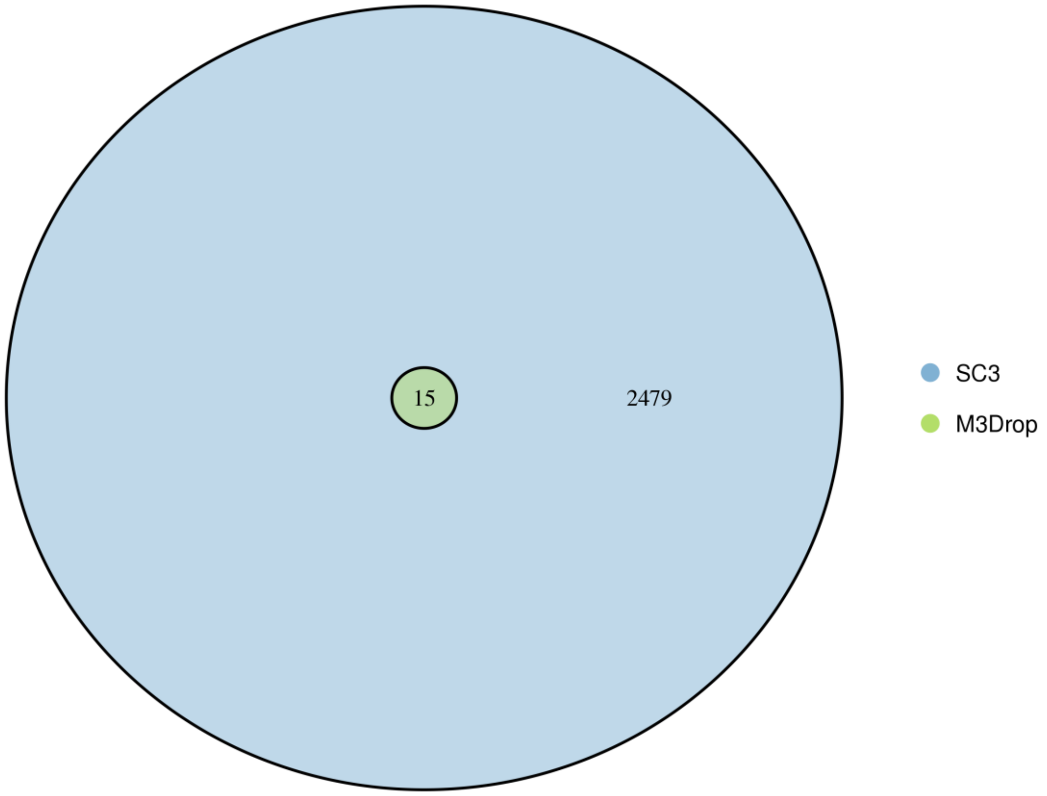

Supplementary Figure S6. Venn diagram comparing the 2,494 differentially abundant transcripts (DAT), obtained by using SC3 pipeline, with the 15 genes not affected by drop-outs, obtained by using M3Drop approach.

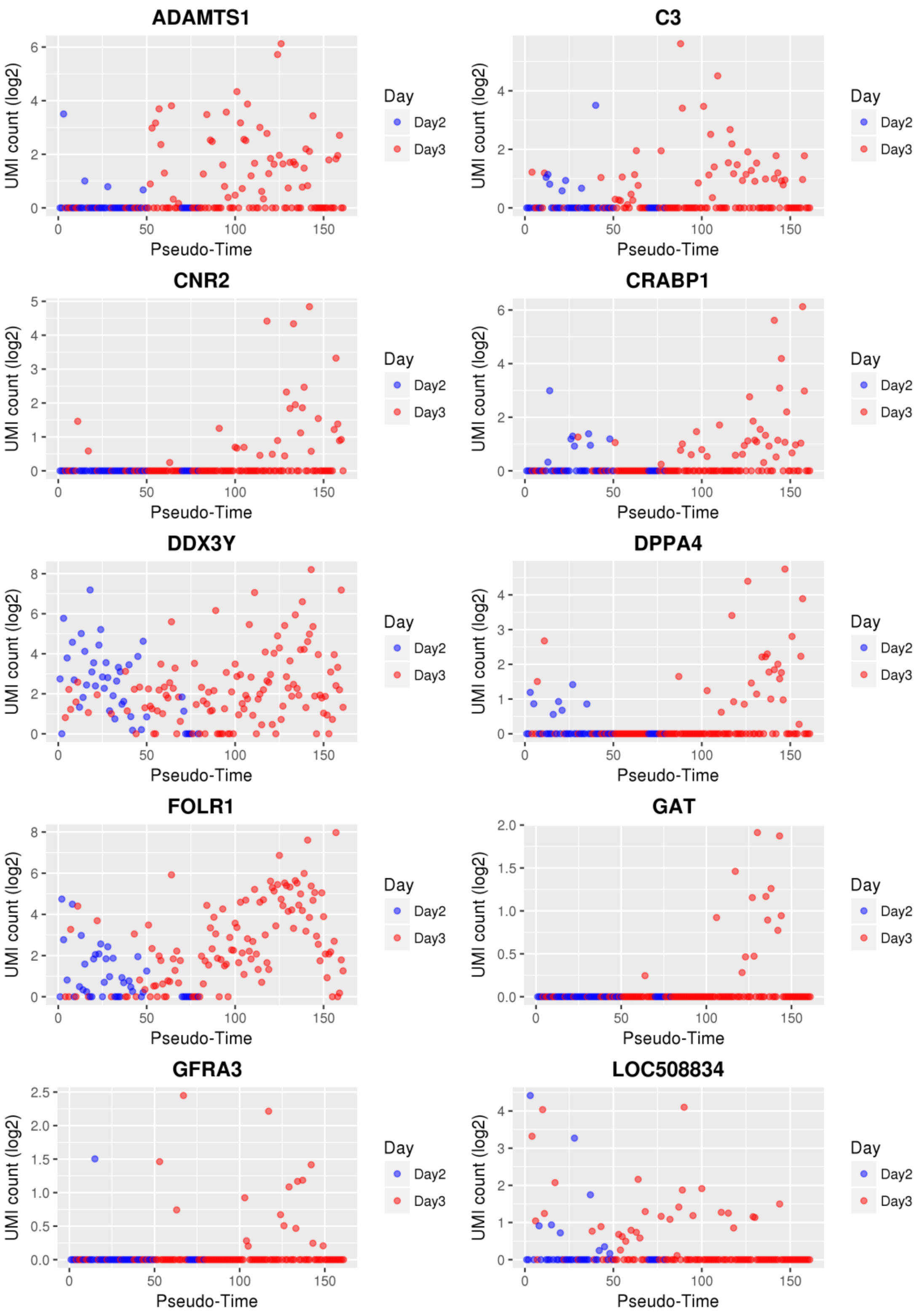

Supplementary Figure S7a. Transcript abundance of the genes first actively transcribed at major embryonic genome activation (EGA). The single cells are aligned according to the pseudo-time line.

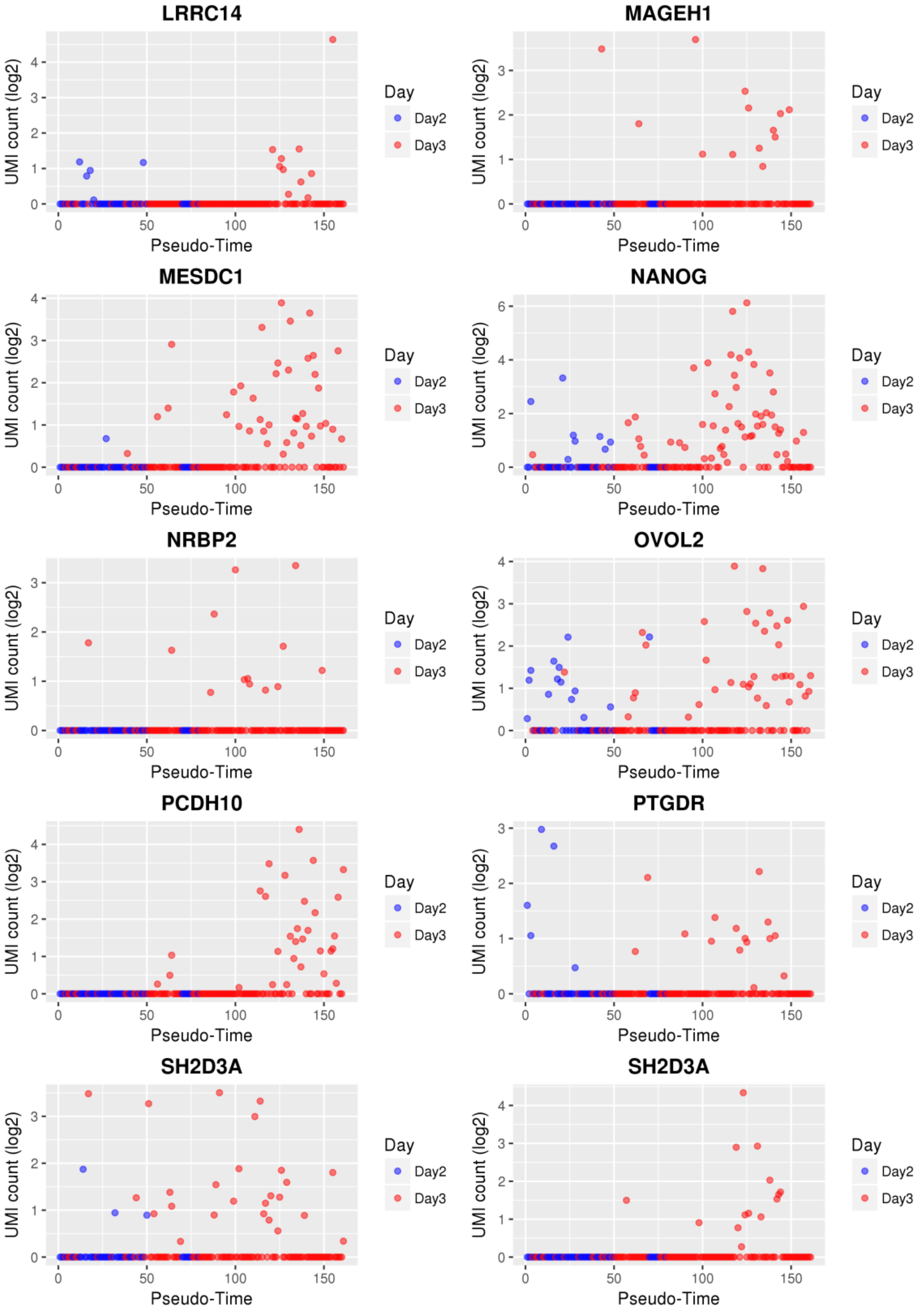

Supplementary Figure S7b. Transcript abundance of the genes first actively transcribed at major embryonic genome activation (EGA). The single cells are aligned according to the pseudo-time line.

Supplementary Table S1. Row count. Information on the count of generated reads, uniquely mapped reads, unique molecular identifiers (UMIs), molecular identifiers (MIs), and detected genes.

Library Name	No. reads	% kept reads after filtering	No. uniquely mapped reads	No. UMI	No. genes detected	All UMI
E1.1_p3	4.309.219	89%	2.617.309	135.584	7.081	1.529.101
E1.2_p3	3.053.922	90%	2.056.708	94.401	6.536	1.207.649
E1.3_p3	88.737	87%	47.586	3.230	1.666	23.673
E1.4_p3	67.384	83%	28.105	1.501	997	8.783
E1.5_p3	80.205	85%	41.752	3.019	1.605	20.148
E1.6_p3	548.917	88%	186.014	14.445	3.847	101.070
E1.7_p3	7.874.226	90%	4.707.109	224.445	7.675	2.835.856
E14.1_p3	12.610.686	90%	8.084.065	362.668	8.308	4.244.672
E14.2_p3	249.717	86%	120.012	8.800	3.045	51.591
E14.3_p3	3.501.574	90%	2.102.756	115.550	7.028	1.126.286
E14.4_p3	2.412.252	90%	1.482.867	69.337	6.330	757.771
E14.5_p3	2.577.675	91%	1.315.563	75.167	6.537	698.945
E14.6_p3	1.888.132	90%	1.232.652	60.590	6.033	721.484
E14.7_p3	3.528.126	91%	2.309.520	106.574	6.929	1.207.219
E14.8_p3	4.520.663	90%	2.642.867	122.157	7.181	1.376.819
E14.9_p3	352.989	88%	169.544	12.803	3.700	83.270
E16.1_p3	6.772.236	90%	4.232.149	193.714	7.568	2.264.002
E16.2_p3	4.167.351	89%	2.500.150	118.750	7.056	1.350.425
E16.3_p3	40.178	78%	17.930	615	481	2.413
E16.4_p3	2.649.490	90%	1.266.674	76.074	6.528	667.004
E16.5_p3	17.830	85%	9.177	52	48	141
E16.6_p3	3.612.133	91%	2.224.111	115.331	6.969	1.225.211
E16.7_p3	4.679.685	90%	2.616.788	147.803	7.377	1.363.480
E16.8_p3	2.357.782	90%	1.302.557	75.886	6.505	691.882
E16.9_p3	1.241.578	89%	755.650	41.019	5.597	400.440
E11.1_p3	1.367.911	88%	796.982	54.145	5.869	440.236
E11.2_p3	3.483.479	91%	2.212.214	111.307	6.665	1.278.410
E11.3_p3	1.834.149	90%	1.190.849	58.345	5.765	655.806
E11.4_p3	8.352.228	90%	5.188.133	238.601	7.601	3.000.471
E11.5_p3	4.364.058	90%	2.891.704	130.121	6.954	1.642.287
E11.6_p3	31.107	85%	18.447	261	216	1.045
E11.7_p3	100.289	84%	50.032	2.666	1.419	15.293
E11.8_p3	2.392.645	90%	1.564.471	71.512	6.024	848.358
E14.1_p2	5.423.343	90%	3.577.154	189.025	7.390	2.006.453
E14.2_p2	5.278.511	89%	3.211.369	157.273	7.427	1.725.599
E14.3_p2	1.752.728	90%	1.137.290	56.431	6.035	589.384
E14.4_p2	1.803.850	90%	1.163.582	71.983	6.333	601.206
E14.5_p2	1.861.393	89%	1.181.417	65.054	6.243	613.073
E14.6_p2	4.584.879	89%	3.105.469	140.255	7.279	1.667.861
E14.7_p2	2.986.164	90%	1.900.664	107.522	6.966	1.060.522
E14.8_p2	210.133	90%	84.180	5.029	2.274	14.808
E15.1_p2	388.988	82%	149.687	23.892	4.639	62.059
E15.2_p2	6.540.522	91%	4.130.170	156.960	7.343	2.348.421

E15.3_p2	5.306.400	90%	3.477.480	139.949	7.177	1.977.386
E15.4_p2	232.592	88%	126.840	7.275	2.691	59.799
E15.5_p2	1.087.197	90%	682.145	35.909	5.240	358.261
E15.6_p2	2.140.465	90%	1.415.147	81.403	6.552	812.163
E2.1_p1	2.017.591	91%	1.301.448	76.616	6.452	698.734
E2.2_p1	1.438.567	91%	1.009.960	43.309	5.348	585.032
E2.3_p1	1.530.360	91%	1.021.546	48.109	5.693	549.746
E2.4_p1	2.394.571	89%	1.573.168	61.729	5.925	908.156
E2.5_p1	1.194.954	91%	715.114	33.663	5.070	372.875
E2.6_p1	1.041.759	91%	693.928	29.184	4.866	360.359
E2.7_p1	1.349.760	90%	934.746	49.264	5.683	500.989
E2.8_p1	7.580	77%	3.600	131	106	462
E2.9_p1	1.687.878	90%	1.030.766	61.780	6.063	527.843
E2.10_p1	1.806.208	91%	1.160.519	46.836	5.648	611.734
E2.11_p1	1.785.101	90%	1.226.962	54.151	5.730	705.344
E2.12_p1	87.483	88%	46.516	2.101	1.274	19.796
E2.13_p1	870.011	89%	532.522	25.916	4.605	272.246
E2.14_p1	1.394.605	90%	883.201	43.669	5.395	490.327
E2.15_p1	1.232.789	91%	783.593	32.377	5.064	394.694
E3.1_p1	484.659	88%	301.101	19.806	4.316	143.636
E3.2_p1	856.134	89%	410.829	21.090	4.383	173.911
E3.3_p1	63.987	88%	36.701	2.794	1.601	10.888
E3.4_p1	80.521	90%	11.188	17	15	18
E3.5_p1	845.288	88%	482.586	27.858	4.886	212.247
E3.6_p1	1.553.963	89%	1.058.614	38.977	5.308	459.240
E3.7_p1	620.395	89%	427.105	14.349	3.535	149.818
E3.8_p1	528.305	88%	307.432	15.121	3.842	143.592
E3.9_p1	426.958	87%	281.918	12.473	3.478	117.755
E3.10_p1	1.237.362	89%	729.772	30.439	5.113	256.207
E3.11_p1	742.877	89%	439.513	24.431	4.608	187.921
E3.12_p1	448.514	89%	261.059	17.415	4.071	120.318
E3.13_p1	989.028	90%	598.738	27.275	4.870	213.488
E3.14_p1	525.728	89%	279.837	19.880	4.336	112.326
E3.15_p1	44.312	86%	22.804	1.608	1.100	4.095
E3.16_p1	721.139	90%	363.933	27.384	4.928	134.324
E10.1_p1	678.898	91%	428.182	26.960	4.818	189.882
E10.2_p1	135.710	86%	69.621	6.504	2.710	29.734
E10.3_p1	450.136	90%	274.323	15.936	3.973	126.464
E10.4_p1	872.973	90%	523.998	43.565	5.657	230.927
E10.5_p1	456.202	90%	285.617	17.719	4.025	149.398
E10.6_p1	381.453	91%	226.686	15.954	3.973	100.629
E10.7_p1	934.996	90%	551.167	30.459	5.009	250.204
E10.8_p1	855.873	91%	565.245	32.500	5.065	255.466
E10.9_p1	603.654	90%	355.595	22.487	4.545	152.345
E10.10_p1	1.159.038	90%	728.391	48.477	5.685	326.292
E10.11_p1	276.785	89%	179.612	9.443	2.961	85.648
E10.12_p1	1.115.059	90%	723.902	33.903	5.113	314.641

E10.13_p1	711.883	91%	489.964	23.969	4.224	267.040
E10.14_p1	540.288	89%	256.378	23.578	4.650	98.472
E10.15_p1	1.055.395	90%	671.050	30.536	4.936	307.538
E10.16_p1	504.032	88%	73.522	5.543	2.015	29.192
E16.1_p1	978.007	87%	633.021	16.849	3.365	357.514
E16.2_p1	263.699	92%	50.136	3.472	1.781	19.789
E16.3_p1	1.962.716	91%	1.005.093	23.364	4.255	432.581
E16.4_p1	3.551.203	90%	2.348.935	48.907	5.375	1.138.934
E16.5_p1	1.730.919	91%	1.057.360	23.695	4.159	528.138
E16.6_p1	2.057.572	88%	1.100.607	27.582	4.377	576.931
E16.7_p1	1.410.131	90%	823.233	20.479	3.750	393.791
E16.8_p1	1.547.770	89%	1.009.226	16.546	3.479	346.615
E16.9_p1	3.278.847	89%	2.115.466	21.560	4.029	499.113
E16.10_p1	2.353.477	91%	1.273.060	49.694	5.251	1.101.034
E16.11_p1	5.352.561	91%	3.782.308	30.466	4.656	592.325
E16.12_p1	126.296	88%	66.982	59.744	5.597	1.657.147
E16.13_p1	2.547.637	92%	1.482.645	2.454	1.337	31.906
E6.1_p3	3.313.306	92%	2.150.788	32.716	5.024	823.510
E6.2_p3	583.519	90%	378.301	45.381	5.569	1.169.480
E6.3_p3	2.100.105	91%	1.305.781	8.492	2.616	188.154
E6.4_p3	2.479.727	92%	1.600.455	25.255	4.464	685.815
E6.5_p3	3.079.072	92%	1.808.938	34.583	5.080	847.247
E6.6_p3	630.145	90%	404.914	41.288	5.341	975.402
E6.7_p3	2.961.826	92%	1.982.352	8.565	2.634	200.862
E6.8_p3	1.062.727	91%	555.389	41.374	5.323	1.109.401
E6.9_p3	2.657.791	91%	1.330.700	16.169	3.790	281.977
E6.10_p3	230.403	90%	144.143	32.160	4.902	724.045
E6.11_p3	915.062	90%	585.769	3.418	1.524	70.076
E6.12_p3	202.696	89%	119.715	15.307	3.644	305.049
E6.13_p3	1.824.904	92%	1.209.171	2.924	1.306	53.800
E6.14_p3	1.063.553	91%	431.226	22.301	4.283	600.025
E6.15_p3	2.859.582	91%	1.945.259	11.750	3.339	226.671
E6.16_p3	1.050.413	91%	547.533	40.572	5.280	1.087.451
E6.17_p3	2.078.362	91%	1.234.388	12.049	3.289	275.806
E13.1_p3	972.669	90%	594.197	23.187	4.256	582.905
E13.2_p3	2.951.080	92%	1.918.743	12.871	3.403	283.369
E13.3_p3	2.013.883	90%	1.201.116	29.662	4.675	772.380
E13.4_p3	3.617.751	91%	2.579.678	22.733	4.163	558.663
E13.5_p3	2.372.234	91%	1.718.379	42.413	5.039	1.240.214
E13.6_p3	681.274	90%	405.259	24.742	4.148	705.386
E13.7_p3	1.762.899	89%	1.236.018	11.054	3.190	190.909
E13.8_p3	4.024.786	91%	2.792.992	24.503	4.372	576.064
E13.9_p3	3.348.857	91%	2.391.799	42.248	5.172	1.226.338
E13.10_p3	3.211.189	91%	2.363.796	36.518	4.828	1.083.887
E13.11_p3	3.114.785	92%	2.035.993	36.888	4.987	1.142.351
E13.12_p3	9.530.527	91%	6.860.570	31.623	4.583	925.047
E13.13_p3	1.673.906	90%	1.171.403	111.463	6.701	3.310.101

E13.14_p3	1.527.381	91%	940.115	20.368	3.853	570.780
E6.1_p2	458.157	90%	324.448	21.557	4.107	418.933
E6.2_p2	544.509	87%	358.015	5.513	1.991	142.990
E6.3_p2	2.156.427	90%	1.448.085	8.154	2.253	177.198
E6.4_p2	19.459	85%	12.502	24.784	4.150	604.287
E6.5_p2	1.820.218	89%	1.131.162	209	165	3.544
E6.6_p2	711.367	89%	459.833	23.777	4.302	534.044
E6.7_p2	1.059.032	90%	728.727	9.834	2.759	210.865
E6.8_p2	1.753.787	89%	1.116.952	11.908	3.004	338.414
E6.9_p2	1.400.226	89%	923.803	32.188	4.938	498.505
E6.10_p2	1.798.549	90%	1.103.207	18.695	3.807	431.391
E6.11_p2	3.138.766	91%	2.277.734	26.017	4.493	448.929
E6.12_p2	1.176.448	89%	795.060	34.686	4.767	1.033.905
E6.13_p2	1.810.894	89%	1.073.477	15.574	3.584	386.426
E6.14_p2	3.535.062	89%	2.304.901	21.489	4.161	519.083
E6.15_p2	1.272.423	88%	755.965	42.258	5.315	914.185
E6.16_p2	1.082.173	87%	624.189	22.028	3.855	409.371
E7.1_p2	1.587.929	89%	1.032.524	16.790	3.497	322.032
E7.2_p2	1.485.246	87%	832.310	24.650	3.969	480.199
E7.3_p2	60.025	85%	36.780	22.632	3.621	469.965
E7.4_p2	268.611	89%	55.051	958	614	14.859
E7.5_p2	1.448.848	87%	905.615	2.928	1.575	18.495
E7.6_p2	2.578.354	89%	1.650.081	23.001	3.974	430.839
E7.7_p2	2.994.008	89%	1.790.120	33.598	4.422	755.616
E7.8_p2	2.337.832	88%	1.335.756	33.096	4.271	765.655
E7.9_p2	1.088.741	87%	692.077	33.586	4.206	745.095
E7.10_p2	841.510	86%	511.576	20.540	3.954	308.329
E7.11_p2	1.778.504	89%	1.152.046	18.256	3.707	254.304
E7.12_p2	2.548.699	89%	1.721.453	33.107	4.681	576.902
E7.13_p2	1.765.228	88%	1.101.797	36.242	4.571	788.034
E7.14_p2	3.000.717	90%	2.065.209	31.731	4.504	565.914
E7.15_p2	1.246.208	89%	748.377	40.442	5.037	922.306
E7.16_p2	1.163.853	90%	685.734	27.805	4.376	365.664

Supplementary Table S2. 2,494 genes with significantly ($p < 0.01$) differently abundant transcripts (DAT) identified by using the SC3 pipeline.

Gene	sc3_6_markers_clusts	sc3_6_de_padj
DUSP1	4	3,19163E-17
UCHL1	1	3,46226E-15
PYCR2	4	3,6645E-15
MRPL17	4	3,69048E-15
BTG1	4	4,19081E-15
RNF11	4	4,49954E-15
XRCC2	1	1,48411E-14
SNHG12	4	3,15744E-14
LOC100196901	4	3,70682E-14
NETO1	1	9,00088E-14
PRNP	4	1,67764E-13
RPS19	4	1,75244E-13
COG2	1	1,91013E-13
ZNF2	1	2,18468E-13
TFAP2C	4	2,65079E-13
RACK1	4	2,80186E-13
PIP5K1A	4	3,69806E-13
SDS	4	3,70259E-13
TRIP4	1	4,26628E-13
ITPR1	5	5,12539E-13
RUFY2	1	6,73613E-13
RABGEF1	1	7,47336E-13
ARFGAP3	4	7,53523E-13
PRPS1	4	7,74517E-13
DYNC2LI1	3	8,85953E-13
SLC25A25	4	9,1132E-13
TPMT	1	9,80319E-13
GALNT13	1	1,11108E-12
RPL36AL	4	1,13246E-12
VKORC1	4	1,64992E-12
NUDT12	1	2,08732E-12
ARMT1	4	4,39041E-12
PLS1	1	4,67168E-12
FOLR1	4	5,56843E-12
TKT	4	7,98055E-12
AMIGO2	4	8,70221E-12
RPL26	4	9,06242E-12
HNRNPAB	4	9,50205E-12
TSEN54	4	9,50988E-12
GRO1	5	9,63135E-12
RHEBL1	2	1,4103E-11
CCNC	4	1,43501E-11
IL18	4	1,47224E-11
RPRD1A	4	1,66394E-11
DTWD1	1	2,19515E-11
PDE6C	1	2,33296E-11
COL1A1	4	2,6209E-11

DDX17	4	2,71957E-11
MIER2	1	2,95389E-11
PARP14	4	3,07252E-11
KDELC1	5	3,1476E-11
ERICH1	4	3,15137E-11
TIGAR	1	3,25596E-11
HAX1	4	3,4073E-11
LOC782781	4	4,24316E-11
C1H3orf58	4	4,78934E-11
PRR5	4	5,71604E-11
CCDC126	4	6,09051E-11
C1D	4	6,4692E-11
C8H9orf64	1	6,70047E-11
CEPT1	1	7,40371E-11
ANK3	1	7,47493E-11
CDV3	4	8,17086E-11
RASA1	1	8,61917E-11
PNP	4	8,78953E-11
DIABLO	4	1,06967E-10
F3	1	1,26512E-10
TMEM128	1	1,43898E-10
SNAPC1	4	1,47879E-10
SLC35D1	1	1,49719E-10
DPP4	1	1,5061E-10
TYW3	4	1,53583E-10
PSMA4	1	1,84571E-10
USP13	1	1,85677E-10
TIMM10B	4	2,07667E-10
ACAA1	1	2,09553E-10
ITM2B	4	2,16148E-10
YTHDF2	4	2,2118E-10
CREM	3	2,31335E-10
RSRP1	2	2,63661E-10
FAM207A	4	2,6939E-10
DNAJB9	4	2,79571E-10
INTS12	1	2,83159E-10
HIST1H2BD	4	2,85355E-10
TDH	4	2,98791E-10
SLC33A1	4	3,39143E-10
C29H11orf84	3	4,11135E-10
INPP1	1	4,12673E-10
RAP1A	1	5,01104E-10
FDPS	1	5,02972E-10
RWDD1	1	5,0301E-10
LEPROTL1	1	5,12055E-10
PPIL1	4	5,30125E-10
EXOSC1	4	5,48554E-10
RPS4Y1	4	6,01448E-10
CEP57L1	1	6,60645E-10
MPLKIP	4	6,60696E-10

IFT43	1	6,79212E-10
SNHG3	2	7,06082E-10
PQBP1	1	7,46609E-10
XRCC4	1	8,11396E-10
ZNF197	1	8,54631E-10
COX7A1	3	8,63411E-10
ANAPC5	1	8,71282E-10
ALAS1	1	8,91176E-10
ENDOV	1	9,46649E-10
CCDC85A	1	9,55386E-10
FASTKD3	1	1,02304E-09
MOB4	1	1,03737E-09
MYF5	1	1,26352E-09
RAB27B	1	1,30194E-09
ZNF75A	1	1,36245E-09
DGCR8	1	1,40902E-09
ARF4	1	1,43948E-09
LARS	1	1,55185E-09
NFYA	5	1,68622E-09
ZCCHC10	4	1,77536E-09
GART	4	1,89748E-09
MAP4K1	3	2,06387E-09
DESI2	4	2,11214E-09
RPS27	4	2,11679E-09
PDGFRA	4	2,19896E-09
IMP3	4	2,26836E-09
CD52	1	2,29483E-09
ENKD1	1	2,46527E-09
NOL11	4	2,6475E-09
PALB2	1	2,65143E-09
SLC35A3	1	2,67465E-09
RMDN3	1	2,81426E-09
ISG20L2	4	2,92537E-09
FBXL12	4	3,08325E-09
TBCA	1	3,11569E-09
ZNF330	1	3,20866E-09
MTHFD1L	4	3,24844E-09
TAF1D	4	3,81957E-09
CKAP5	1	3,97094E-09
ASZ1	1	4,07974E-09
MACROD1	1	4,21481E-09
POLR1C	4	4,38991E-09
EIF6	4	4,44851E-09
LGALS3	3	4,49756E-09
CBX3	4	4,49799E-09
SPESP1	1	4,81327E-09
ADI1	1	5,0669E-09
DCAF8	1	5,07549E-09
C8H4orf27	1	5,08988E-09
ZBTB9	4	5,12411E-09

ZFAND2B	4	5,32943E-09
TFDP2	1	5,60791E-09
EIF1AD	4	5,62308E-09
TMEM159	3	5,71454E-09
NOP16	4	6,2815E-09
ZNF879	1	6,31823E-09
CMC2	4	6,48633E-09
FAM89A	4	6,76758E-09
TMEM42	3	6,83483E-09
DSCC1	1	7,0952E-09
ATF7IP	4	7,34295E-09
ICA1	1	7,66567E-09
RCBTB1	1	7,86037E-09
LXN	4	8,21383E-09
DEPDC7	1	8,30102E-09
TGS1	4	8,40025E-09
PLA2G4A	1	8,42859E-09
DNAJA4	1	9,18969E-09
UQCC3	4	9,20551E-09
RMDN1	3	9,43449E-09
C3H1orf52	4	9,61933E-09
NME7	1	9,94759E-09
GPALPP1	1	1,00354E-08
GLRX2	2	1,04394E-08
ADHFE1	1	1,04959E-08
BORA	1	1,0629E-08
TCEB2	4	1,07568E-08
TMEM196	1	1,0771E-08
ARHGAP24	1	1,08472E-08
MGAT5	1	1,12336E-08
PUS7	4	1,15242E-08
LHX2	3	1,17527E-08
ZNF266	1	1,18925E-08
PIR	1	1,1968E-08
PRDM14	4	1,19687E-08
TMEM38B	1	1,21973E-08
RPL38	4	1,22444E-08
GLT8D1	1	1,26168E-08
GOLIM4	1	1,27811E-08
WRAP53	4	1,29403E-08
VPS45	1	1,3006E-08
NFU1	1	1,32353E-08
LARP4	4	1,32872E-08
COPB2	1	1,32906E-08
NLRP9	1	1,36909E-08
PEX5L	1	1,41165E-08
MFN2	1	1,46858E-08
LOC530773	3	1,50399E-08
ING1	4	1,53541E-08
ABCA1	1	1,53985E-08

TSPAN14	1	1,54826E-08
BRDT	4	1,5578E-08
ELP4	1	1,5696E-08
CLK1	4	1,59391E-08
GNPDA2	1	1,60016E-08
CHMP7	1	1,6118E-08
APBB1IP	1	1,61675E-08
C2CD5	1	1,64053E-08
ETV6	1	1,66813E-08
HIPK3	4	1,70586E-08
LDHC	1	1,70614E-08
SNAI1	2	1,71212E-08
ZNF133	1	1,92423E-08
BMPR1A	1	1,92831E-08
BMP4	4	1,93364E-08
FAM214B	4	1,95836E-08
CCSER1	1	1,97239E-08
BET1	1	1,98614E-08
TTC32	1	1,98821E-08
IFNGR1	1	2,01183E-08
RPH3AL	1	2,1126E-08
FAM175A	3	2,1706E-08
CLCN3	1	2,24827E-08
VANGL1	3	2,34614E-08
LOC538702	1	2,44114E-08
EFCAB7	1	2,54399E-08
ITGA4	1	2,59645E-08
AGPAT5	4	2,67182E-08
GCDH	1	2,8028E-08
HEXIM1	4	2,83701E-08
SAMD12	3	2,86603E-08
LIMK2	1	2,90128E-08
GNPNAT1	1	2,92014E-08
ACOT9	1	2,97939E-08
CADPS	1	3,01728E-08
CDKN2C	4	3,04157E-08
SMIM8	1	3,12319E-08
MTMR6	1	3,25492E-08
IPMK	4	3,35338E-08
TTC14	4	3,38309E-08
AIFM1	1	3,39759E-08
NANOG	4	3,43914E-08
OSTF1	1	3,4533E-08
GMPPA	1	3,51447E-08
DEF6	1	3,53058E-08
DHX9	4	3,69638E-08
PSTPIP1	1	3,94178E-08
CCNL1	5	3,96771E-08
HTATIP2	1	3,97208E-08
SLC27A1	4	3,97596E-08

SDF2	4	3,97883E-08
STK16	1	4,03451E-08
PRIMPOL	1	4,19026E-08
SDSL	1	4,21746E-08
WDR41	1	4,22941E-08
LIAS	1	4,42739E-08
TRIM23	1	4,45806E-08
RNF144B	1	4,51131E-08
EXOC6	1	4,56677E-08
VPS16	1	4,63143E-08
LPPR1	1	4,63236E-08
DISP3	1	4,65133E-08
NDUFC1	1	4,68752E-08
KLF3	4	4,78944E-08
FBXO38	1	4,90218E-08
MNF1	1	5,04428E-08
PPM1H	1	5,1051E-08
ZNF746	3	5,16426E-08
UACA	1	5,16845E-08
PBRM1	5	5,22857E-08
NAA11	4	5,37618E-08
MAD1L1	1	5,57693E-08
ECE2	4	5,60028E-08
TMEM41B	4	5,72233E-08
AP5S1	1	5,72836E-08
ABCB7	1	5,80744E-08
ZNF408	4	6,27171E-08
CNDP1	1	6,30996E-08
ARL14EPL	3	6,64328E-08
SEPT4	1	6,71677E-08
MED23	1	6,76048E-08
MRPL42	1	6,77048E-08
ZWILCH	1	6,83961E-08
NDUFB2	4	6,87129E-08
C23H6orf62	4	6,92059E-08
RAB7B	3	6,94937E-08
ATXN7L3	4	7,18276E-08
DAG1	3	7,19543E-08
GMPS	1	7,20854E-08
GOPC	1	7,27207E-08
PICK1	1	7,46989E-08
ZSCAN12	1	7,50106E-08
MKLN1	1	7,5481E-08
NFXL1	1	7,81534E-08
MKRN1	4	7,82952E-08
SLC1A1	1	7,92608E-08
OXSM	3	8,05007E-08
AACS	3	8,29841E-08
CXCL3	5	8,31593E-08
MT3	3	8,35287E-08

PHF5A	4	8,82837E-08
LIPE	1	8,86413E-08
MGA	4	8,86413E-08
GNB5	1	9,12954E-08
ANAPC13	1	9,31696E-08
KRR1	4	9,33508E-08
NNT	3	9,34465E-08
HACL1	1	9,87804E-08
BRF2	4	1,00833E-07
CDKAL1	1	1,02613E-07
RAB3IP	1	1,03452E-07
KCTD20	4	1,07114E-07
ME1	1	1,08382E-07
SNHG4	4	1,11707E-07
CCDC86	4	1,11941E-07
PSMA1	4	1,12878E-07
MPHOSPH6	1	1,15002E-07
MCFD2	1	1,21147E-07
KLF5	4	1,22365E-07
GADD45GIP1	1	1,25923E-07
RANBP2	4	1,29326E-07
C4H7orf57	1	1,29667E-07
SLC6A20	3	1,32324E-07
JUP	4	1,34127E-07
MED4	1	1,34988E-07
HUWE1	4	1,35782E-07
G3BP2	5	1,36603E-07
UBASH3B	1	1,42506E-07
MRPL38	1	1,42532E-07
UCK2	4	1,49289E-07
MCPH1	1	1,49296E-07
NQO1	1	1,49438E-07
DPH5	1	1,50008E-07
DAPL1	1	1,50798E-07
HERC3	1	1,5363E-07
SLC25A51	1	1,55429E-07
EGR4	1	1,55772E-07
MTMR1	1	1,56745E-07
ATP9B	1	1,56827E-07
IMPACT	4	1,56827E-07
ASPSCR1	1	1,58179E-07
SNRPB	4	1,58989E-07
SESTD1	1	1,60745E-07
CD3G	1	1,61182E-07
CBX1	1	1,62879E-07
MNS1	1	1,63357E-07
TFCP2	1	1,64009E-07
THEM4	1	1,64507E-07
CEP120	1	1,66286E-07
GJA4	1	1,66949E-07

STK25	1	1,69647E-07
CCDC172	1	1,6992E-07
CCNL2	4	1,71981E-07
RPS9	4	1,72533E-07
VIMP	4	1,73295E-07
NLRP5	1	1,77011E-07
CERS3	3	1,81777E-07
LYPLAL1	1	1,82473E-07
C3H1orf228	1	1,85123E-07
BBS2	1	1,8914E-07
RAB31	1	1,89575E-07
LRRC8C	1	1,93125E-07
FAM216A	1	1,98055E-07
COTL1	4	1,98418E-07
THBS4	1	1,99177E-07
ZMAT5	4	2,00116E-07
PSMD5	1	2,00679E-07
LRRC1	1	2,1202E-07
PTTG1IP	1	2,18208E-07
MKRN2	1	2,18309E-07
DPYSL2	4	2,18671E-07
ANKRD16	1	2,19575E-07
CRLS1	1	2,20763E-07
IPO7	4	2,20763E-07
FRA10AC1	1	2,21084E-07
VDAC3	3	2,27973E-07
NDEL1	1	2,30481E-07
MAPK1	1	2,30587E-07
GPR158	1	2,3126E-07
SCO1	4	2,31621E-07
SMAD1	1	2,33137E-07
EWSR1	4	2,33579E-07
EEF2KMT	3	2,33919E-07
LRRC40	4	2,34432E-07
PGM2L1	1	2,35481E-07
PRKCH	1	2,35718E-07
PRICKLE1	1	2,38277E-07
PRDM6	1	2,44217E-07
GRHPR	1	2,44862E-07
TMEM223	4	2,47354E-07
CUTC	1	2,47526E-07
KIAA0922	1	2,50076E-07
PITX2	4	2,52829E-07
TEX261	3	2,56322E-07
DIAPH2	1	2,58336E-07
WDR34	1	2,66635E-07
RPL10A	4	2,66934E-07
STK39	1	2,73831E-07
PRPS2	1	2,76999E-07
DNA2	1	2,83878E-07

KLF10	4	2,92507E-07
PRDX5	1	2,92876E-07
MTG1	1	2,98026E-07
MYO6	1	3,02371E-07
IQCC	1	3,0306E-07
NKAPL	4	3,044E-07
VAT1	1	3,05004E-07
DNMBP	1	3,07444E-07
LMO1	1	3,35397E-07
ZNF296	2	3,40946E-07
CMTM8	1	3,45163E-07
SLC4A4	1	3,45667E-07
HPS3	1	3,49231E-07
PPM1K	4	3,78709E-07
NUP37	1	3,81705E-07
ZNF345	1	3,82427E-07
COLEC12	1	3,85007E-07
RPS29	4	3,94228E-07
CDK1	4	3,94532E-07
WDR55	4	3,94541E-07
RALYL	1	3,96782E-07
METTL12	4	3,99785E-07
MRPL43	3	4,00801E-07
TNFSF12	3	4,02026E-07
PAXBP1	4	4,11458E-07
MRPL14	1	4,141E-07
GABPB1	1	4,17339E-07
CDK20	2	4,17835E-07
PHLDB1	1	4,2678E-07
PSMG1	1	4,30733E-07
MTMR3	1	4,315E-07
VWA9	1	4,33378E-07
NIPA2	4	4,39402E-07
MAP4K5	1	4,47833E-07
CNN2	3	4,60029E-07
SCP2	1	4,65729E-07
LSS	5	4,71869E-07
PARP1	1	4,75091E-07
HTRA2	1	4,79654E-07
PTGR2	1	4,96511E-07
PTGFR	4	5,0203E-07
OTUD7B	1	5,04926E-07
TMEM150A	3	5,06519E-07
TTK	1	5,07875E-07
CAD	4	5,08804E-07
TXNIP	1	5,1631E-07
NCBP2	4	5,17707E-07
LHFPL4	1	5,17797E-07
SYNRG	1	5,18043E-07
MCM5	1	5,23965E-07

PLEKHO1	1	5,2718E-07
JMJD6	4	5,27702E-07
MTFMT	1	5,28842E-07
CNKSR1	3	5,31184E-07
TMEM50B	1	5,64758E-07
TMEM219	3	5,6551E-07
ZAR1	1	5,68072E-07
LOC100848703	1	5,71233E-07
VWF	1	5,7866E-07
BMPR1B	1	5,9483E-07
ALG14	3	6,00511E-07
TRAF3IP1	1	6,06093E-07
BCKDK	1	6,12594E-07
RAPGEF4	1	6,25979E-07
ATIC	1	6,32902E-07
ZNF395	3	6,64882E-07
METTL14	1	6,682E-07
RIC3	1	6,82601E-07
CLHC1	1	6,83469E-07
BRI3	3	6,93051E-07
RIIAD1	3	6,93933E-07
SUV39H2	1	7,02516E-07
TBC1D19	3	7,06088E-07
CEP44	1	7,13511E-07
BFAR	1	7,28502E-07
PCDH8	4	7,31367E-07
SNX2	1	7,46102E-07
UBASH3A	1	7,50187E-07
HVCN1	1	7,60446E-07
CEP41	3	7,80115E-07
MGC148714	3	7,81972E-07
MZB1	1	8,3339E-07
SYNM	1	8,34288E-07
EBPL	1	8,44408E-07
FAM213A	1	8,46266E-07
LCP1	3	8,47368E-07
KCNJ3	1	8,63353E-07
TMIGD1	1	8,70335E-07
ORC4	1	8,71025E-07
VPS25	1	8,74117E-07
RPL6	4	8,87859E-07
SCML2	1	8,88442E-07
MID1	1	9,07332E-07
GALK2	1	9,11876E-07
SPATA22	1	9,14146E-07
CRIM1	1	9,59028E-07
PRDX3	1	9,73771E-07
TMEM266	1	9,96732E-07
RCOR2	3	1,00105E-06
C5H12orf66	1	1,00882E-06

METTL6	1	1,08636E-06
TMEM242	1	1,09852E-06
NRBP1	5	1,1007E-06
CSRNP2	4	1,1011E-06
BUB1B	1	1,10361E-06
FKBP3	4	1,11614E-06
POLI	1	1,1225E-06
DNAJC15	3	1,16074E-06
CYSTM1	3	1,17542E-06
CROT	5	1,17551E-06
TMEM50A	3	1,19121E-06
KCTD3	1	1,21883E-06
RNASEH2C	4	1,21895E-06
ETS1	1	1,22399E-06
COQ2	1	1,23982E-06
B3GALNT2	1	1,24069E-06
ARPC4	1	1,24126E-06
GLS2	1	1,24845E-06
GABPA	4	1,25357E-06
PLEKHA8	1	1,26671E-06
SOCS2	5	1,27634E-06
ABCG5	1	1,27779E-06
TBCEL	1	1,27792E-06
ZMYM1	4	1,30548E-06
EML5	1	1,30915E-06
ATXN7L1	1	1,32038E-06
LYRM7	1	1,33738E-06
UROS	1	1,348E-06
TDRD7	1	1,36378E-06
NT5E	1	1,42406E-06
ZNF548	1	1,43437E-06
CNPPD1	1	1,44183E-06
RPL37	4	1,46989E-06
MOV10	1	1,47934E-06
PLEKHM3	1	1,52716E-06
SORBS1	1	1,53572E-06
NSMCE1	1	1,54571E-06
SKAP2	1	1,54988E-06
BBS5	1	1,56712E-06
ACSL3	1	1,56976E-06
TJP2	1	1,57783E-06
FTH1	3	1,62537E-06
NUDT22	1	1,64212E-06
EPC1	3	1,64954E-06
QTRT2	1	1,65619E-06
BCL2L12	4	1,66841E-06
CBR1	1	1,67579E-06
PON2	1	1,71989E-06
IMPDH1	4	1,72543E-06
KTI12	4	1,74985E-06

IRF6	1	1,78807E-06
DPPA2	4	1,81746E-06
FBN1	3	1,83043E-06
AKR1A1	1	1,84347E-06
DLD	4	1,84585E-06
PPP4R3A	4	1,8684E-06
PLXDC2	1	1,87219E-06
FAM21A	5	1,87242E-06
CHMP2B	1	1,88247E-06
ANO5	1	1,88358E-06
LRRC6	1	1,90753E-06
DENND2C	4	1,98051E-06
ARHGAP26	1	1,99784E-06
FRS3	1	2,02602E-06
IL1R1	1	2,02944E-06
GPR1	1	2,05102E-06
C7H5orf45	1	2,08935E-06
SGCE	1	2,08935E-06
SRSF6	2	2,10295E-06
MSMO1	1	2,10667E-06
SERP2	3	2,10823E-06
RTN2	4	2,12321E-06
CRYZL1	1	2,1279E-06
RPE	4	2,13889E-06
EHMT1	1	2,16692E-06
TFAM	4	2,18344E-06
ACSL5	5	2,18653E-06
MICU1	4	2,19154E-06
ZNF438	1	2,19212E-06
NAB1	1	2,22477E-06
ZNF35	1	2,22748E-06
SEC22B	1	2,24992E-06
HAGH	1	2,2993E-06
UBA3	1	2,30854E-06
AP1S1	1	2,32773E-06
TMEM167B	1	2,34635E-06
LOC789175	4	2,35248E-06
MANF	4	2,35281E-06
PARK2	1	2,36339E-06
MBIP	4	2,40168E-06
CDK5RAP1	1	2,42124E-06
ZNF713	1	2,43167E-06
UQCRFS1	4	2,45005E-06
PRSS23	3	2,45429E-06
COX20	1	2,50766E-06
AMN1	1	2,50846E-06
LZTFL1	1	2,51824E-06
EPS15L1	1	2,54296E-06
HILPDA	1	2,55432E-06
SLC38A8	3	2,57296E-06

GSTA2	4	2,57331E-06
DNAJC11	3	2,61057E-06
TMEM186	4	2,62176E-06
ODF4	1	2,63147E-06
TUBGCP4	1	2,63358E-06
DLST	1	2,64865E-06
NOL6	4	2,67074E-06
CXADR	4	2,69602E-06
RBMX	4	2,69724E-06
FZD3	1	2,70677E-06
TOB1	4	2,71252E-06
PTPRF	1	2,73373E-06
ORC6	1	2,74575E-06
CYCT	4	2,76467E-06
PSMD6	1	2,7934E-06
TM9SF1	1	2,79637E-06
SLC25A31	3	2,81898E-06
SLC24A2	1	2,83562E-06
ALKBH3	1	2,8471E-06
COQ8A	3	2,91578E-06
ZNF22	1	2,92361E-06
EFTUD2	1	2,94302E-06
GJD2	1	3,03497E-06
TMEM168	1	3,03497E-06
SH2B1	1	3,05681E-06
YPEL3	3	3,09345E-06
TMEM101	1	3,10296E-06
ATG4D	4	3,10811E-06
TMEM161A	1	3,1191E-06
SUPV3L1	1	3,17727E-06
DNAJB4	1	3,20085E-06
APOO	1	3,2178E-06
ROBO1	1	3,23037E-06
KLHDC2	1	3,24824E-06
PREP	1	3,29614E-06
SOX30	1	3,3045E-06
CCT4	4	3,38417E-06
EED	4	3,4271E-06
SOCS7	3	3,44729E-06
MPV17L2	1	3,45434E-06
F11R	4	3,49833E-06
ALDH1A1	1	3,51421E-06
MTM1	1	3,5155E-06
TXNDC5	1	3,55539E-06
PRPF3	1	3,58658E-06
MEDAG	4	3,66654E-06
CAB39	1	3,68164E-06
SNX19	3	3,69942E-06
MESDC1	4	3,79639E-06
RTN4	2	3,79775E-06

RNF44	4	3,80896E-06
MYL12B	1	3,81013E-06
UTP18	5	3,85036E-06
ZBTB6	1	3,86509E-06
C15H11orf74	1	3,90326E-06
DPH2	1	3,91133E-06
SDF2L1	1	3,91263E-06
PSMB7	1	3,91926E-06
PMPCB	1	3,92406E-06
LACC1	1	3,93926E-06
FAM72A	1	3,94538E-06
TSC22D1	4	3,94864E-06
FAM76B	1	3,99972E-06
UBE2L6	1	4,02458E-06
SIPA1L2	1	4,13407E-06
CLCC1	3	4,15782E-06
HDGF	3	4,17356E-06
C20H5orf47	1	4,26233E-06
FAM45A	4	4,3261E-06
TMEM39A	3	4,36023E-06
ZNF692	1	4,42323E-06
CASP2	1	4,45322E-06
NR2F2	4	4,50962E-06
PPAT	4	4,5709E-06
UFL1	1	4,61169E-06
SPATA2L	3	4,61693E-06
SCCPDH	3	4,68562E-06
LSM8	4	4,70706E-06
TTC4	1	4,7889E-06
IFT88	1	4,81228E-06
PLEKHJ1	4	4,92065E-06
HARS	1	4,99265E-06
JUN	1	5,00038E-06
COPRS	1	5,02396E-06
ZNF184	1	5,08874E-06
PDPN	1	5,10833E-06
ELMOD2	1	5,18102E-06
SNAPC5	4	5,19944E-06
RTKN2	1	5,20383E-06
TMX4	1	5,21588E-06
NDUFS4	1	5,2671E-06
NPC1	5	5,35579E-06
SRI	1	5,43197E-06
SLC16A13	1	5,51399E-06
MAP4	3	5,53716E-06
LUZP2	1	5,54525E-06
PEX3	1	5,58656E-06
RUNDC1	1	5,60636E-06
SIRT1	4	5,61216E-06
RAB3GAP1	5	5,61261E-06

C14H8orf59	4	5,63431E-06
QARS	4	5,64074E-06
RDH12	1	5,6642E-06
RPL13A	4	5,67315E-06
ZNRF1	4	5,70061E-06
CDH7	1	5,72375E-06
SASS6	1	5,73426E-06
DCTN3	1	5,7818E-06
TMX3	1	5,79351E-06
NSMCE2	1	5,8117E-06
DDRGK1	1	5,88662E-06
PMS1	1	6,01496E-06
C11H2orf49	3	6,19342E-06
EIF2B3	3	6,1981E-06
IDE	1	6,29635E-06
TMEM100	1	6,36518E-06
NRBF2	4	6,38535E-06
SEMA4A	1	6,45739E-06
PCYT1B	1	6,51251E-06
SYT11	4	6,51582E-06
NOL8	4	6,59123E-06
TRAPPC13	4	6,66253E-06
ANKRD50	4	6,73795E-06
ZAK	1	6,75989E-06
TGFBRAP1	1	6,82721E-06
PRKG1	3	6,89694E-06
NCAPH	1	7,03305E-06
NECAP1	4	7,06405E-06
STXBP5	1	7,07236E-06
TSTD3	1	7,13407E-06
MFSD13A	1	7,26535E-06
TMEM45A	1	7,28436E-06
ITGA2B	4	7,34353E-06
NKX2-2	1	7,34353E-06
RDH14	1	7,40151E-06
GPATCH1	3	7,68982E-06
PXMP4	1	7,73642E-06
TAF11	4	7,79406E-06
NFKBIB	4	7,86984E-06
RNF219	1	7,96974E-06
CDCA7	1	7,97139E-06
MRPS35	1	8,03397E-06
SPATA24	1	8,06356E-06
LTBR	1	8,06982E-06
TIMELESS	1	8,13859E-06
WDFY2	3	8,25045E-06
ALG8	1	8,32588E-06
ZCCHC7	1	8,34476E-06
MCC	1	8,366E-06
GPATCH2	1	8,37165E-06

TIFA	1	8,38114E-06
UNC50	1	8,41033E-06
SPON1	1	8,47038E-06
CCDC91	1	8,48912E-06
ATP6V0C	1	8,58808E-06
FBXO10	3	8,6654E-06
CRYM	4	8,6823E-06
VPS26A	1	8,69839E-06
MRAP2	1	8,75973E-06
COMMD2	1	8,76027E-06
TIMM17B	1	8,82549E-06
ARHGEF12	1	8,9861E-06
MAP2K1	1	9,00534E-06
PLCL2	3	9,01535E-06
MYC	4	9,07149E-06
NSG1	1	9,08039E-06
COL4A3BP	5	9,2755E-06
DYRK3	5	9,38731E-06
AKAP6	1	9,55267E-06
DICER1	1	9,61043E-06
PCDH10	4	9,66191E-06
TMEM86A	1	9,77883E-06
RPAP1	1	9,78134E-06
ILDR1	3	9,79006E-06
RARG	1	9,83356E-06
LMX1A	1	9,92271E-06
NADK2	1	9,97423E-06
LMOD3	1	1,00436E-05
IFT52	1	1,0077E-05
MED9	4	1,00804E-05
JMJD1C	1	1,04827E-05
ADAMTS1	2	1,0565E-05
ATP5G2	1	1,06575E-05
MIER3	1	1,06926E-05
PUS7L	1	1,0705E-05
ZW10	1	1,07317E-05
BLZF1	1	1,0798E-05
KIAA0754	1	1,08573E-05
ACAD11	1	1,08614E-05
ARHGEF39	1	1,08927E-05
TRIM59	3	1,09276E-05
FEN1	4	1,09481E-05
NFKBIE	1	1,09952E-05
DUSP5	1	1,10414E-05
ATP10D	1	1,1058E-05
DSTYK	1	1,11329E-05
PRKCI	1	1,11409E-05
P4HA3	1	1,14299E-05
DNAL4	3	1,14546E-05
SEC16B	3	1,14954E-05

BSDC1	1	1,15298E-05
ADAMTS9	1	1,16223E-05
SLC40A1	1	1,16306E-05
MRPL33	1	1,18592E-05
OSBPL11	1	1,19738E-05
GLOD4	1	1,2015E-05
NUPL2	4	1,20451E-05
RNASEH1	4	1,22028E-05
C1H21orf33	1	1,22155E-05
GAB1	1	1,22716E-05
THUMPD3	1	1,2357E-05
DCTN1	1	1,24104E-05
PFKFB3	1	1,24168E-05
TBC1D2	4	1,26374E-05
GADD45B	1	1,2878E-05
SORCS3	1	1,28791E-05
CHD8	4	1,29631E-05
PPP3R1	1	1,31934E-05
OVOL1	1	1,32976E-05
LOC100847759	4	1,34232E-05
TMEM184B	1	1,34451E-05
ENO1	1	1,34486E-05
MECP2	3	1,34486E-05
WDR61	1	1,3523E-05
MRPL3	1	1,36781E-05
MTIF2	1	1,3816E-05
ATP5A1	1	1,40596E-05
MDGA1	1	1,40927E-05
STAP2	1	1,42219E-05
TAF13	4	1,42329E-05
ITGB4	1	1,44699E-05
PPP1R21	1	1,4487E-05
RBAK	4	1,45591E-05
UCHL5	1	1,46931E-05
STX17	1	1,47249E-05
CCDC50	3	1,47413E-05
RECQL	1	1,49872E-05
SOD2	1	1,53397E-05
PGAM5	3	1,53576E-05
FANCM	1	1,53623E-05
NDUFAF7	1	1,54785E-05
FAM76A	1	1,54969E-05
SLC9A9	1	1,5578E-05
ZNF135	1	1,56296E-05
ETFDH	1	1,56638E-05
PRDX6	3	1,56801E-05
WDPCP	1	1,58926E-05
MND1	1	1,59629E-05
RPAIN	1	1,59726E-05
C10H14orf1	1	1,60382E-05

MAPK1IP1L	4	1,60611E-05
CLIP1	6	1,62183E-05
RAD52	3	1,63089E-05
TMA16	1	1,66118E-05
SLC17A5	1	1,66737E-05
ZNF419	1	1,6825E-05
PIH1D2	1	1,69865E-05
ZMAT3	1	1,71195E-05
UIMC1	1	1,71248E-05
RNF130	1	1,72359E-05
PCCB	1	1,7262E-05
NDUFA12	3	1,7304E-05
MTMR14	4	1,73783E-05
SLC25A15	1	1,7383E-05
ANKRD39	3	1,75236E-05
ARHGAP1	4	1,75408E-05
CISD2	1	1,75963E-05
LOC613444	4	1,77128E-05
FBXO11	5	1,773E-05
DPP6	1	1,77348E-05
ST5	1	1,7836E-05
IGFBP3	1	1,78818E-05
ARFIP2	1	1,80335E-05
OIP5	1	1,8098E-05
PATJ	1	1,81588E-05
ARNTL	3	1,82145E-05
GFPT2	1	1,82807E-05
MRPS15	3	1,85354E-05
C4H7orf25	3	1,85897E-05
SNX4	1	1,86337E-05
BAMBI	4	1,86485E-05
NIPAL1	4	1,86921E-05
KATNBL1	3	1,86925E-05
CAPN14	1	1,9019E-05
CPB2	1	1,91098E-05
SCG3	1	1,91971E-05
IPP	1	1,94646E-05
NDUFB3	4	1,94994E-05
DDA1	1	1,96373E-05
FLAD1	1	1,9839E-05
DTX2	1	1,99508E-05
COQ7	1	2,01552E-05
ST7L	1	2,03921E-05
LUC7L	4	2,05431E-05
ATP23	4	2,07907E-05
GHDC	3	2,11914E-05
ODZ3	1	2,1772E-05
HAUS4	1	2,23576E-05
TRIM52	1	2,24358E-05
PIH1D1	4	2,28904E-05

ARL4D	2	2,30246E-05
MAGOHB	1	2,30367E-05
RAB6B	1	2,34365E-05
TTF2	1	2,34689E-05
SSSCA1	4	2,34999E-05
DTX1	1	2,36564E-05
SNX11	1	2,37992E-05
HACE1	1	2,38917E-05
HKDC1	1	2,39252E-05
ZBTB40	1	2,39857E-05
YTHDF1	1	2,41698E-05
DIRC2	1	2,43405E-05
GTF2I	1	2,48933E-05
CPSF6	4	2,51672E-05
SDHAF3	1	2,51786E-05
TMEM110	1	2,5349E-05
TCIRG1	1	2,53495E-05
SMAD3	1	2,5524E-05
CXXC1	1	2,57434E-05
ELK3	1	2,59572E-05
OMA1	1	2,60376E-05
PDSS2	1	2,6088E-05
USP3	4	2,61972E-05
CDK14	1	2,68195E-05
METTL3	1	2,71201E-05
UBXN1	1	2,71549E-05
RNF181	1	2,7684E-05
PEX5	1	2,80148E-05
FBXO3	1	2,81177E-05
WDR74	4	2,82611E-05
TTLL4	1	2,86623E-05
CAT	1	2,86709E-05
TSPYL4	1	2,89684E-05
WDR48	1	2,91989E-05
IP6K1	1	2,9309E-05
PRRG2	4	2,95881E-05
ZNF397	1	2,9772E-05
EXOSC9	1	3,02059E-05
LSM12	3	3,0481E-05
CPNE1	3	3,04999E-05
EIF3D	4	3,05051E-05
WIF1	1	3,06313E-05
PJA2	1	3,07504E-05
MTHFD2	3	3,12791E-05
SLC39A12	3	3,15183E-05
SSX2IP	5	3,19114E-05
C18H16orf87	4	3,19196E-05
C3H1orf123	3	3,22518E-05
UBXN4	5	3,22534E-05
SHISA2	1	3,22696E-05

AP1AR	1	3,24269E-05
FAM89B	4	3,2465E-05
AXIN1	1	3,25789E-05
ECI2	1	3,30119E-05
MFSD1	1	3,3027E-05
RNF167	4	3,31544E-05
MGEA5	4	3,36687E-05
ZNF326	4	3,38222E-05
TRIM25	1	3,38782E-05
ZBED8	1	3,3913E-05
TNFAIP8L1	2	3,40834E-05
KCNN4	3	3,41182E-05
MED31	4	3,43803E-05
APBA3	1	3,44894E-05
INSIG1	3	3,49925E-05
SLC20A1	1	3,50108E-05
PYM1	1	3,51026E-05
AHSA2	1	3,51027E-05
FXN	1	3,53063E-05
AARSD1	1	3,54248E-05
GFI1	1	3,56239E-05
MGP	4	3,57649E-05
RHBDL2	1	3,57649E-05
OPA1	1	3,58235E-05
METTL22	3	3,62608E-05
SPINK5	1	3,63403E-05
ETV1	1	3,67809E-05
KLHDC1	1	3,68188E-05
S100B	1	3,73137E-05
TRPS1	1	3,77641E-05
GUCY2C	4	3,83616E-05
MRPL19	1	3,92947E-05
NKAIN2	1	3,96311E-05
MRVI1	1	3,96693E-05
NEGR1	1	3,98281E-05
FHIT	3	3,98401E-05
CREB5	1	4,02096E-05
GTF2H3	1	4,05024E-05
ATXN3	1	4,05458E-05
RGS3	1	4,0651E-05
SLC22A5	3	4,06733E-05
CMTM7	3	4,07465E-05
MYOF	1	4,08808E-05
CHEK1	5	4,1628E-05
GRAP2	1	4,19055E-05
AMT	4	4,19643E-05
ACAT1	1	4,20804E-05
PDIA5	3	4,22524E-05
HIKESHI	1	4,23337E-05
TXN2	4	4,23348E-05

DHX35	1	4,25184E-05
SHOX2	3	4,33918E-05
MFSD14A	1	4,35623E-05
IL1R2	1	4,35753E-05
EDF1	3	4,36043E-05
EFNB1	1	4,36964E-05
CAPN7	1	4,4098E-05
PAFAH1B2	3	4,42322E-05
NBR1	5	4,45557E-05
NCOA4	1	4,4556E-05
MYH2	1	4,47136E-05
LOC615809	4	4,47421E-05
ASAH1	1	4,50313E-05
ZNF34	1	4,53759E-05
PATZ1	3	4,55005E-05
MYH8	1	4,64381E-05
GSDMC	1	4,64973E-05
TIMMDC1	4	4,67141E-05
KIF15	1	4,67368E-05
LOC534742	3	4,67483E-05
KCNK1	1	4,6785E-05
WTIP	1	4,7181E-05
CCNH	3	4,74782E-05
CPNE8	4	4,76346E-05
MDM4	1	4,76521E-05
GNA14	1	4,80132E-05
RARS2	1	4,80132E-05
ETFA	1	4,82717E-05
PIGP	1	4,83491E-05
SLC10A7	1	4,84919E-05
POLR2F	4	4,88082E-05
ZFAND3	1	4,88964E-05
USP38	4	4,89607E-05
CHAC1	3	4,91663E-05
BMS1	4	4,92266E-05
LMO3	1	4,92386E-05
CCNB1IP1	1	4,96539E-05
SHROOM3	1	4,96843E-05
ANAPC11	3	5,02037E-05
NOL4	1	5,0853E-05
MTERF4	1	5,08575E-05
NIPSNAP3A	4	5,10771E-05
GNA11	3	5,11095E-05
FANCI	1	5,11539E-05
HACD2	1	5,15094E-05
FBXO21	3	5,16816E-05
RCSD1	4	5,22947E-05
TBC1D14	1	5,25303E-05
HACD3	1	5,27998E-05
GRK3	1	5,28331E-05

TAOK3	3	5,30428E-05
TUFT1	4	5,33506E-05
RMI2	1	5,34715E-05
PSME4	4	5,4057E-05
ACTL6A	4	5,41157E-05
RCN2	1	5,4174E-05
ACTR6	1	5,42763E-05
MACROD2	1	5,4389E-05
PEX11A	1	5,52055E-05
MTHFSD	1	5,57685E-05
RTN4IP1	1	5,59546E-05
NCF2	1	5,59672E-05
UBE3A	5	5,60771E-05
GNAI1	1	5,61839E-05
DARS	1	5,63164E-05
CCDC93	1	5,67131E-05
LRIG2	1	5,67293E-05
SMDT1	4	5,67543E-05
MRS2	4	5,67923E-05
SLC25A14	1	5,72771E-05
DGKB	3	5,72975E-05
SPATA5L1	4	5,7398E-05
DSP	1	5,83171E-05
BTBD1	4	5,84223E-05
C14H8orf76	3	5,84478E-05
CCDC65	1	5,87803E-05
CHD6	1	5,89582E-05
VTI1B	1	5,93493E-05
LRRIQ4	1	5,95457E-05
TMUB2	4	6,03038E-05
MRPL1	4	6,09225E-05
LRSAM1	1	6,1556E-05
XPA	3	6,1556E-05
SNTA1	1	6,15577E-05
GPRC5D	1	6,20985E-05
HORMAD1	4	6,2119E-05
SHFM1	4	6,2119E-05
GPR39	1	6,25365E-05
KATNAL1	1	6,2556E-05
CARD19	4	6,25606E-05
XRN1	1	6,26179E-05
HIBADH	1	6,28453E-05
AK6	4	6,34037E-05
MALSU1	4	6,34037E-05
RBP4	1	6,34282E-05
LCT	1	6,34817E-05
SPR	1	6,3973E-05
MTO1	1	6,4051E-05
DDX4	1	6,41119E-05
DPCD	1	6,43957E-05

NR4A1	1	6,48777E-05
CHGA	1	6,52986E-05
UBE2S	2	6,53191E-05
SYNJ2BP	1	6,54964E-05
FERMT1	1	6,57465E-05
BSP5	1	6,58392E-05
BTBD9	3	6,60431E-05
GCC1	1	6,81535E-05
ANXA11	1	6,86996E-05
RAF1	1	6,9112E-05
BPHL	1	7,00806E-05
NUAK2	1	7,02791E-05
ATOX1	1	7,0604E-05
RSPO3	1	7,13815E-05
TCHP	1	7,14718E-05
LAS1L	5	7,29709E-05
NDUFB6	1	7,30707E-05
AIMP1	4	7,35503E-05
EFR3A	1	7,36856E-05
BCL10	1	7,40049E-05
NUBPL	1	7,40841E-05
TOB2	4	7,41736E-05
PTGS2	1	7,42133E-05
TMEM230	1	7,50639E-05
CD63	1	7,54435E-05
SLC38A9	1	7,5725E-05
CD247	1	7,61744E-05
EPB41L4A	1	7,64138E-05
SERTAD1	4	7,65315E-05
GALNT1	1	7,70498E-05
ZIM2	1	7,73761E-05
PAN2	1	7,75566E-05
ANKRD46	1	7,77539E-05
CYBRD1	3	7,7805E-05
DDHD1	1	7,84788E-05
RBM46	1	7,88053E-05
DNAJC12	4	7,90329E-05
ACOT7	1	7,9201E-05
WDR83	4	7,9652E-05
LRRC8E	3	8,04857E-05
MARCH11	1	8,09861E-05
POLL	1	8,10231E-05
GPD1L	4	8,19719E-05
OSBPL3	1	8,28703E-05
PLEKHA3	1	8,35395E-05
ADCY6	1	8,35554E-05
C3H1orf50	1	8,35554E-05
PLEKHA1	1	8,51409E-05
FBXO15	1	8,67911E-05
DENND1A	1	8,75373E-05

LNX1	1	8,77264E-05
SPRY1	1	8,79353E-05
POLR2D	4	8,8046E-05
CFAP97	3	8,9237E-05
GCNT1	1	8,96606E-05
CCAR1	4	9,0549E-05
ADRM1	1	9,0841E-05
KIZ	1	9,24523E-05
HRASLS	1	9,26339E-05
TCEA1	1	9,28069E-05
TIMM9	3	9,28627E-05
RGS10	3	9,29226E-05
PLPP2	1	9,32008E-05
PPP3CC	1	9,34404E-05
SNAPC3	4	9,35019E-05
SLC35B2	1	9,36344E-05
TBCD	1	9,38685E-05
FAAP24	1	9,38953E-05
PHACTR3	3	9,39886E-05
PEX14	1	9,46228E-05
ABCD4	1	9,49744E-05
CXHXorf36	1	9,54592E-05
MOB1A	4	9,5845E-05
BEND4	1	9,62964E-05
CORO6	3	9,65013E-05
ATL3	1	9,71092E-05
TFDP1	1	9,73957E-05
SLC23A2	1	9,75596E-05
TEX9	1	9,79474E-05
NEIL1	3	9,82469E-05
ELK4	1	9,87328E-05
CIRBP	1	9,87641E-05
CTDSPL2	1	9,87641E-05
SNRNP40	3	9,88412E-05
SPINK2	3	9,88732E-05
THOP1	3	9,90959E-05
DDB2	1	9,93511E-05
NPHS2	1	9,95011E-05
GRAMD1A	1	0,000100031
SURF4	3	0,00010076
ATG3	4	0,000100795
PPP2R2B	1	0,000101066
CDIP1	3	0,000101271
DNTTIP1	1	0,000101499
EXOSC8	3	0,000102142
FBXO48	1	0,000102267
NCAPG2	1	0,000102342
C15H11orf49	1	0,00010339
TERF2	1	0,000103474
APLF	5	0,000103495

WFIKKN2	1	0,000103538
ETFRF1	1	0,000103804
TMCC1	3	0,000103962
WDTC1	1	0,000104196
PSMD10	1	0,000105365
GSTA4	1	0,000105475
ZDHHC17	4	0,00010583
HNF1B	3	0,000106689
SLC11A2	1	0,000107772
MSRB2	1	0,000107991
DSC3	4	0,000108241
ICE2	1	0,000108531
PLEKHG3	1	0,000109019
RWDD3	1	0,000109474
MKKS	1	0,00010978
TLE3	1	0,000109969
DPP8	1	0,000110936
SPAST	1	0,000112074
GFM2	1	0,000112911
GFER	3	0,000113018
DDIT4	3	0,000113652
MRE11A	1	0,000114259
CLDN10	1	0,000114278
ZNF277	1	0,000115275
G2E3	4	0,000115582
TIMM10	4	0,000115749
NDUFAF6	3	0,000116252
POLD1	1	0,000116431
IMPA2	1	0,000116597
LAMTOR2	3	0,000116597
CHD1	4	0,000117408
TXNL4A	3	0,000117649
TIPRL	1	0,000118591
RNF115	1	0,000118726
SFT2D1	3	0,000119965
CENPH	1	0,000120045
NELL2	4	0,000120282
ELL	3	0,000120763
SNX15	1	0,000121012
SMARCA1	1	0,000122549
LRRTM2	1	0,000123008
RPA1	3	0,000124099
UTP4	4	0,000124195
XKRX	1	0,00012435
HSPA1A	1	0,000124573
XXYLT1	1	0,000124629
EMC10	4	0,00012487
CRY1	1	0,000124906
FAM96B	1	0,000124939
CAPRIN1	4	0,000126645

NPR2	1	0,000126717
SPIRE2	1	0,000126853
CHM	5	0,000127393
PACSIN2	3	0,000128399
C25H16orf91	4	0,000129527
NFKB2	3	0,00012987
ACYP1	3	0,00013031
CAMKK2	3	0,000130594
PNPO	5	0,000131489
TRAPPC9	1	0,000132492
RRP8	1	0,000133203
METTL13	1	0,00013552
SLC18A2	1	0,000136892
SCMH1	3	0,000139035
SOX5	1	0,000140433
KIFAP3	1	0,000141193
TRIM3	3	0,000142091
TOMM5	4	0,000142137
AQR	1	0,000142985
TIMM22	4	0,000143397
CMYA5	1	0,000143633
ACAP2	1	0,000143895
LARP4B	1	0,000143921
VPS11	1	0,000144701
CTCFL	1	0,000144846
TMEFF1	1	0,000145908
PGM2	4	0,000146537
RNF170	1	0,00014858
ZFYVE26	1	0,000148912
HEATR4	1	0,000149954
IFIT3	1	0,000150066
SH3GLB2	3	0,000150093
SLC9A3R1	3	0,00015021
TRIM21	3	0,000151803
PIP5K1C	3	0,000153109
GIGYF1	1	0,0001539
UBE2M	1	0,000154735
ZNF33B	1	0,000154841
MCU	1	0,000155465
PSPH	1	0,000156003
SLC25A3	4	0,000159395
SMN2	1	0,000159652
NFKB1	1	0,000162282
UEVLD	1	0,000162282
PIN1	4	0,000163103
HDHD2	3	0,000163603
NFKBIA	1	0,000164227
CASP6	1	0,000164597
TIMD4	1	0,000165293
MBNL3	1	0,000165321

MSANTD3	1	0,000165634
SLC39A1	1	0,000165964
PLPP1	1	0,00016648
ERCC4	1	0,00016758
GABRG1	1	0,000167611
CFAP43	1	0,0001681
AAMP	4	0,000168748
YIPF6	1	0,000170307
STXBP6	1	0,000170597
PEX7	1	0,000170971
PTPRK	3	0,000170971
DOCK10	1	0,000171594
ADGRG1	1	0,000171696
FAM234A	1	0,000172714
CLVS2	1	0,000174809
RNASEH2A	1	0,000175113
TSPAN13	1	0,000176046
YPEL4	3	0,000176265
CLGN	1	0,000176975
AIDA	1	0,000179424
XRCC3	1	0,000179437
FGR	1	0,000181451
TSEN15	1	0,000182154
NRIP3	3	0,000183325
ZNF3	1	0,000183339
CDK5	1	0,000185423
BRWD1	1	0,000185801
CDKN1A	1	0,000186007
CCNE1	2	0,000186054
ELMOD1	1	0,000186825
RCHY1	1	0,000187107
LIN28A	1	0,000187658
SLC35A1	1	0,00018825
PRDM1	1	0,000189574
MOB1B	1	0,000190102
REL	1	0,000191623
PPTC7	1	0,000191944
ERCC5	1	0,000192413
FOSL1	1	0,000193373
MADD	1	0,00019387
ARHGEF4	1	0,000195015
PDIA3	2	0,000195352
HOXB7	1	0,000195442
BACE2	1	0,000195524
RTEL1	1	0,000195723
PCDHGC3	1	0,000196135
GTF2H1	4	0,000196848
NOP58	4	0,000197242
PRKAA2	1	0,000197652
RRAGD	1	0,000199719

PCP4	1	0,000202346
GAD1	1	0,000203553
LRRC59	1	0,000204438
MED18	1	0,000204722
UBA5	1	0,000205172
MRPL22	4	0,000206049
CDKL1	1	0,0002061
SLC35B1	3	0,00020741
CHD9	1	0,000209251
ZNF512	1	0,00020978
POLR2M	1	0,00020998
MASTL	3	0,000209986
PIM2	1	0,000210201
RUNDC3B	1	0,00021025
UQCR10	4	0,00021025
CPSF4	1	0,000210817
RIPK1	1	0,000211149
ATG12	4	0,000211583
RRP1	1	0,000213644
NEK9	1	0,00021425
PFDN4	1	0,00021425
PURB	4	0,000216134
SNRNP70	4	0,000216372
RILPL1	3	0,000217957
PEX19	1	0,000218977
CHMP3	1	0,000220351
TNIP1	3	0,000222294
ZNF394	4	0,00022395
ABCB11	1	0,000225492
TXNRD1	1	0,000225807
CENPO	1	0,000226768
LAMC1	1	0,000226804
BTG3	4	0,000227267
CLDN11	1	0,000227349
MAP4K3	1	0,000230297
PPM1B	5	0,000231736
AQP11	4	0,000235544
CTNNA2	1	0,000235617
HIST1H2AC	4	0,000238401
MED26	2	0,000239025
ERC1	1	0,000239541
CACNA1D	1	0,000240435
USP4	1	0,000240638
SUPT7L	1	0,000240823
ERLIN2	4	0,000241727
SF3A2	4	0,000242072
FOXN1	1	0,000242941
FLOT1	1	0,00024317
DHX16	4	0,000246087
STXBP4	1	0,000246231

KLK5	4	0,000246821
ZMYM2	1	0,000247866
HPS5	1	0,000248207
LEF1	1	0,000249467
BIVM	1	0,000249544
ATP6V1D	3	0,000250811
ZNF638	5	0,000254715
VPS39	1	0,000256582
NR1I2	1	0,000257252
ACBD4	1	0,000258758
SLC25A17	1	0,000258983
ARL15	1	0,00025936
CYB561A3	1	0,000261776
SKIL	4	0,00026302
TNK2	2	0,000264448
PTPN18	3	0,000268104
NOS2	3	0,000268699
DNMT3A	1	0,000268773
PSTPIP2	1	0,000271213
RHOG	3	0,00027384
DDC	1	0,000274945
LOC513767	1	0,000275213
PRKDC	1	0,00027556
EXOC3L1	1	0,000276507
MCM8	1	0,00027676
FLNB	1	0,00027693
RCC1	1	0,000277835
GABRG2	3	0,000279614
CPQ	1	0,00028001
NFIB	1	0,000281306
PRKG2	1	0,000281428
LEAP2	1	0,000281702
TMEM147	1	0,000284296
BCAR1	1	0,000286942
SH3GL2	1	0,000287426
LEPROT	1	0,000293434
RNF166	3	0,000293444
PLEK2	1	0,000293945
FAM84B	4	0,00029675
ZMAT4	2	0,000297496
MPDZ	1	0,000297529
C2H2orf76	1	0,000298242
SMARCD2	2	0,000298348
C7H19orf52	3	0,000298418
ACVR1	1	0,000298498
TSFM	3	0,00029891
DHX36	4	0,000299492
LBH	3	0,00029982
FUBP3	3	0,000301086
PDXDC1	1	0,000304578

APBA1	1	0,000304873
AFAP1L2	1	0,000310333
LYSMD3	1	0,000313597
STX6	1	0,000314644
CD3E	1	0,000317256
TWIST1	3	0,000319705
DENND4A	1	0,000320487
CC2D1A	3	0,000320958
TRIM27	4	0,000321588
REEP2	1	0,000322009
F2R	1	0,000322509
DNAJC10	4	0,000323441
FARSA	1	0,000326794
POLR3C	1	0,000328299
PSMD12	1	0,000328866
PDCD10	4	0,000329542
HINT1	3	0,000330337
CITED2	3	0,000330372
GLIPR2	4	0,000333292
LHX4	1	0,000333883
TTC27	1	0,000334101
DLG3	1	0,000334169
MYH10	1	0,000334831
DMAP1	1	0,000335113
KLHL42	1	0,000335113
SLC39A14	1	0,000335481
DYRK1B	1	0,000336631
VPS33A	1	0,000336854
STAR	3	0,000338742
MUTYH	1	0,000342625
CHPF2	1	0,000343627
CPN1	1	0,000343798
EIF3G	4	0,000344292
CCBL1	1	0,000346089
GMIP	1	0,000346875
BCL6B	1	0,000350495
CELF2	1	0,000352499
ZNF181	1	0,000354178
TM9SF2	3	0,000354732
PLS3	4	0,000356349
SPDYA	1	0,000357819
PUM1	4	0,000359711
ZNF451	1	0,000361054
ARHGEF2	1	0,000361596
BTD	1	0,00036272
TP53I3	1	0,000368459
CHMP4B	1	0,000370188
DEPTOR	1	0,000370919
HPS1	1	0,000371906
IP6K2	3	0,000375104

ACO1	3	0,000375764
DNAJC7	1	0,00037638
RB1	1	0,000379739
PDZD8	4	0,000382253
ESPL1	4	0,000382693
DNMT3B	1	0,000382884
SNCA	1	0,000383932
LIG4	1	0,000385396
RPL12	4	0,000386973
DDX3Y	4	0,000391242
CSNK1A1	5	0,000391538
ARCN1	1	0,000391842
LIG3	5	0,000392389
NES	1	0,000394242
AP1B1	1	0,000394792
PBK	1	0,000399423
MMAA	1	0,000399968
PACRGL	1	0,000400941
C6H4orf17	1	0,000401225
FEZ1	3	0,000402357
TUBE1	1	0,000404028
ZNF652	1	0,000406773
ATF7IP2	1	0,000406962
FAN1	1	0,000407619
TRAF4	3	0,00041091
FAM19A1	1	0,000411637
MRPL52	3	0,000411753
IBSP	1	0,000412778
PKM	1	0,00041317
ITGB1	1	0,000413962
ATF3	4	0,000414403
ATP2B1	1	0,000414713
NDUFS2	1	0,000414713
CCDC28B	1	0,000418751
SCFD1	1	0,000423575
STXBP5L	3	0,000423575
HYOU1	3	0,000423934
ARHGEF28	1	0,000424276
MXD1	1	0,000428454
STX18	1	0,000428593
TMEM115	1	0,000430229
HEXA	1	0,000430838
FAR1	4	0,000433064
SPRY2	1	0,000433432
KCNH1	3	0,000438327
NCR3LG1	1	0,000438386
ZNF148	4	0,00043842
TMED3	1	0,000439648
HMG20B	4	0,000443129
NUMA1	4	0,000444124

SARS2	1	0,000444448
PBXIP1	1	0,000447191
DIS3L	4	0,000448074
NDUFB5	1	0,000450421
BNIP3	2	0,000451035
THTPA	1	0,000451659
OXA1L	1	0,000453791
ZMPSTE24	1	0,000455244
VRK3	1	0,000455297
ZSWIM8	1	0,00045554
SMARCD1	1	0,000456556
PARP11	3	0,0004581
PEX2	3	0,00045904
DRC7	1	0,000461797
INIP	3	0,000463815
FXYD6	1	0,000466195
SERINC3	1	0,000466238
AS3MT	3	0,000471135
ATP6V1C1	3	0,000475792
SLC9A1	3	0,000476183
HID1	1	0,000477643
ASNS	1	0,000478559
EXOSC6	1	0,000480679
ANXA2	1	0,000481853
NSMCE4A	1	0,00048342
TBK1	1	0,000484349
JAM2	4	0,000488578
SEPT8	1	0,000490657
PPCS	1	0,000492257
EMC6	5	0,000493051
CBR4	1	0,000502653
NHS	1	0,000504278
STRAP	1	0,000504278
HSPB11	1	0,000505772
MRPL35	1	0,000506827
MYOM1	1	0,000509774
ADCY9	1	0,000515693
CEP135	1	0,00051857
EBF1	1	0,000521424
ABCC1	1	0,000523378
SDCCAG3	1	0,000524931
TPI1	1	0,000525274
DRG1	4	0,000525621
SNX27	1	0,000526143
METTL8	1	0,000528564
UNC5D	1	0,000530857
EEPD1	1	0,000532056
OCRL	1	0,000532497
UCP3	1	0,000535031
OCEL1	1	0,000536409

MINA	1	0,00053714
PLCG1	1	0,00053757
DCTN5	1	0,00054036
RND3	4	0,00054046
CAND1	4	0,000545091
VPS26B	1	0,000546898
CDK7	3	0,000551151
SEC22A	1	0,000558088
SUMO3	1	0,000561782
GNG4	1	0,00056234
CSPG5	2	0,000562945
CERS2	1	0,000564855
CREB3L2	3	0,000572122
CDH3	1	0,000572802
ARFGAP1	1	0,000575445
KLHL24	4	0,000581618
CD320	1	0,000582112
PANX1	1	0,000583163
TJAP1	1	0,000585372
ARPC1B	3	0,000585376
SLC41A2	1	0,000588025
RAD23A	3	0,000592331
CLSTN3	1	0,000593918
FREM1	1	0,000597638
COX19	1	0,000599185
TMEM59	4	0,000599185
ALG6	1	0,000599251
DTWD2	1	0,000599891
ZNF576	4	0,000602584
LRR1	1	0,000604464
ANAPC10	1	0,000604731
SPATA20	1	0,000606699
SGPL1	1	0,000610604
MED29	4	0,000611633
RASGEF1B	1	0,000611633
PAK3	1	0,000612241
COMMD6	3	0,000614368
VSIG1	1	0,000617052
PARK7	3	0,000618975
GABARAPL1	4	0,000620931
PLEKHH3	3	0,000620931
CDK5RAP2	1	0,000621599
LENG8	2	0,000625219
C8B	1	0,000627186
FKBP7	1	0,000627513
C26H10orf2	1	0,000627942
C7H19orf43	4	0,000628179
POC5	1	0,000628256
NSUN5	1	0,00063006
GOLM1	1	0,000639362

ASAP3	1	0,000639832
PALM2	1	0,000640652
DNAJB2	3	0,000643854
DPM2	3	0,000645625
C7H19orf24	4	0,000648972
STK19	4	0,000649401
EVI5L	1	0,000651512
SNX24	1	0,000657759
WDR89	4	0,000658722
EFEMP1	1	0,000665177
KCNA5	1	0,000665745
GJB4	1	0,000667669
FANCG	1	0,000668613
CREB1	3	0,000670215
PTPN3	1	0,000679465
MRPL58	3	0,000681868
MYL6B	3	0,000681868
BORCS5	3	0,000682494
ABTB1	4	0,00068612
ALAD	1	0,000688875
ADA	1	0,000691958
TFAP2B	1	0,000693144
NKD1	1	0,000700079
PROSC	4	0,000701712
NRF1	2	0,000703584
SLC35G1	1	0,000704667
FBLN5	1	0,000705818
SGSH	1	0,000712001
IRAK2	3	0,000719436
ST3GAL6	1	0,000726132
OTUD4	1	0,000731288
DHX32	1	0,000738355
ACOT8	1	0,000741256
TUBGCP5	1	0,000741795
MANBAL	1	0,000743638
GRB7	1	0,000747258
ITGA2	1	0,000748213
EMC8	3	0,00075419
HEY2	1	0,000756241
PCCA	3	0,000756324
GCHFR	4	0,000760013
OLFML1	1	0,000760013
GPS2	1	0,000762547
CCZ1	1	0,000763911
MED19	4	0,000766242
TANK	1	0,00077751
CDA	1	0,000782059
ERCC8	4	0,000782059
TAF6	1	0,000782059
PTPRB	1	0,000782544

GMCL1	1	0,000786218
MAP1B	1	0,000787971
AP1M2	1	0,000789005
TSG101	3	0,000790462
SESN2	1	0,000794723
TRIM13	3	0,000797137
ADCYAP1	5	0,000798986
FRMD3	1	0,000799709
LPGAT1	1	0,000799709
OSGEPL1	3	0,000801124
PRRG4	1	0,000804678
DNAJC21	1	0,000817104
UQCC	1	0,000827418
SDR42E1	1	0,00083575
MID1IP1	4	0,000837384
RASGRP2	1	0,000843978
PTBP2	1	0,000846781
CARS2	1	0,000848226
PTRH2	4	0,000848226
MTRR	1	0,000849417
MANBA	3	0,000852963
CD59	1	0,000855825
TMEM198	1	0,000866779
TRPV6	1	0,000870343
KIN	3	0,000871312
CETN3	1	0,000877933
FUT8	1	0,000881102
RTCB	1	0,000884315
S100A10	4	0,000885473
CLN5	3	0,00088637
MAPKAPK3	3	0,000889291
CA6	1	0,000893865
FGFR2	1	0,000893865
ATP1B3	4	0,000898234
CSTB	1	0,000898345
DGKA	1	0,000898902
CTNS	1	0,000899376
RAD54B	4	0,000901399
NUDT9	3	0,000904429
NDRG3	1	0,000907073
ITFG1	1	0,000907872
EIF4E3	1	0,000909339
MAK	4	0,000912199
KIAA0907	4	0,000914352
FBXO22	1	0,000918224
CRHBP	3	0,000924886
DTNB	1	0,000927969
CCS	2	0,000931689
PIK3CB	1	0,000932506
SSFA2	1	0,000944654

PRMT2	1	0,000953444
MRPL57	4	0,000957841
XAB2	1	0,000969506
GTF3C1	1	0,000973503
DPP10	3	0,000977004
CACNA1E	1	0,000983639
LAMP1	5	0,000983733
DTD1	1	0,000987627
CDKN1B	1	0,000989167
BHMT	1	0,000991396
TBX18	1	0,000991396
ACTC1	1	0,000992509
ACCS	1	0,000993645
LRRC45	1	0,000995538
LOC788142	1	0,000997921
NSUN6	3	0,001003067
EFHB	3	0,001005563
KCNK5	4	0,001011774
PGGT1B	1	0,00101807
C2H2orf47	1	0,001019056
BBS7	1	0,001021438
DDX28	1	0,00103224
UBE2G2	1	0,00103588
PTPN5	1	0,00103812
CMBL	1	0,001039467
KANSL2	4	0,001040723
PNOC	1	0,001049103
DHRS4	1	0,001053172
EXOG	1	0,001059754
MORN2	3	0,001069831
VMP1	4	0,001075127
SUN2	1	0,001083258
GLCE	1	0,001085248
PFN1	3	0,001097303
ERLIN1	1	0,001104823
KPNB1	1	0,001105711
PRRC1	1	0,001113334
LETMD1	3	0,001115626
SERGEF	1	0,001120244
PPP1R1A	1	0,001122844
HIP1	1	0,0011238
ARFIP1	1	0,00112506
HYKK	1	0,001129048
C20H5orf22	1	0,001129659
LOC512899	1	0,001132368
CNGA3	1	0,001134978
BDH1	4	0,001137132
LOC534913	3	0,001140105
RAD1	1	0,00114752
POR	1	0,001148283

FAM13A	1	0,001152642
ATP6V1C2	1	0,001154022
POLR1B	1	0,001156099
TMCC2	3	0,001161699
SFXN4	1	0,001164924
P4HA1	1	0,001166141
GPR179	1	0,001171625
APIP	1	0,001173245
SMYD3	3	0,001175321
PTCD3	1	0,00118411
FERMT3	1	0,001184243
NOS1AP	1	0,001185229
ARPC3	1	0,001187666
SOCS4	4	0,001187666
DYNC1I1	1	0,001189114
TSKU	1	0,001190236
KLHDC9	1	0,001209096
POLR2I	4	0,001212211
LIX1L	3	0,00121915
BCAT1	3	0,001221185
P2RX1	1	0,001230863
ARFGAP2	1	0,001235998
RPS19BP1	4	0,001236847
STK3	5	0,001240878
SF3B3	4	0,001241507
LDLR	1	0,001246172
JAK1	1	0,001263167
MSX2	1	0,00127546
FGFR1OP2	4	0,001276471
NEK3	1	0,001283079
SFRP1	1	0,001285736
KLHDC10	1	0,001287066
SDHAF2	1	0,001289409
ZNF341	1	0,001289409
ILDR2	1	0,001289996
BLOC1S6	3	0,001291581
PPP2CB	4	0,001292312
ABHD6	1	0,001296917
BTRC	3	0,001296917
IFT81	1	0,001297461
PTN	1	0,001301648
NAP1L4	1	0,001302145
IQSEC1	3	0,001302516
SGCB	1	0,001310702
PSMD3	1	0,001315518
STIM1	1	0,001316926
GABPB2	1	0,001322149
CLCN4	1	0,001322389
LOC616319	1	0,001323658
WNT2B	1	0,001327094

ZKSCAN8	4	0,001330252
NHEDC1	1	0,001331468
IDI1	1	0,001356693
SCLY	4	0,001369267
BICDL2	3	0,001370153
TNPO3	4	0,00137056
DOLK	3	0,001375957
ADGRL3	1	0,001378666
CTU1	4	0,001391189
LATS1	1	0,001399899
TTC21B	1	0,001401645
GAN	4	0,001401712
MAPKAP1	1	0,001402318
WDR70	5	0,001411445
CPTP	3	0,001416147
GPC5	1	0,00141667
EHD4	1	0,001443884
HMG20A	1	0,001453555
UMPS	4	0,001456429
SAR1B	1	0,001471159
NDUFA5	1	0,001475525
SLC39A7	1	0,00148448
CUX2	3	0,001485206
FNDC3A	1	0,001486772
HSPBAP1	1	0,001495812
USO1	1	0,001499125
MMACHC	4	0,001500755
LAMTOR5	1	0,001504046
CASC1	1	0,001505699
PCBD1	1	0,001541544
PTPRR	1	0,001542787
FNTB	1	0,001543277
ZHX2	1	0,001561492
NAV3	1	0,001562455
LRRFIP1	1	0,001574612
SLC5A6	4	0,0015859
CNEP1R1	3	0,0015873
ZNF445	1	0,0015873
RASGEF1A	3	0,001603422
UBE2D4	1	0,001619135
MRPS2	3	0,001630016
RFX5	3	0,001632001
AK3	1	0,00163607
HMMR	1	0,00163607
ARL2BP	1	0,001647093
SPIN1	4	0,001663772
HARBI1	1	0,001673252
MSI1	1	0,00167601
RWDD2A	1	0,00167601
SIGMAR1	1	0,00167601

CNOT10	3	0,001676741
SULF2	1	0,001676987
FARS2	1	0,001678171
HSPA1L	1	0,001692895
CMTR1	1	0,00170005
TLR2	5	0,001701931
MRPL48	1	0,001708364
TBL2	1	0,001709682
GCNT3	1	0,001711203
FBXL14	1	0,001713199
MGAT4B	3	0,001715447
ACACA	1	0,001720605
PPP3CB	1	0,001723207
ZFYVE27	4	0,001729542
EXTL3	1	0,001731609
C5	1	0,00173381
FAM92A1	3	0,00174717
GATB	3	0,001771756
FAT1	1	0,001772161
MCTP1	3	0,00177239
RNF7	1	0,001773873
ATP5B	4	0,001775351
PPP1R1B	1	0,001780863
SLC31A2	1	0,001789812
ULK3	1	0,001813118
GTSE1	1	0,001817496
MGAT4A	1	0,001820852
TPPP3	1	0,001836349
SPCS1	3	0,001846298
VIPAS39	1	0,001847185
WLS	3	0,001863281
PHC2	1	0,001863821
GOLGA7B	3	0,001876497
EIF2S1	1	0,00187794
DNM3	1	0,001882042
HSPD1	1	0,00188868
SNX22	1	0,00188868
ANKRD1	3	0,001891488
EMG1	4	0,001899242
TMEM126A	1	0,001900033
ASL	1	0,001903363
ALDH3A2	1	0,001906221
GPX8	1	0,001906221
FRZB	1	0,001923991
MRPS9	3	0,001923991
USP44	1	0,001935283
ITPA	3	0,001936121
PTGER4	4	0,001941848
TFB1M	1	0,001954803
MRPS28	3	0,001992206

PDE12	4	0,002000459
TMEM127	1	0,002012563
PNPLA6	1	0,002013716
POLR3F	4	0,002017029
DYM	3	0,002035929
RPL7L1	4	0,002046128
NUP155	4	0,002047426
ZSWIM7	1	0,002052313
GET4	1	0,002056397
VRK2	1	0,002056397
BAHD1	3	0,002059231
HESX1	1	0,002059231
RAB28	1	0,002059231
ZSCAN26	1	0,002069557
OGDHL	1	0,002093648
JAG1	1	0,002099358
PPM1L	3	0,002106886
YARS	4	0,002106886
ACRV1	1	0,002116778
BANP	3	0,002116778
ORAI1	1	0,002116778
ZNF774	1	0,002117173
PORCN	3	0,002118503
SUGP1	4	0,002119984
FBXO18	1	0,002130756
SYT4	3	0,002160627
MAPK9	1	0,002170669
TRAPPC3	1	0,002175536
ADORA2B	1	0,002186329
H1F0	1	0,002198201
KIAA2012	1	0,002199535
KCNMA1	1	0,002202221
FXR2	1	0,002202783
B4GALT6	1	0,002214889
C7H5orf24	1	0,002215002
TMEM41A	3	0,002237782
COPS5	3	0,002239304
DEUP1	1	0,002243772
UBE3D	1	0,002243987
EIF2B1	1	0,002252515
ZNF621	1	0,002267501
BROX	4	0,002280057
LOC788201	1	0,002289312
CDC42BPG	1	0,002293615
ARL8B	1	0,00229451
FBF1	3	0,00230526
MICAL1	1	0,002309884
TDRKH	1	0,0023118
SNX3	1	0,00231542
RFWD2	1	0,002317541

CMPK1	1	0,002324502
RPL18	4	0,002330531
CDC7	1	0,002330644
PKIG	1	0,002330644
MLF1	1	0,002331154
SLC46A1	1	0,002337003
GTF2E1	4	0,002339682
LRIG3	1	0,002346632
CBLB	1	0,002346836
ANKRD27	1	0,002348237
EMILIN2	3	0,002355818
NAE1	1	0,002367687
DIP2B	1	0,002369921
EMC7	1	0,002369921
RHPN2	4	0,002369921
ARSB	1	0,002370004
PTMS	3	0,002373189
ABCA4	1	0,002383933
BCL7C	3	0,002387085
BLOC1S5	3	0,002387085
MRGPRX2	4	0,002387115
STMN1	3	0,002387115
ITGA6	1	0,002397557
BRIX1	1	0,002413713
C7H19orf66	1	0,002413713
RASD2	3	0,002414695
UXS1	3	0,002419785
IMPA1	3	0,002424157
CECR5	1	0,00242905
MTERF3	6	0,002435834
NDUFA7	1	0,00244509
HHEX	1	0,002467904
RAB3C	3	0,002473661
ATP6V0A1	1	0,002479199
INPP5K	4	0,002481063
LOC515736	1	0,002496922
DVL1	1	0,00249924
AIM1L	1	0,002519286
CSTF2T	4	0,002522574
C9H6orf203	1	0,002528452
GPR84	3	0,002533141
TUBGCP3	3	0,002533141
NAIF1	4	0,002542512
PPT1	1	0,002542512
GFRA3	4	0,002544483
SLC3A2	1	0,002551391
C10H14orf37	1	0,002556731
DECR1	1	0,002590619
TMEM55B	3	0,002601897
MLN	3	0,002608544

RARRES1	3	0,002616015
FARP1	1	0,002625609
ZNF384	2	0,002633626
USP15	5	0,002642667
KCNH5	1	0,00266065
CSK	1	0,002670467
UNC93B1	3	0,002677155
NAA60	3	0,002678542
MAN2A1	1	0,002692364
BLOC1S1	3	0,002693075
APOBEC2	3	0,002698753
ECHS1	1	0,002707442
COMMD7	1	0,002722133
CNKSR3	1	0,002723949
ZNF689	3	0,002725352
ATG7	1	0,002725926
ZCCHC4	1	0,002730235
NDUFA9	1	0,002741827
C16H1orf27	1	0,002761189
SFXN1	1	0,002762926
TAB1	3	0,002765929
TEK	1	0,002780371
ZDHHC13	1	0,002789557
CCDC115	4	0,002794578
SOX2	3	0,002796215
NAAA	3	0,002797539
SUPT3H	1	0,00281543
CALCOCO2	1	0,002823739
ADPRM	4	0,002832946
PPP1R3F	3	0,002838523
PNRC2	3	0,002841993
ERP44	1	0,002844677
MIOS	1	0,002852477
SLC8B1	1	0,002853963
WDR54	1	0,002859461
PPIP5K1	1	0,002862093
PIP4K2B	1	0,002864164
MRPS27	1	0,002869858
GPN3	4	0,00287423
DAPK1	1	0,00290186
SRSF9	3	0,00290186
IGFLR1	4	0,002906477
LMO2	5	0,002922058
EPN2	1	0,002936998
SMIM14	1	0,002954828
TRIM32	1	0,002964788
PSMB6	4	0,002967296
RPS6KC1	1	0,002969075
TRPC6	1	0,002970809
PHLPP2	1	0,002981126

LETM1	3	0,00298344
CHRNA7	1	0,002993913
MTMR10	1	0,003008076
TUBB6	1	0,003021367
ABR	3	0,003024952
ACSS1	4	0,003028142
SIAE	1	0,003035456
KIF4A	5	0,003072853
TDP2	1	0,003075672
CPNE3	1	0,003086111
SYN3	3	0,003160119
CALB2	1	0,003164618
METTL18	1	0,003171404
COX10	1	0,003171513
CSNK1E	3	0,003171513
SPCS3	4	0,003185127
RPA2	1	0,003189359
RFC3	3	0,003211983
HSPB1	3	0,003228392
OGDH	1	0,003234315
CCAR2	1	0,003265077
TRMT12	3	0,003270534
DONSON	1	0,003278974
MRPL47	3	0,003298548
NCAPD2	4	0,00330789
CDK16	1	0,003342163
OGFR	1	0,003382965
RNF121	3	0,003393082
NCSTN	3	0,003394624
CDC42EP4	1	0,003420978
LTA4H	3	0,003423215
NQO2	1	0,003425797
NUCKS1	3	0,003431093
ECD	1	0,00345243
ADAMTS12	1	0,003452449
TMED5	4	0,003452449
PKN2	1	0,003458495
KIF3B	3	0,003458929
UBE2D1	1	0,003507763
CASC3	1	0,003508257
RPAP2	1	0,003508257
GTF2A1L	1	0,003513407
TTL	1	0,003517372
KLHL20	1	0,003539308
LYRM9	1	0,003548049
AGO3	4	0,003553701
VCAN	3	0,003557973
QDPR	3	0,003580778
PIGM	4	0,003582393
LOC533308	1	0,003599048

DDX6	1	0,003613049
CKMT2	3	0,003633953
TSR3	3	0,00364131
SCG5	1	0,003656384
PAQR7	3	0,003660115
ATP5G1	1	0,003678582
MRPL46	1	0,003705062
STAT1	1	0,003707165
WDR77	4	0,003708953
TMCO3	3	0,003714449
ZBTB11	5	0,003716877
SUMF1	1	0,003753354
UTP6	4	0,003756592
BCAR3	1	0,003762176
PCGF5	1	0,003762176
VAPB	3	0,003772599
BID	1	0,003773977
IER2	4	0,003775639
CTDSPL	1	0,003781034
SNX5	4	0,003824179
MYD88	1	0,003851229
ZC4H2	3	0,003874484
FAM81B	1	0,003884988
RNF128	4	0,003894887
IVD	1	0,003897645
TBC1D9B	3	0,003898072
RILPL2	1	0,003932757
TMEM106B	3	0,003937765
PITPNA	1	0,00394235
C1GALT1	4	0,00394665
HLF	3	0,00394665
SNX30	1	0,003948709
RUFY3	1	0,003953793
PIK3R1	1	0,003963108
SLC44A3	1	0,003981263
TMEM206	1	0,003981263
FAF2	1	0,004013911
ZNF705A	3	0,004015428
RAD9A	1	0,004019672
HECTD2	1	0,004023016
HAT1	4	0,004029957
CPOX	1	0,004035896
EPB41L3	1	0,004035896
CIDEA	1	0,004043
NUDT16	1	0,004050245
PSTK	1	0,004058357
GCAT	1	0,004063879
ZSCAN31	1	0,004087927
EIF2A	4	0,004096651
ETV5	1	0,004096651

SYTL4	3	0,004109118
CSNK2A2	3	0,004117196
SMG5	4	0,00411936
NXT1	1	0,004122344
TRAT1	3	0,004131262
NOSTRIN	1	0,00413377
AARS	4	0,004183632
PHF1	3	0,004185119
EXOSC4	1	0,004218751
CLASP2	1	0,004237858
UXT	4	0,004269979
BSP3	1	0,004271747
PSMG2	3	0,004288392
STRN3	5	0,004295918
SLA2	1	0,004323241
RNF138	4	0,004354793
HINFP	4	0,004355642
HIST3H2A	1	0,004363319
ARRDC4	1	0,004406088
HDDC3	1	0,004407276
MRPS5	1	0,004420822
DEF8	1	0,004425203
CLN8	3	0,004432803
HMBOX1	1	0,004438646
B4GALT3	5	0,00444504
POLR2C	1	0,004459415
TMEM35	3	0,00447635
UBALD1	4	0,004501876
PFKFB2	1	0,004509682
BUD13	3	0,004580206
SORCS1	1	0,004580982
STC1	3	0,004580982
RPL14	4	0,004581392
LRRTM1	1	0,004585915
ORMDL3	2	0,004587156
TAOK2	1	0,004587156
RNF123	3	0,004610083
NKIRAS2	1	0,004613137
CARD14	4	0,004614853
LRCH4	1	0,004630865
COQ3	4	0,004640492
EIF2B2	4	0,004663528
WDR91	1	0,004666358
DBT	1	0,004668487
ADGRE5	1	0,0047068
GAS7	1	0,004714886
NXN	3	0,004717503
MEST	1	0,004722657
FAM210B	3	0,00473322
PRIM2	1	0,004748879

PDE6A	1	0,004753185
HEATR6	1	0,004769451
C14H8orf33	4	0,004779259
IL17RB	1	0,004779259
BDNF	1	0,004822306
DIS3L2	1	0,004832182
CNST	1	0,004865929
PPP2R3C	1	0,00486836
TOR2A	1	0,004875266
CYCS	1	0,004908616
TUBA4A	4	0,004918124
BCKDHB	1	0,004918712
NOL12	1	0,004939072
DPPA4	4	0,004966559
C15H11orf70	1	0,004966675
LRP10	4	0,004967348
TLE1	4	0,004982855
STK33	1	0,00499298
C7H5orf30	4	0,005001948
PRPF31	4	0,005003887
BARHL2	1	0,005063582
ENHO	1	0,005069613
TOR1AIP1	1	0,005069613
NUP188	1	0,005095838
RAB32	1	0,005099532
PLA2G16	1	0,005105095
TBC1D7	3	0,005105095
TMEM163	3	0,005105095
KCNE1	1	0,005106827
LOC534155	4	0,005112424
ISY1	3	0,005113543
PRPF4B	4	0,005174474
TBC1D20	3	0,005202385
FAM228B	1	0,005208985
HSPA2	5	0,005212466
PHPT1	1	0,005219872
PRMT7	1	0,005236894
ARRDC1	1	0,005240916
PIGS	1	0,005276172
CDX1	3	0,005282702
SLC35A5	3	0,005282702
LPCAT4	3	0,00529033
RAC3	1	0,005316631
STAM2	4	0,005322059
SARAF	1	0,005344972
POLA1	1	0,005402
TRAIP	1	0,005402
GRM1	1	0,005433269
SLC35F5	1	0,005433269
LYSMD4	1	0,005435278

STX8	2	0,005448837
TRPM7	1	0,005454134
DTNBP1	3	0,00546821
KRTCAP3	1	0,005579776
SEC14L1	1	0,005589311
CAPZB	1	0,005637545
GALNT2	1	0,005678233
SDCBP	3	0,005715446
TEX30	1	0,005744609
METTL21B	3	0,005803238
ADCY7	1	0,005812579
PIGW	4	0,005821165
TP63	3	0,005821165
DCK	3	0,005826727
ALG2	4	0,005839595
ORC2	1	0,005863028
DBN1	3	0,005866494
MEGF10	1	0,005867479
PDHX	1	0,005887593
CDX2	1	0,00590016
UBTD2	1	0,005905155
FH	1	0,005931983
HIRIP3	1	0,005948812
MRPL10	3	0,005951717
RNF214	3	0,005960119
WBP4	1	0,005974885
PATL1	5	0,006016422
RXRA	1	0,006079207
MRPS22	1	0,006095436
ZCCHC11	3	0,006097608
AIMP2	4	0,006108042
ACADS	1	0,006119905
AZIN2	3	0,006148145
GFI1B	1	0,006148145
TRMT11	1	0,006167192
NT5C3B	1	0,006176911
MFF	3	0,0061882
MED25	1	0,006212317
SQSTM1	1	0,006261512
MYL6	1	0,006320268
TRMT10B	3	0,006348766
SETDB2	4	0,006351109
ALDH5A1	3	0,006366306
TRIM37	1	0,00637258
ACOT13	1	0,006384032
MRRF	1	0,006393316
KIAA1551	4	0,006399854
PPRC1	4	0,006402607
GATA2	1	0,006429783
PSMC3IP	3	0,006450633

DPP3	3	0,006484501
DUSP4	2	0,006484501
SMYD2	1	0,006544242
MYCBP2	1	0,006571798
PCED1A	1	0,006580498
PLA2G4D	1	0,006589775
ANKRA2	1	0,006604496
PAFAH2	1	0,00660695
RBFOX1	1	0,006625548
HSF2BP	1	0,006659799
ING2	2	0,006671385
KRAS	1	0,006679518
MTMR7	1	0,006679518
NENF	3	0,006753953
TRAPPC11	1	0,006781487
CAPNS1	4	0,006790189
CHCHD6	1	0,006799349
NAGK	1	0,006854768
MBOAT1	1	0,006897043
PDCL2	3	0,006904875
C5H12orf4	1	0,00690507
CCR8	1	0,006920098
ZPR1	4	0,006931019
MSN	1	0,006936196
RBMX2	1	0,006953672
MIP	1	0,006979838
CTNNA3	3	0,006984334
MIS12	4	0,007002246
ZDHHC21	1	0,007019107
C11H9orf16	4	0,007019127
RNF185	4	0,007092415
CDKN2AIP	4	0,007143869
PIK3C3	1	0,007146646
CAST	2	0,00714819
GARS	1	0,007153285
CPA1	1	0,007163567
RALGPS2	3	0,007168215
DUSP11	4	0,007171553
GMPR	1	0,007178558
RLIM	4	0,007191088
WDR19	1	0,007195182
DGUOK	3	0,007219138
EXOC1	1	0,007236437
LIPT2	1	0,007260711
RAB26	3	0,007260711
ZMYM3	1	0,007279177
FRMD5	1	0,007401861
KMT5A	4	0,007410414
RIOK2	1	0,007412675
URB1	4	0,007416699

SMCHD1	4	0,007417914
ZC3H4	1	0,007442828
ABCF3	4	0,007452537
MAN1A1	1	0,007473662
CSTF2	1	0,007473845
ZNF570	1	0,007514198
ZBED5	1	0,007569151
GCLC	1	0,007581257
NPRL2	1	0,00763053
MAB21L1	1	0,00765764
ATG4C	4	0,00767663
MUM1	3	0,007677033
TEFM	1	0,007710682
C21H14orf2	3	0,007731225
TTYH1	3	0,007764028
GRK2	1	0,007862783
LIX1	1	0,007877377
ELN	3	0,007941771
NSFL1C	1	0,007941771
FEM1A	4	0,007970337
CHPF	1	0,007997016
ITPRIP	4	0,008027605
HK1	2	0,008072913
RNF122	1	0,008074664
POLA2	1	0,008142728
TRAPPC10	3	0,008157375
LIN7B	1	0,008172137
MSH6	4	0,008182419
CDYL	1	0,008190213
PYURF	3	0,008229837
RNF43	1	0,00824559
PPP1R13L	1	0,008263941
FGF16	1	0,008344226
NADK	1	0,00837861
ARIH2	3	0,008411697
CTPS1	1	0,008411697
PI4K2B	1	0,008429291
NT5DC1	3	0,008447511
IQCD	4	0,008488485
EIF4EBP2	5	0,008548759
C24H18orf25	1	0,008562681
IL2RA	1	0,008562681
PSAT1	1	0,008568754
TSPAN33	1	0,00858584
DNAJC18	1	0,008618209
RPRD1B	4	0,00864536
NCKIPSD	1	0,008670522
COPS6	1	0,008683954
FAT2	1	0,008746286
SRF	3	0,008761545

TRAP1	3	0,008781144
GNG12	1	0,008811502
RPF2	1	0,008888981
WDR6	4	0,008916522
UBL4A	1	0,008925377
IAH1	1	0,008949835
NDUFS8	3	0,008994069
MBTD1	5	0,009023039
TMX1	4	0,009023039
UPP2	1	0,009069447
CPE	1	0,009103802
IL2RG	4	0,009160343
ACACB	1	0,009180295
ZNF329	1	0,009195134
FECH	1	0,009258712
RAD51B	1	0,009307576
DCLRE1C	1	0,009313812
CCDC36	1	0,009345922
RGL1	1	0,009398127
RND2	1	0,009436791
VPS50	1	0,009540973
DFNA5	1	0,009551717
FCF1	3	0,009551717
MRPL41	4	0,009551717
DNASE1	4	0,009572089
ODF2L	1	0,009585034
NUDT11	3	0,009603152
MCRS1	4	0,009605893
ARHGEF37	1	0,00961075
C9H6orf118	3	0,009614828
PRKACA	3	0,009622304
TNFSF8	1	0,0096315
SNX29	1	0,009667983
CMTM4	1	0,009687578
ARHGEF16	1	0,009704424
TUG1	4	0,009735821
PRKCE	1	0,00974598
SMIM12	4	0,009763965
PARVG	1	0,009767631
PDSS1	1	0,009779268
GLDC	1	0,009803759
MRPL16	4	0,009843099
NPAS2	1	0,009848027
RPS6KA3	1	0,009848027
EOGT	1	0,009917712
SMG6	1	0,00997363
RPTOR	1	0,009990719

Supplementary Table S3. Cluster specific marker genes identified using SC3 pipeline with a threshold of the adjusted p-value < 0.01 and the area under the ROC curve (AUROC) > 0.85.

Gene name	adjusted p-value	AUROC	Cluster
C8H9orf64	5,78322E-11	0,868639668	1
COG2	3,18661E-10	0,860159225	1
DPP4	1,09151E-10	0,859813084	1
F3	1,01963E-10	0,871408792	1
NETO1	5,25661E-11	0,857995846	1
RUFY2	5,26725E-10	0,859813084	1
TIGAR	2,2572E-10	0,864053306	1
TMEM128	5,35744E-10	0,859640014	1
TPMT	1,85153E-11	0,869505019	1
UCHL1	4,81329E-10	0,853409484	1
XRCC2	1,12258E-11	0,858428522	1
ZNF75A	4,96539E-10	0,857130495	1
RHEBL1	8,21539E-06	0,855979644	2
AMIGO2	8,57864E-06	0,865198711	4
AMT	1,35901E-05	0,85754565	4
ARFGAP3	3,34991E-07	0,895005371	4
ARHGAP1	6,1081E-06	0,866272825	4
ARMT1	4,12503E-07	0,888829216	4
ATF7IP	4,1745E-06	0,872180451	4
C14H8orf59	1,60997E-07	0,900912997	4
C1D	6,31323E-07	0,889366273	4
C3H1orf52	1,0613E-05	0,863050483	4
CAD	1,32611E-08	0,918367347	4
CCDC126	8,25557E-07	0,886949517	4
CCT4	5,17188E-06	0,870032223	4
CDK1	9,85311E-06	0,863856069	4
CDKN2C	1,95214E-05	0,851638024	4
CXADR	3,19081E-05	0,851235231	4
DESI2	2,11316E-07	0,889500537	4
DLD	3,41191E-05	0,851235231	4
EIF3D	1,54396E-05	0,859291085	4
EIF6	5,42133E-07	0,891245972	4
EXOSC1	5,09899E-06	0,870032223	4
FAM207A	7,47777E-08	0,886815252	4
FAM89A	1,14757E-06	0,880773362	4
FOLR1	3,65359E-08	0,914607948	4
GART	5,55874E-06	0,868689581	4
HAX1	5,46665E-07	0,890708915	4
HIST1H2BD	7,6562E-09	0,926154672	4
IL18	1,33071E-07	0,89943609	4
IMP3	1,28889E-07	0,90386681	4
IPMK	1,66538E-05	0,852712137	4
ISG20L2	8,56319E-08	0,892722879	4
ITM2B	3,01123E-08	0,900778733	4
KLF3	5,76141E-06	0,859828142	4
KLF5	5,61358E-07	0,888426423	4
LARP4	5,79018E-06	0,86895811	4
LOC782781	1,24401E-07	0,901718582	4
LRRC40	2,1534E-06	0,87755102	4
LSM8	3,8289E-06	0,871911923	4
MANF	7,06591E-06	0,855263158	4
MBIP	6,54952E-06	0,867883996	4
MED31	1,98476E-05	0,856068743	4
MRPL17	1,98026E-07	0,90037594	4

MTHFD1L	2,35239E-07	0,895542427	4
NAA11	9,59681E-07	0,883458647	4
NANOG	2,21636E-06	0,861976369	4
NCBP2	8,23006E-07	0,887486574	4
NDUFB2	1,39641E-06	0,882384533	4
NDUFB3	3,04196E-06	0,870435016	4
NKAPL	6,17809E-07	0,88990333	4
NOL6	1,26957E-06	0,883190118	4
NOP16	1,00822E-06	0,883458647	4
PARP14	8,49937E-07	0,87150913	4
PDCD10	1,04396E-05	0,863050483	4
PHF5A	1,90154E-05	0,855397422	4
PIP5K1A	2,52619E-05	0,853383459	4
PLEKHJ1	1,42305E-05	0,857411386	4
PPAT	9,59053E-06	0,863990333	4
PPIL1	3,90162E-06	0,855934479	4
PRPS1	2,5436E-05	0,853920516	4
PSMA1	3,08411E-06	0,862916219	4
PYCR2	2,77566E-05	0,853383459	4
RACK1	4,44504E-06	0,871643394	4
RNF11	7,78745E-07	0,888023631	4
RPL26	2,97325E-06	0,875402793	4
RPL36AL	4,45686E-06	0,871643394	4
RPL37	6,89829E-07	0,888560687	4
RPL38	7,635E-06	0,866272825	4
RPS19	3,95417E-08	0,91433942	4
RPS27	3,00288E-07	0,896616541	4
RPS29	6,74663E-07	0,889366273	4
RPS4Y1	7,33016E-07	0,888560687	4
RPS9	3,75967E-06	0,87244898	4
SCO1	3,90642E-06	0,862379162	4
SLC27A1	1,8594E-05	0,852577873	4
SLC33A1	2,57437E-08	0,90829753	4
SNHG12	3,3282E-07	0,887218045	4
SNRPB	5,43696E-06	0,869495166	4
SYT11	2,96711E-05	0,852846402	4
TAF11	8,12336E-06	0,86546724	4
TCEB2	1,32201E-06	0,882384533	4
TIMM10B	1,75964E-07	0,885606874	4
TMEM41B	2,86018E-05	0,852309345	4
TSC22D1	3,20347E-05	0,852040816	4
TSEN54	5,12698E-06	0,866004296	4
TTC14	7,33487E-07	0,888292159	4
TYW3	3,5239E-06	0,865601504	4
UQCRFS1	1,66106E-06	0,878625134	4
ZBTB9	1,69814E-06	0,880504834	4
ZNRF1	1,66365E-06	0,879699248	4
GRO1	4,91E-006	0,860767991	5

Supplementary Table S4. GO terms related to the six topics identified by using SC3 tool.

GO-ID	GO-term	Corrected p-value	No. genes
GO:0042254	ribosome biogenesis	2,90E-06	9
GO:0042255	ribosome assembly	2,90E-03	3
GO:0042273	ribosomal large subunit biogenesis	3,60E-03	3
GO:0046112	nucleobase biosynthetic process	4,20E-03	3
GO:0006414	translational elongation	4,60E-03	3
GO:0008652	cellular amino acid biosynthetic process	5,40E-03	3

Supplementary Table S5. GO terms of the different topics, obtained by using CellTree tool.

Topic	GO-ID	GO Term	Corrected p-value	No. genes
1	GO:0006412	translation	1,50E-06	286
1	GO:0051301	cell division	2,20E-06	151
2	GO:0006412	translation	4,10E-18	286
2	GO:0006446	regulation of translational initiation	1,30E-08	29
2	GO:0000398	mRNA splicing, via spliceosome	1,70E-07	79
2	GO:0002181	cytoplasmic translation	5,60E-07	22
2	GO:0006364	rRNA processing	3,10E-06	74
2	GO:0000245	spliceosomal complex assembly	5,20E-06	14
2	GO:0048025	negative regulation of mRNA splicing, via spliceosome	5,60E-06	8
2	GO:0051301	cell division	1,10E-05	151
2	GO:0000381	regulation of alternative mRNA splicing, via spliceosome	1,90E-05	16
3	GO:0006412	translation	2,20E-06	286
4	GO:0015986	ATP synthesis coupled proton transport	7,10E-04	18
5	GO:0070125	mitochondrial translational elongation	6,50E-05	74
6	GO:0015850	organic hydroxy compound transport	3,00E-04	20

Supplementary Table S6. Subset of twenty genes analysed for studying the major embryonic genome activation (EGA). The genes and their normalised log2(UMI+1) values are reported.

Cell_Name	E1.1_p3	E1.2_p3	E1.3_p3	E1.5_p3	E1.6_p3	E1.7_p3	E14.1_p3	E14.2_p3	E14.3_p3	E14.4_p3
Embryo_Name	Day2-E1	Day2-E1	Day2-E1	Day2-E1	Day2-E1	Day2-E1	Day2-E2	Day2-E2	Day2-E2	Day2-E2
ADAMTS1	0,00000	0,00000	0,00000	0,00000	0,00000	0,67110	3,50328	0,00000	0,00000	0,00000
C3	0,00000	0,00000	0,00000	0,00000	0,00000	0,00000	0,00000	0,00000	0,00000	0,00000
CNR2	0,00000	0,00000	0,00000	0,00000	0,00000	0,00000	0,00000	0,00000	0,00000	0,00000
CRABP1	0,00000	0,00000	0,00000	0,00000	0,00000	1,18876	0,00000	0,00000	1,38160	0,00000
DDX3Y	2,43825	3,86615	0,00000	1,13034	0,20664	4,62469	5,77262	1,83990	1,47810	0,17612
DPPA4	0,55656	0,00000	0,00000	0,00000	0,00000	0,00000	1,19126	0,00000	0,00000	0,00000
FOLR1	0,24529	1,94864	0,00000	0,00000	0,00000	0,21505	2,76916	0,00000	0,92678	0,25927
GAT	0,00000	0,00000	0,00000	0,00000	0,00000	0,00000	0,00000	0,00000	0,00000	0,00000
GFRA3	0,00000	0,00000	0,00000	0,00000	0,00000	0,00000	0,00000	0,00000	0,00000	0,00000
LOC508834	0,00000	0,34869	0,00000	0,00000	0,00000	0,16610	4,41607	0,00000	0,00000	0,24595
LRRC14	0,78762	0,00000	0,00000	0,00000	0,00000	1,16807	0,00000	0,00000	0,00000	0,00000
MAGEH1	0,00000	0,00000	0,00000	0,00000	0,00000	0,00000	0,00000	0,00000	0,00000	0,00000
MESDC1	0,00000	0,00000	0,00000	0,00000	0,00000	0,00000	0,00000	0,00000	0,00000	0,00000
NANOG	0,00000	0,67110	0,00000	0,00000	0,00000	0,93555	2,44839	0,00000	0,00000	1,14474
NRBP2	0,00000	0,00000	0,00000	0,00000	0,00000	0,00000	0,00000	0,00000	0,00000	0,00000
OVOL2	1,64048	0,00000	0,00000	0,00000	0,00000	0,55796	1,42322	2,21397	0,00000	0,00000
PCDH10	0,00000	0,00000	0,00000	0,00000	0,00000	0,00000	0,00000	0,00000	0,00000	0,00000
PTGDR	2,67432	0,00000	0,00000	0,00000	0,00000	0,00000	1,05318	0,00000	0,00000	0,00000
SH2D3A	0,00000	0,00000	0,00000	0,00000	0,00000	0,00000	0,00000	0,00000	0,00000	0,00000
SLC2A5	0,00000	0,00000	0,00000	0,00000	0,00000	0,00000	0,00000	0,00000	0,00000	0,00000

Cell_Name	E14.5_p3	E14.6_p3	E14.7_p3	E14.8_p3	E14.9_p3	E16.1_p3	E16.2_p3	E16.4_p3	E16.6_p3	E16.7_p3
Embryo_Name	Day2-E2	Day2-E2	Day2-E2	Day2-E2	Day2-E2	Day2-E3	Day2-E3	Day2-E3	Day2-E3	Day2-E3
ADAMTS1	0,00000	0,00000	0,79088	0,00000	0,00000	1,00321	0,00000	0,00000	0,00000	0,00000
C3	0,00000	0,00000	0,00000	1,04184	0,00000	0,00000	0,00000	0,00000	0,00000	0,00000
CNR2	0,00000	0,00000	0,00000	0,00000	0,00000	0,00000	0,00000	0,00000	0,00000	0,00000
CRABP1	0,00000	0,00000	0,92325	0,00000	0,00000	0,00000	0,00000	0,95255	0,00000	0,00000
DDX3Y	0,84410	3,10329	2,80570	1,32973	0,00000	4,11920	3,54740	1,61735	3,78990	4,57516
DPPA4	0,00000	0,85679	0,00000	0,00000	0,00000	0,00000	0,00000	0,00000	0,86264	0,00000
FOLR1	1,24762	0,00000	2,42825	0,48920	0,00000	1,58496	1,83668	0,68228	0,81194	4,49400
GAT	0,00000	0,00000	0,00000	0,00000	0,00000	0,00000	0,00000	0,00000	0,00000	0,00000
GFRA3	0,00000	0,00000	0,00000	0,00000	0,00000	1,50359	0,00000	0,00000	0,00000	0,00000
LOC508834	0,00000	0,00000	3,26991	0,00000	0,00000	0,93555	0,72074	1,74559	0,00000	0,91107
LRRC14	0,00000	0,00000	0,00000	1,18505	0,00000	0,00000	0,11153	0,00000	0,00000	0,00000
MAGEH1	0,00000	0,00000	0,00000	0,00000	0,00000	0,00000	0,00000	0,00000	0,00000	0,00000
MESDC1	0,00000	0,00000	0,00000	0,00000	0,00000	0,00000	0,00000	0,00000	0,00000	0,00000
NANOG	0,00000	0,00000	0,97174	0,00000	0,00000	0,00000	0,00000	0,00000	0,00000	0,00000
NRBP2	0,00000	0,00000	0,00000	0,00000	0,00000	0,00000	0,00000	0,00000	0,00000	0,00000
OVOL2	0,00000	0,00000	0,93555	0,00000	0,00000	0,00000	1,14474	0,00000	0,00000	0,00000
PCDH10	0,00000	0,00000	0,00000	0,00000	0,00000	0,00000	0,00000	0,00000	0,00000	0,00000
PTGDR	0,00000	0,00000	0,47210	0,00000	0,00000	0,00000	0,00000	0,00000	0,00000	0,00000
SH2D3A	0,89320	0,00000	0,00000	0,00000	0,00000	0,00000	0,00000	0,00000	0,00000	0,00000
SLC2A5	0,00000	0,00000	0,00000	0,00000	0,00000	0,00000	0,00000	0,00000	0,00000	0,00000

Supplementary Table S6 (continued). Subset of twenty genes analysed for studying the major embryonic genome activation (EGA). The genes and their normalised log2(UMI+1) values are reported.

Cell_Name	E16.8_p3	E16.9_p3	E11.1_p3	E11.2_p3	E11.3_p3	E11.4_p3	E11.5_p3	E11.7_p3	E11.8_p3	E14.1_p2
Embryo_Name	Day2-E3	Day2-E3	Day2-E4	Day2-E4	Day2-E4	Day2-E4	Day2-E4	Day2-E4	Day2-E4	Day2-E5
ADAMTS1	0,00000	0,00000	0,00000	0,00000	0,00000	0,00000	0,00000	0,00000	0,00000	0,00000
C3	0,00000	0,67110	3,50328	0,00000	0,00000	0,93555	0,00000	0,00000	0,00000	0,00000
CNR2	0,00000	0,00000	0,00000	0,00000	0,00000	0,00000	0,00000	0,00000	0,00000	0,00000
CRABP1	0,00000	0,00000	0,00000	0,00000	0,00000	0,00000	1,18505	0,00000	0,00000	0,00000
DDX3Y	2,84016	0,74219	3,44824	2,69165	3,31987	4,43327	2,27331	0,00000	2,74154	5,20949
DPPA4	0,00000	0,00000	0,00000	0,00000	0,00000	0,00000	0,00000	0,00000	0,00000	0,00000
FOLR1	0,00000	0,00000	0,77318	0,00000	0,00000	2,07905	0,69917	0,00000	0,00000	2,56260
GAT	0,00000	0,00000	0,00000	0,00000	0,00000	0,00000	0,00000	0,00000	0,00000	0,00000
GFRA3	0,00000	0,00000	0,00000	0,00000	0,00000	0,00000	0,00000	0,00000	0,00000	0,00000
LOC508834	0,00000	0,00000	0,00000	0,00000	0,00000	0,00000	0,00000	0,00000	0,00000	0,00000
LRRC14	0,00000	0,00000	0,00000	0,00000	0,00000	0,00000	0,00000	0,00000	0,00000	0,00000
MAGEH1	0,00000	0,00000	0,00000	0,00000	0,00000	0,00000	0,00000	0,00000	0,00000	0,00000
MESDC1	0,00000	0,00000	0,00000	0,00000	0,00000	0,00000	0,00000	0,00000	0,00000	0,00000
NANOG	0,00000	0,00000	0,00000	0,00000	0,00000	0,00000	0,00000	0,00000	0,00000	0,29132
NRBP2	0,00000	0,00000	0,00000	0,00000	0,00000	0,00000	0,00000	0,00000	0,00000	0,00000
OVOL2	0,00000	0,00000	0,00000	0,00000	0,00000	0,00000	0,73522	0,00000	0,28137	2,20967
PCDH10	0,00000	0,00000	0,00000	0,00000	0,00000	0,00000	0,00000	0,00000	0,00000	0,00000
PTGDR	0,00000	0,00000	0,00000	2,97724	0,00000	0,00000	0,00000	0,00000	1,60245	0,00000
SH2D3A	0,00000	0,94392	0,00000	0,00000	0,00000	0,00000	0,00000	0,00000	0,00000	0,00000
SLC2A5	0,00000	0,00000	0,00000	0,00000	0,00000	0,00000	0,00000	0,00000	0,00000	0,00000

Cell_Name	E14.2_p2	E14.3_p2	E14.4_p2	E14.5_p2	E14.6_p2	E14.7_p2	E14.8_p2	E15.1_p2	E15.2_p2	E15.3_p2
Embryo_Name	Day2-E5	Day2-E5	Day2-E5	Day2-E5	Day2-E5	Day2-E5	Day2-E5	Day2-E6	Day2-E6	Day2-E6
ADAMTS1	0,00000	0,00000	0,00000	0,00000	0,00000	0,00000	0,00000	0,00000	0,00000	0,00000
C3	0,00000	0,00000	0,00000	0,00000	0,58228	0,80975	0,00000	0,00000	0,00000	0,00000
CNR2	0,00000	0,00000	0,00000	0,00000	0,00000	0,00000	0,00000	0,00000	0,00000	0,00000
CRABP1	1,29530	0,00000	0,00000	0,00000	0,00000	2,99000	0,00000	0,00000	0,00000	0,00000
DDX3Y	3,55433	0,85846	2,63397	1,14829	2,39224	1,81670	0,00000	0,00000	7,18738	3,09041
DPPA4	1,41544	0,00000	0,00000	0,00000	0,67639	0,00000	0,00000	0,00000	0,00000	0,92678
FOLR1	1,84471	0,47210	0,00000	0,97550	2,04848	0,33506	0,00000	4,74319	0,00000	0,00000
GAT	0,00000	0,00000	0,00000	0,00000	0,00000	0,00000	0,00000	0,00000	0,00000	0,00000
GFRA3	0,00000	0,00000	0,00000	0,00000	0,00000	0,00000	0,00000	0,00000	0,00000	0,00000
LOC508834	0,00000	0,00000	0,00000	0,00000	0,00000	0,00000	0,00000	0,00000	0,00000	0,00000
LRRC14	0,00000	0,00000	0,00000	0,00000	0,00000	0,00000	0,00000	0,00000	0,94392	0,00000
MAGEH1	0,00000	0,00000	0,00000	0,00000	0,00000	0,00000	0,00000	0,00000	0,00000	0,00000
MESDC1	0,67639	0,00000	0,00000	0,00000	0,00000	0,00000	0,00000	0,00000	0,00000	0,00000
NANOG	1,19126	0,00000	0,00000	0,00000	3,32379	0,00000	0,00000	0,00000	0,00000	0,00000
NRBP2	0,00000	0,00000	0,00000	0,00000	0,00000	0,00000	0,00000	0,00000	0,00000	0,00000
OVOL2	0,00000	0,00000	0,30887	0,00000	0,00000	0,00000	0,00000	1,19126	1,21841	1,49391
PCDH10	0,00000	0,00000	0,00000	0,00000	0,00000	0,00000	0,00000	0,00000	0,00000	0,00000
PTGDR	0,00000	0,00000	0,00000	0,00000	0,00000	0,00000	0,00000	0,00000	0,00000	0,00000
SH2D3A	0,00000	0,00000	0,00000	0,00000	0,00000	1,87173	0,00000	0,00000	0,00000	0,00000
SLC2A5	0,00000	0,00000	0,00000	0,00000	0,00000	0,00000	0,00000	0,00000	0,00000	0,00000

Supplementary Table S6 (continued). Subset of twenty genes analysed for studying the major embryonic genome activation (EGA). The genes and their normalised log2(UMI+1) values are reported.

Cell_Name	E15.4_p2	E15.5_p2	E15.6_p2	E2.1_p1	E2.2_p1	E2.3_p1	E2.4_p1	E2.5_p1	E2.6_p1	E2.7_p1
Embryo_Name	Day2-E6	Day2-E6	Day2-E6	Day3-E1	Day3-E1	Day3-E1	Day3-E1	Day3-E1	Day3-E1	Day3-E1
ADAMTS1	0,00000	0,00000	0,00000	0,00000	0,00000	0,00000	0,00000	0,00000	0,00000	0,00000
C3	0,00000	0,00000	1,14311	0,00000	0,00000	0,00000	0,00000	0,00000	0,00000	0,29132
CNR2	0,00000	0,00000	0,00000	0,58472	0,00000	0,00000	0,00000	0,00000	0,00000	0,00000
CRABP1	0,00000	0,00000	0,32407	0,00000	0,00000	0,00000	0,00000	0,00000	0,00000	1,05318
DDX3Y	0,00000	1,90222	5,00928	1,05878	1,29880	1,14474	1,94810	1,58924	0,00000	2,24148
DPPA4	0,00000	0,00000	0,00000	0,00000	0,00000	0,00000	0,00000	0,00000	0,00000	0,00000
FOLR1	0,00000	1,87173	2,97724	0,00000	0,34869	0,00000	3,69344	0,00000	0,00000	3,48211
GAT	0,00000	0,00000	0,00000	0,00000	0,00000	0,00000	0,00000	0,00000	0,00000	0,00000
GFRA3	0,00000	0,00000	0,00000	0,00000	0,00000	0,00000	0,00000	0,00000	0,00000	0,00000
LOC508834	0,00000	0,00000	0,00000	2,07296	0,00000	0,00000	0,00000	4,03585	0,00000	0,00000
LRRC14	0,00000	0,00000	0,00000	0,00000	0,00000	0,00000	0,00000	0,00000	0,00000	0,00000
MAGEH1	0,00000	0,00000	0,00000	0,00000	0,00000	0,00000	0,00000	0,00000	0,00000	0,00000
MESDC1	0,00000	0,00000	0,00000	0,00000	0,00000	0,32407	0,00000	0,00000	0,00000	0,00000
NANOG	0,00000	0,00000	0,00000	0,00000	0,00000	0,00000	0,00000	0,00000	0,00000	0,00000
NRBP2	0,00000	0,00000	0,00000	1,77948	0,00000	0,00000	0,00000	0,00000	0,00000	0,00000
OVOL2	0,00000	0,00000	0,85679	0,00000	0,00000	0,00000	1,38160	0,00000	0,00000	0,00000
PCDH10	0,00000	0,00000	0,00000	0,00000	0,00000	0,00000	0,00000	0,00000	0,00000	0,00000
PTGDR	0,00000	0,00000	0,00000	0,00000	0,00000	0,00000	0,00000	0,00000	0,00000	0,00000
SH2D3A	0,00000	0,00000	0,00000	3,48211	0,00000	0,00000	0,00000	0,00000	1,26432	3,26991
SLC2A5	0,00000	0,00000	0,00000	0,00000	0,00000	0,00000	0,00000	0,00000	0,00000	0,00000

Cell_Name	E2.9_p1	E2.10_p1	E2.11_p1	E2.12_p1	E2.13_p1	E2.14_p1	E2.15_p1	E3.1_p1	E3.2_p1	E3.3_p1
Embryo_Name	Day3-E1	Day3-E1	Day3-E1	Day3-E1	Day3-E1	Day3-E1	Day3-E1	Day3-E2	Day3-E2	Day3-E2
ADAMTS1	0,00000	0,00000	0,00000	0,00000	0,00000	0,00000	0,00000	0,00000	0,00000	0,00000
C3	1,19126	1,21841	0,00000	0,00000	0,00000	0,00000	0,00000	0,00000	0,00000	0,00000
CNR2	1,46071	0,00000	0,00000	0,00000	0,00000	0,00000	0,00000	0,00000	1,21841	0,00000
CRABP1	0,00000	0,00000	1,26019	0,00000	0,00000	0,00000	0,00000	0,00000	1,03250	0,00000
DDX3Y	2,57825	0,80975	0,99964	0,00000	3,12006	1,21990	2,21349	0,71900	3,95445	1,46071
DPPA4	2,67432	0,00000	0,00000	0,00000	0,00000	1,50359	0,00000	0,27245	2,23112	0,00000
FOLR1	4,40102	0,00000	0,00000	0,00000	0,00000	3,26991	0,00000	0,00000	2,69505	0,00000
GAT	0,00000	0,00000	0,00000	0,00000	0,00000	0,00000	0,00000	0,00000	0,00000	0,00000
GFRA3	0,00000	0,00000	0,00000	0,00000	0,00000	0,00000	0,00000	0,00000	0,00000	0,00000
LOC508834	1,24192	3,31987	0,00000	0,00000	0,76627	0,00000	1,04184	0,00000	0,00000	0,00000
LRRC14	0,00000	0,00000	0,00000	0,00000	0,00000	0,00000	0,00000	4,63658	0,00000	0,00000
MAGEH1	0,00000	0,00000	0,00000	0,00000	0,00000	0,00000	0,00000	0,00000	0,00000	0,00000
MESDC1	0,00000	0,00000	0,00000	0,00000	0,00000	0,00000	0,00000	0,89835	0,00000	0,00000
NANOG	0,00000	0,46716	0,00000	0,00000	0,00000	0,00000	0,00000	0,00000	0,00000	0,00000
NRBP2	0,00000	0,00000	0,00000	0,00000	0,00000	0,00000	0,00000	0,00000	0,00000	0,00000
OVOL2	0,00000	0,00000	0,00000	0,00000	0,00000	0,00000	0,00000	1,08502	0,00000	0,00000
PCDH10	0,00000	0,00000	0,00000	0,00000	0,00000	0,00000	0,00000	1,20672	1,54772	0,00000
PTGDR	0,00000	0,00000	0,00000	0,00000	0,00000	0,00000	0,00000	0,00000	0,00000	0,00000
SH2D3A	0,00000	0,00000	0,00000	0,00000	0,00000	0,00000	0,00000	1,80193	0,00000	0,00000
SLC2A5	0,00000	0,00000	0,00000	0,00000	0,00000	0,00000	0,00000	0,00000	0,00000	0,00000

Supplementary Table S6 (continued). Subset of twenty genes analysed for studying the major embryonic genome activation (EGA). The genes and their normalised log2(UMI+1) values are reported.

Cell_Name	E3.5_p1	E3.6_p1	E3.7_p1	E3.8_p1	E3.9_p1	E3.10_p1	E3.11_p1	E3.12_p1	E3.13_p1	E3.14_p1
Embryo_Name	Day3-E2	Day3-E2	Day3-E2	Day3-E2	Day3-E2	Day3-E2	Day3-E2	Day3-E2	Day3-E2	Day3-E2
ADAMTS1	0,00000	1,82677	0,00000	0,00000	0,00000	0,00000	1,95350	0,00000	0,00000	0,00000
C3	0,00000	0,96838	0,00000	0,92325	0,78762	0,00000	1,77968	0,00000	0,95255	0,00000
CNR2	0,00000	3,32379	0,00000	0,00000	0,00000	0,00000	1,38160	0,00000	1,54265	0,00000
CRABP1	2,19744	6,12416	0,00000	4,18915	0,00000	0,00000	2,97724	0,66911	1,06599	0,00000
DDX3Y	1,93530	2,40524	3,08684	1,88401	0,00000	1,79134	3,31971	1,86940	3,95718	1,03023
DPPA4	0,00000	3,88976	0,00000	1,76252	0,97550	0,00000	0,00000	2,80277	4,74319	0,00000
FOLR1	1,74559	7,97417	2,17689	5,05871	2,94169	5,04581	0,00000	2,07600	2,54629	3,89069
GAT	0,00000	0,00000	0,00000	0,00000	0,00000	0,00000	0,00000	0,00000	0,00000	0,00000
GFRA3	0,00000	0,00000	0,00000	0,00000	0,00000	0,20664	0,00000	0,00000	0,00000	0,00000
LOC508834	0,00000	0,00000	0,00000	0,00000	0,00000	0,00000	0,00000	0,00000	0,00000	0,00000
LRRC14	0,00000	0,00000	0,00000	0,00000	0,00000	0,00000	0,00000	0,00000	0,00000	0,00000
MAGEH1	0,00000	0,00000	0,00000	0,00000	0,00000	2,11597	0,00000	0,00000	0,00000	0,00000
MESDC1	0,97550	0,00000	0,00000	2,19744	0,00000	0,00000	2,75428	1,03674	1,87173	0,00000
NANOG	0,21505	1,29530	0,00000	0,00000	0,00000	0,00000	0,00000	0,00000	0,48920	0,00000
NRBP2	0,00000	0,00000	0,00000	0,00000	0,00000	1,21990	0,00000	0,00000	0,00000	0,00000
OVOL2	2,60873	2,93915	0,00000	1,28098	0,00000	0,67639	0,81481	0,00000	1,29530	1,28485
PCDH10	1,14578	0,28137	1,14311	2,17148	0,00000	0,00000	2,58438	0,00000	0,00000	0,53358
PTGDR	0,00000	0,00000	0,00000	0,00000	0,32453	0,00000	0,00000	0,00000	0,00000	0,00000
SH2D3A	0,00000	0,00000	0,00000	0,00000	0,00000	0,00000	0,00000	0,00000	0,00000	0,00000
SLC2A5	0,00000	0,00000	0,00000	0,00000	0,00000	0,00000	0,00000	0,00000	0,00000	0,00000

Cell_Name	E3.16_p1	E10.1_p1	E10.2_p1	E10.3_p1	E10.4_p1	E10.5_p1	E10.6_p1	E10.7_p1	E10.8_p1	E10.9_p1
Embryo_Name	Day3-E2	Day3-E3	Day3-E3	Day3-E3	Day3-E3	Day3-E3	Day3-E3	Day3-E3	Day3-E3	Day3-E3
ADAMTS1	0,00000	1,29913	0,00000	0,00000	0,00000	0,89320	0,00000	0,32407	0,00000	2,70686
C3	0,00000	0,46716	0,00000	0,00000	0,00000	0,00000	1,94864	0,00000	1,13506	0,00000
CNR2	0,92325	0,00000	0,00000	0,00000	0,00000	0,00000	0,00000	0,00000	0,00000	0,89044
CRABP1	0,00000	0,00000	0,00000	0,00000	0,00000	0,00000	0,24595	0,00000	0,00000	0,00000
DDX3Y	7,18196	2,33772	0,00000	0,62097	1,32446	1,38160	3,51824	2,27100	1,78872	2,19709
DPPA4	0,00000	0,00000	0,00000	0,00000	0,00000	0,00000	0,00000	0,00000	0,00000	0,00000
FOLR1	1,78888	0,00000	1,74045	1,75626	1,25456	0,00000	0,00000	1,83990	0,77764	0,17612
GAT	0,00000	0,00000	0,00000	0,00000	0,00000	0,00000	0,00000	0,00000	0,00000	0,00000
GFRA3	0,00000	0,00000	0,00000	0,00000	0,00000	0,00000	0,00000	0,00000	0,00000	0,00000
LOC508834	0,00000	0,79088	0,00000	0,00000	0,00000	0,00000	1,16539	0,58228	0,00000	0,00000
LRRC14	0,00000	0,00000	0,00000	0,00000	0,00000	0,00000	0,00000	0,00000	0,00000	0,00000
MAGEH1	0,00000	0,00000	0,00000	0,00000	0,00000	0,00000	0,00000	0,00000	0,00000	0,00000
MESDC1	0,66911	0,00000	0,00000	0,00000	0,00000	0,00000	0,00000	0,00000	1,39891	0,00000
NANOG	0,00000	0,00000	0,00000	0,00000	0,00000	0,00000	0,00000	0,77318	1,87314	0,00000
NRBP2	0,00000	0,00000	0,00000	0,00000	0,00000	0,00000	0,00000	0,00000	0,00000	0,00000
OVOL2	0,92678	0,00000	0,00000	0,00000	1,29913	0,00000	0,00000	0,00000	0,89320	0,00000
PCDH10	0,00000	0,00000	0,00000	0,00000	3,32379	0,00000	0,00000	0,00000	0,00000	0,00000
PTGDR	0,00000	0,00000	0,00000	2,10601	0,00000	0,00000	0,00000	0,00000	0,76627	0,00000
SH2D3A	0,00000	0,00000	0,00000	0,33307	0,33919	0,00000	0,00000	0,00000	0,00000	0,00000
SLC2A5	0,00000	0,00000	0,00000	0,00000	0,00000	0,00000	0,00000	0,00000	0,00000	0,00000

Supplementary Table S6 (continued). Subset of twenty genes analysed for studying the major embryonic genome activation (EGA). The genes and their normalised log2(UMI+1) values are reported.

Cell_Name	E10.10_p1	E10.11_p1	E10.12_p1	E10.13_p1	E10.14_p1	E10.15_p1	E10.16_p1	E16.1_p1	E16.2_p1	E16.3_p1
Embryo_Name	Day3-E3	Day3-E3	Day3-E3	Day3-E3	Day3-E3	Day3-E3	Day3-E3	Day3-E4	Day3-E4	Day3-E4
ADAMTS1	2,36361	0,00000	0,00000	0,00000	0,00000	0,00000	0,00000	0,00000	1,78821	1,84471
C3	0,11153	0,00000	1,95350	0,00000	0,25927	0,00000	0,00000	0,00000	0,00000	1,47223
CNR2	0,00000	0,00000	0,24529	0,00000	0,00000	0,00000	0,00000	0,00000	0,00000	0,00000
CRABP1	0,00000	1,70985	0,00000	0,00000	0,00000	0,00000	0,00000	0,00000	0,96697	0,00000
DDX3Y	3,48118	0,92240	2,55263	2,33103	1,92823	3,28240	0,00000	1,87840	0,00000	2,96339
DPPA4	0,00000	0,00000	0,00000	0,00000	0,00000	0,00000	0,00000	0,00000	0,00000	0,00000
FOLR1	1,75934	2,68350	0,73619	0,81293	0,00000	1,27750	2,07296	4,21560	2,07310	5,61349
GAT	0,00000	0,00000	0,00000	0,00000	0,00000	0,00000	0,00000	0,00000	0,00000	0,00000
GFRA3	0,00000	0,00000	0,74219	0,00000	0,00000	0,00000	0,00000	0,00000	0,00000	0,00000
LOC508834	0,00000	0,00000	0,73522	0,00000	0,00000	0,00000	0,00000	0,00000	0,00000	0,00000
LRRC14	0,00000	0,00000	0,00000	0,00000	0,00000	0,00000	0,00000	0,00000	0,00000	0,00000
MAGEH1	0,00000	0,00000	0,00000	0,00000	0,00000	0,00000	0,00000	1,25229	0,00000	0,00000
MESDC1	0,00000	1,63376	0,00000	0,00000	0,00000	0,00000	0,00000	0,00000	0,00000	0,00000
NANOG	1,65544	0,69917	0,00000	0,00000	0,00000	0,00000	0,47210	0,00000	0,97550	1,62813
NRBP2	0,00000	0,00000	0,00000	0,00000	0,00000	0,00000	0,00000	0,00000	0,00000	0,00000
OVOL2	0,32407	0,00000	0,00000	0,00000	0,77318	2,32047	0,00000	0,00000	0,00000	0,00000
PCDH10	0,00000	0,00000	0,49486	0,00000	0,00000	0,00000	0,00000	0,00000	0,00000	0,00000
PTGDR	0,00000	0,00000	0,00000	0,00000	0,00000	0,00000	0,00000	2,21397	0,00000	0,00000
SH2D3A	0,00000	0,00000	1,38070	0,00000	0,00000	0,00000	0,00000	0,00000	0,00000	1,30554
SLC2A5	0,00000	0,00000	0,00000	0,00000	0,00000	0,00000	0,00000	0,00000	0,00000	0,77318

Cell_Name	E16.4_p1	E16.5_p1	E16.6_p1	E16.7_p1	E16.8_p1	E16.9_p1	E16.10_p1	E16.11_p1	E16.12_p1	E16.13_p1
Embryo_Name	Day3-E4	Day3-E4	Day3-E4	Day3-E4	Day3-E4	Day3-E4	Day3-E4	Day3-E4	Day3-E4	Day3-E4
ADAMTS1	0,82721	2,19829	1,96309	1,62813	0,33506	0,00000	6,12416	0,00000	5,72040	0,00000
C3	1,00000	0,00000	1,14362	0,00000	2,67432	0,00000	1,91241	2,18432	0,00000	0,00000
CNR2	0,00000	0,00000	0,00000	0,00000	0,00000	0,48920	0,00000	0,00000	0,89320	0,00000
CRABP1	5,61496	0,00000	0,00000	0,00000	0,00000	0,00000	1,11816	0,00000	0,94408	1,46118
DDX3Y	4,60547	1,31186	4,29209	4,02543	2,19813	0,46418	0,71631	4,08722	4,89392	0,00000
DPPA4	1,84483	0,00000	0,00000	0,00000	0,00000	0,00000	4,39371	3,40622	0,85127	0,00000
FOLR1	7,60786	3,18151	6,86635	5,13456	4,70930	5,29520	4,73822	3,00155	5,44820	2,87835
GAT	0,00000	0,00000	0,00000	0,00000	0,00000	0,28137	0,00000	1,46071	0,00000	0,00000
GFRA3	0,00000	0,00000	0,00000	0,00000	0,00000	0,00000	0,50636	2,21397	0,67110	0,00000
LOC508834	0,00000	0,00000	0,00000	0,00000	0,00000	0,00000	0,00000	0,00000	0,00000	0,00000
LRRC14	0,17612	0,00000	1,05992	0,00000	0,00000	1,53135	1,27750	0,00000	0,00000	0,00000
MAGEH1	1,50359	1,65544	0,00000	0,00000	0,00000	0,00000	2,15566	1,11195	2,53252	0,00000
MESDC1	2,57863	0,96697	0,00000	0,00000	0,85127	0,00000	3,89069	0,00000	2,46661	0,00000
NANOG	1,50080	2,80277	6,12416	1,49776	4,18915	4,06814	4,29410	5,80731	1,12446	0,00000
NRBP2	0,00000	0,00000	0,00000	0,00000	0,00000	0,00000	0,00000	0,81947	0,89044	0,00000
OVOL2	1,26019	0,00000	2,81712	0,00000	1,13517	0,00000	1,03250	0,00000	0,00000	0,00000
PCDH10	1,69725	0,00000	0,00000	0,00000	0,00000	0,24529	0,00000	2,60759	1,13506	0,00000
PTGDR	1,05235	0,00000	0,93555	0,00000	0,00000	0,79088	0,00000	0,00000	1,00321	0,00000
SH2D3A	0,00000	0,00000	1,27617	0,00000	0,92270	0,00000	1,84946	1,14829	0,55796	0,00000
SLC2A5	0,00000	0,00000	0,00000	0,27245	0,00000	0,00000	1,15505	0,00000	1,11195	0,00000

Supplementary Table S6 (continued). Subset of twenty genes analysed for studying the major embryonic genome activation (EGA). The genes and their normalised log2(UMI+1) values are reported.

Cell_Name	E6.1_p3	E6.2_p3	E6.3_p3	E6.4_p3	E6.5_p3	E6.6_p3	E6.7_p3	E6.8_p3	E6.9_p3	E6.10_p3
Embryo_Name	Day3-E5	Day3-E5	Day3-E5	Day3-E5	Day3-E5	Day3-E5	Day3-E5	Day3-E5	Day3-E5	Day3-E5
ADAMTS1	3,16657	2,97724	0,00000	0,00000	0,00000	3,69344	1,60245	0,16610	3,48211	0,00000
C3	1,05318	0,27245	0,00000	0,00000	0,00000	0,00000	0,00000	0,00000	0,00000	0,00000
CNR2	0,00000	0,00000	1,25233	0,00000	0,00000	0,00000	0,00000	0,00000	0,00000	0,00000
CRABP1	0,00000	0,00000	0,00000	0,00000	0,00000	0,00000	0,00000	0,00000	0,00000	0,00000
DDX3Y	0,00000	1,24762	0,00000	0,00000	1,14471	2,18556	0,00000	1,83668	1,49264	1,00535
DPPA4	0,00000	0,00000	0,00000	0,00000	0,00000	0,00000	0,00000	0,00000	0,00000	0,00000
FOLR1	0,53389	2,34071	0,00000	2,21709	0,00000	1,97937	4,26284	0,67110	4,43822	0,62007
GAT	0,00000	0,00000	0,00000	0,00000	0,00000	0,00000	0,00000	0,00000	0,00000	0,00000
GFRA3	0,00000	1,46194	0,00000	2,44839	0,00000	0,00000	0,00000	0,00000	0,00000	0,00000
LOC508834	0,62097	0,67965	0,00000	0,00000	0,00000	0,49486	0,00000	1,29422	0,00000	0,00000
LRRC14	0,00000	0,00000	0,00000	0,00000	0,00000	0,00000	0,00000	0,00000	0,00000	0,00000
MAGEH1	0,00000	0,00000	0,00000	0,00000	0,00000	0,00000	0,00000	0,00000	0,00000	0,00000
MESDC1	0,00000	0,00000	0,00000	0,00000	1,19530	0,00000	0,00000	0,00000	0,00000	0,00000
NANOG	0,00000	0,00000	0,00000	0,45003	0,00000	0,00000	0,00000	0,00000	0,00000	0,00000
NRBP2	0,00000	0,00000	0,00000	0,00000	0,00000	0,00000	0,00000	0,00000	0,00000	0,00000
OVOL2	0,00000	0,00000	0,00000	0,00000	0,00000	0,00000	0,00000	2,02175	0,00000	0,00000
PCDH10	0,00000	0,00000	0,00000	0,00000	0,25927	0,00000	0,00000	0,00000	0,00000	0,00000
PTGDR	0,00000	0,00000	0,00000	0,00000	0,00000	0,00000	0,00000	0,00000	0,00000	0,00000
SH2D3A	0,00000	0,00000	3,50328	0,00000	0,00000	0,00000	0,00000	0,00000	0,00000	0,00000
SLC2A5	0,00000	0,00000	0,00000	0,00000	0,00000	1,49776	0,00000	0,00000	0,00000	0,00000

Cell_Name	E6.11_p3	E6.12_p3	E6.13_p3	E6.14_p3	E6.15_p3	E6.16_p3	E6.17_p3	E13.1_p3	E13.2_p3	E13.3_p3
Embryo_Name	Day3-E5	Day3-E5	Day3-E5	Day3-E5	Day3-E5	Day3-E5	Day3-E5	Day3-E6	Day3-E6	Day3-E6
ADAMTS1	0,00000	0,80198	0,38788	1,26432	0,00000	0,00000	0,00000	2,47590	0,72074	2,51264
C3	0,00000	0,00000	0,00000	0,00000	0,00000	1,03674	0,00000	0,00000	1,12446	0,34869
CNR2	0,00000	0,00000	0,00000	0,00000	0,00000	0,00000	0,00000	0,00000	0,00000	0,00000
CRABP1	0,00000	0,60125	0,00000	0,00000	0,00000	0,00000	0,00000	0,00000	0,00000	0,00000
DDX3Y	0,00000	1,25233	0,00000	1,64482	2,32047	2,22372	2,07905	1,16809	1,50969	1,92241
DPPA4	0,00000	0,00000	0,00000	0,00000	0,00000	0,00000	0,00000	1,64999	0,00000	0,00000
FOLR1	0,00000	1,49838	0,00000	0,62442	1,97104	3,04725	1,53892	2,32706	2,12571	4,69259
GAT	0,00000	0,00000	0,00000	0,00000	0,00000	0,00000	0,00000	0,00000	0,00000	0,92240
GFRA3	0,00000	0,00000	0,00000	0,00000	0,00000	0,00000	0,00000	0,00000	0,28137	0,00000
LOC508834	0,00000	0,00000	0,00000	1,08502	0,00000	0,89044	0,00000	1,41544	0,00000	0,00000
LRRC14	0,00000	0,00000	0,00000	0,00000	0,00000	0,00000	0,00000	0,00000	0,00000	0,00000
MAGEH1	0,00000	0,00000	3,69344	0,00000	0,00000	3,48211	0,00000	0,00000	0,00000	0,00000
MESDC1	0,00000	0,00000	0,00000	0,00000	0,00000	0,00000	0,00000	0,00000	0,00000	0,00000
NANOG	0,00000	0,00000	0,00000	0,93555	0,00000	0,00000	0,00000	0,91107	0,00000	1,54075
NRBP2	0,00000	0,00000	0,00000	0,00000	0,00000	0,00000	0,00000	0,00000	0,00000	0,00000
OVOL2	0,00000	0,00000	0,00000	0,00000	0,00000	0,00000	0,00000	0,00000	0,00000	0,00000
PCDH10	0,00000	0,00000	0,00000	0,00000	0,00000	0,00000	0,00000	0,00000	0,00000	0,00000
PTGDR	0,00000	0,00000	0,00000	0,00000	0,00000	0,00000	0,00000	0,00000	0,00000	0,00000
SH2D3A	0,00000	0,00000	0,00000	0,00000	0,00000	0,00000	0,00000	0,00000	0,00000	0,00000
SLC2A5	0,00000	0,00000	0,00000	0,00000	0,00000	0,00000	0,00000	0,00000	0,00000	0,00000

Supplementary Table S6 (continued). Subset of twenty genes analysed for studying the major embryonic genome activation (EGA). The genes and their normalised log2(UMI+1) values are reported.

Cell_Name	E13.4_p3	E13.5_p3	E13.6_p3	E13.7_p3	E13.8_p3	E13.9_p3	E13.10_p3	E13.11_p3	E13.12_p3	E13.13_p3
Embryo_Name	Day3-E6	Day3-E6	Day3-E6	Day3-E6	Day3-E6	Day3-E6	Day3-E6	Day3-E6	Day3-E6	Day3-E6
ADAMTS1	3,57161	0,00000	0,00000	2,52948	3,17122	4,33652	3,87475	0,00000	0,00000	3,80880
C3	0,00000	5,60728	0,00000	0,00000	0,00000	3,46774	1,39891	0,24595	0,00000	0,76644
CNR2	0,00000	0,00000	0,00000	0,00000	0,00000	0,66911	0,00000	0,00000	0,00000	0,00000
CRABP1	0,00000	0,76627	0,00000	0,00000	0,53389	0,00000	0,00000	0,00000	0,00000	0,00000
DDX3Y	3,06377	2,15566	1,73238	1,13517	2,95684	2,86263	1,74539	0,00000	5,45845	5,59767
DPPA4	0,00000	0,00000	0,00000	0,00000	1,24192	0,00000	0,00000	0,00000	0,00000	0,00000
FOLR1	1,57960	3,86318	1,32149	3,34438	4,58992	3,36528	2,82033	0,51672	2,20967	5,92083
GAT	0,00000	0,00000	0,00000	0,00000	0,00000	0,00000	0,00000	0,00000	0,00000	0,24529
GFRA3	0,00000	0,00000	0,00000	0,00000	0,92372	0,00000	0,00000	0,00000	0,00000	0,00000
LOC508834	1,18505	0,00000	0,00000	0,11153	0,00000	0,00000	0,00000	0,25927	0,00000	2,16104
LRRC14	0,00000	0,00000	0,00000	0,00000	0,00000	0,00000	0,00000	0,00000	0,00000	0,00000
MAGEH1	0,00000	0,00000	0,00000	0,00000	0,00000	0,00000	0,00000	0,00000	0,00000	1,80193
MESDC1	1,24192	0,00000	0,00000	0,00000	1,92731	0,00000	0,00000	0,00000	0,85846	2,90865
NANOG	3,70179	0,00000	1,38304	0,00000	3,89069	0,31678	2,73537	0,00000	0,00000	1,05517
NRBP2	0,00000	2,36366	0,00000	0,77318	0,00000	0,00000	1,05318	0,00000	0,94392	1,62896
OVOL2	0,00000	0,00000	0,00000	0,00000	0,00000	2,57863	0,96697	0,00000	0,00000	0,00000
PCDH10	0,00000	0,00000	0,00000	0,00000	0,00000	0,00000	0,00000	0,00000	0,00000	1,03250
PTGDR	0,00000	0,00000	0,00000	0,00000	0,00000	0,00000	1,38160	0,00000	0,00000	0,00000
SH2D3A	0,00000	0,89638	0,00000	0,00000	0,00000	0,00000	0,00000	0,92372	0,00000	1,08325
SLC2A5	0,00000	0,00000	0,00000	0,00000	0,00000	0,00000	0,00000	0,00000	0,00000	0,00000

Cell_Name	E13.14_p3	E6.1_p2	E6.2_p2	E6.3_p2	E6.4_p2	E6.6_p2	E6.7_p2	E6.8_p2	E6.9_p2	E6.10_p2
Embryo_Name	Day3-E6	Day3-E7	Day3-E7	Day3-E7	Day3-E7	Day3-E7	Day3-E7	Day3-E7	Day3-E7	Day3-E7
ADAMTS1	0,00000	2,77684	0,00000	0,00000	3,00241	2,55554	1,19530	0,00000	0,47460	0,61362
C3	0,00000	1,16643	0,00000	0,93555	0,00000	2,51264	4,50962	0,00000	0,00000	1,54075
CNR2	0,00000	4,41607	0,00000	0,00000	0,45602	0,69326	0,00000	0,00000	0,69802	0,00000
CRABP1	0,00000	0,00000	0,00000	0,62007	0,00000	0,00000	0,00000	0,00000	0,79088	0,00000
DDX3Y	0,00000	2,64569	0,00000	0,00000	1,98203	1,24307	2,83219	0,00000	3,48323	0,45003
DPPA4	0,00000	0,92270	0,00000	0,00000	0,00000	0,00000	0,00000	0,00000	0,00000	0,00000
FOLR1	2,25281	1,65472	1,80685	3,44188	4,59336	1,08325	1,83473	1,86940	3,57278	0,70081
GAT	0,00000	0,00000	0,00000	0,46418	0,00000	0,00000	0,00000	0,00000	0,00000	0,00000
GFRA3	0,00000	0,00000	0,00000	0,00000	0,00000	0,20218	0,00000	0,00000	0,00000	0,00000
LOC508834	0,00000	0,85387	0,00000	0,00000	0,00000	0,00000	0,00000	4,10014	1,91246	1,25229
LRRC14	0,00000	0,00000	0,00000	0,00000	0,00000	0,00000	0,00000	0,00000	0,00000	0,00000
MAGEH1	0,00000	0,00000	0,00000	0,00000	0,00000	0,00000	0,00000	0,00000	1,11816	0,00000
MESDC1	1,77948	0,55796	0,00000	2,21397	1,12736	0,00000	0,00000	0,00000	0,00000	3,30978
NANOG	0,00000	3,42731	0,00000	0,00000	0,17612	0,33919	0,00000	0,73619	1,59363	2,25735
NRBP2	0,00000	0,00000	0,00000	0,00000	0,00000	1,03250	0,00000	0,00000	3,26213	0,00000
OVOL2	0,00000	3,89069	0,31678	1,09211	0,00000	0,00000	0,00000	0,00000	0,00000	0,00000
PCDH10	0,00000	0,00000	0,00000	0,00000	2,75428	0,00000	0,00000	0,00000	0,00000	0,00000
PTGDR	0,00000	0,00000	0,00000	0,00000	0,00000	0,95255	0,00000	1,08502	0,00000	0,00000
SH2D3A	1,19126	0,00000	0,00000	0,00000	3,32379	0,00000	0,00000	0,00000	0,00000	0,00000
SLC2A5	0,00000	0,00000	0,00000	4,33652	0,00000	0,00000	0,00000	0,00000	0,00000	0,00000

Supplementary Table S6 (continued). Subset of twenty genes analysed for studying the major embryonic genome activation (EGA). The genes and their normalised log2(UMI+1) values are reported.

Cell_Name	E6.11_p2	E6.12_p2	E6.13_p2	E6.14_p2	E6.15_p2	E6.16_p2	E7.1_p2	E7.2_p2	E7.3_p2	E7.5_p2
Embryo_Name	Day3-E7	Day3-E7	Day3-E7	Day3-E7	Day3-E7	Day3-E7	Day3-E8	Day3-E8	Day3-E8	Day3-E8
ADAMTS1	1,66432	0,00000	0,00000	0,00000	1,27750	0,00000	1,61735	0,00000	1,71534	0,00000
C3	0,00000	3,40622	0,85127	0,00000	0,00000	0,98709	0,00000	0,00000	0,00000	0,00000
CNR2	0,00000	0,00000	0,00000	0,00000	0,00000	0,00000	1,95017	2,32310	4,33652	0,00000
CRABP1	0,00000	1,00321	0,00000	0,00000	0,58228	1,32528	0,00000	1,85449	1,54772	0,00000
DDX3Y	7,05816	6,15876	2,09560	0,94813	2,56935	1,92762	5,94301	2,85256	2,47054	0,93555
DPPA4	0,62007	0,00000	0,00000	0,00000	0,00000	2,21742	2,21200	0,00000	0,00000	0,00000
FOLR1	5,21146	3,05295	2,27462	1,65611	1,32446	4,45104	5,63815	5,39697	3,32221	0,92325
GAT	0,00000	0,00000	0,00000	0,00000	0,00000	0,89320	0,00000	0,00000	0,00000	0,00000
GFRA3	0,00000	0,00000	0,00000	0,00000	0,00000	0,00000	1,16807	1,08502	0,46716	0,00000
LOC508834	1,27385	1,87314	0,00000	0,00000	0,00000	0,00000	0,00000	1,15505	0,00000	0,00000
LRRC14	0,00000	0,00000	0,00000	0,00000	0,00000	1,55009	0,00000	0,00000	0,00000	0,00000
MAGEH1	0,00000	0,00000	0,00000	0,00000	0,00000	0,00000	0,84410	0,00000	0,00000	0,00000
MESDC1	0,00000	0,00000	0,00000	0,96561	1,00321	0,00000	1,16539	0,58228	0,80975	0,00000
NANOG	0,77576	0,00000	0,00000	0,00000	2,97292	2,02875	1,59366	3,82985	1,89928	0,00000
NRBP2	0,00000	0,00000	0,00000	0,00000	0,00000	0,00000	3,34799	0,00000	0,00000	0,00000
OVOL2	0,00000	0,00000	0,61362	1,66432	0,00000	0,58930	3,83127	1,27750	0,00000	0,00000
PCDH10	0,00000	0,00000	0,00000	0,16610	3,48211	4,40102	1,39891	0,24595	0,94515	0,00000
PTGDR	0,00000	0,00000	0,00000	0,00000	1,18505	0,00000	0,00000	0,11153	0,00000	0,00000
SH2D3A	2,99490	1,54265	0,00000	1,88329	0,78762	0,00000	0,00000	1,59255	0,00000	0,00000
SLC2A5	0,00000	0,00000	0,90818	0,00000	2,89717	0,00000	0,00000	0,00000	1,05992	0,00000

Cell_Name	E7.6_p2	E7.7_p2	E7.8_p2	E7.9_p2	E7.10_p2	E7.11_p2	E7.12_p2	E7.13_p2	E7.14_p2	E7.15_p2
Embryo_Name	Day3-E8	Day3-E8	Day3-E8	Day3-E8	Day3-E8	Day3-E8	Day3-E8	Day3-E8	Day3-E8	Day3-E8
ADAMTS1	0,74219	2,10601	0,00000	1,48396	0,00000	0,00000	3,43457	0,77482	1,64048	1,69561
C3	0,90818	1,78217	1,19530	0,00000	0,00000	0,00000	0,00000	0,00000	0,00000	1,53135
CNR2	0,00000	4,84122	0,57637	2,46661	1,11816	0,00000	0,00000	1,85997	0,00000	1,83990
CRABP1	1,14578	0,51672	1,14311	0,00000	0,00000	0,30887	3,08684	0,92372	2,76236	1,08325
DDX3Y	2,28929	4,98154	8,20464	4,21791	4,20226	2,26871	5,35898	6,60016	1,38304	3,64363
DPPA4	0,00000	0,00000	2,00419	0,99964	2,29994	0,00000	1,58205	1,77623	0,00000	1,14311
FOLR1	4,14593	3,85090	5,37707	5,98847	4,99388	5,52029	4,68207	4,18643	4,42558	5,32544
GAT	1,91241	0,77318	1,87314	0,00000	0,00000	1,16809	0,94392	1,26019	1,15505	0,00000
GFRA3	0,00000	1,41544	0,24529	0,00000	1,18505	0,00000	0,00000	0,00000	0,00000	0,00000
LOC508834	1,13517	0,00000	0,00000	0,00000	0,00000	0,00000	1,49776	0,00000	0,00000	0,00000
LRRC14	0,27318	0,00000	0,85679	0,00000	0,62097	0,00000	0,00000	0,00000	0,96838	0,00000
MAGEH1	0,00000	0,00000	0,00000	0,00000	0,00000	0,00000	2,02875	0,00000	0,00000	0,00000
MESDC1	2,29994	3,64862	0,73522	0,00000	0,51672	1,14311	2,64554	1,27040	0,30887	3,45879
NANOG	1,97948	0,46825	1,26407	1,94258	0,00000	0,00000	1,39298	3,51281	1,14829	1,53045
NRBP2	0,00000	0,00000	0,00000	0,00000	0,00000	0,00000	0,00000	0,00000	1,70985	0,00000
OVOL2	2,53900	2,47703	2,02875	0,00000	0,00000	2,34920	0,00000	2,78388	1,10453	0,76627
PCDH10	0,00000	0,00000	0,00000	2,47590	0,72074	1,74559	3,57161	1,46513	0,00000	1,54075
PTGDR	0,00000	0,00000	0,00000	0,00000	1,29913	0,00000	0,00000	1,00000	0,00000	0,00000
SH2D3A	0,00000	0,00000	0,00000	0,88748	0,00000	0,00000	0,00000	0,00000	0,00000	0,00000
SLC2A5	0,00000	1,53135	1,64905	0,00000	0,00000	0,00000	1,71534	2,02875	0,00000	2,92790

Supplementary Table S6 (continued). Subset of twenty genes analysed for studying the major embryonic genome activation (EGA). The genes and their normalised log2(UMI+1) values are reported.

Cell_Name	E7.16_p2									
Embryo_Name	Day3-E8									
ADAMTS1	0,00000									
C3	1,27750									
CNR2	0,44060									
CRABP1	0,00000									
DDX3Y	5,40931									
DPPA4	1,46071									
FOLR1	5,52419									
GAT	0,47210									
GFRA3	0,00000									
LOC508834	0,00000									
LRRC14	0,00000									
MAGEH1	0,00000									
MESDC1	0,00000									
NANOG	1,17720									
NRBP2	0,00000									
OVOL2	0,00000									
PCDH10	3,17122									
PTGDR	0,00000									
SH2D3A	0,00000									
SLC2A5	0,00000									

Supplementary Table S7. Gene normalised log2(UMI+1) values.

Cell_Name	E1.1_p3	E1.2_p3	E1.3_p3	E1.5_p3	E1.6_p3	E1.7_p3	E14.1_p3	E14.2_p3	E14.3_p3	E14.4_p3
Embryo_Name	Day2-E1	Day2-E1	Day2-E1	Day2-E1	Day2-E1	Day2-E1	Day2-E2	Day2-E2	Day2-E2	Day2-E2
ADAMTS1	3,21616	0,00000	0,00000	0,00000	0,00000	1,29572	3,89299	0,00000	1,78821	2,81712
C3	1,14426	0,00000	0,00000	0,00000	0,00000	3,02408	1,05517	0,00000	1,78217	1,83990
CNR2	0,00000	0,67110	0,00000	0,00000	0,00000	0,93555	2,44839	0,00000	0,00000	1,14474
CRABP1	0,00000	0,00000	0,00000	0,00000	0,00000	0,00000	1,59255	0,00000	0,00000	0,00000
DDX3Y	3,39166	2,91468	0,00000	0,00000	1,14578	2,22061	5,31012	2,64554	2,38870	1,65472
DPPA4	7,03909	4,95991	0,53736	2,07421	2,99490	7,19387	6,90775	2,19709	6,21623	5,39856
FOLR1	1,46071	0,00000	0,00000	0,00000	0,00000	3,41166	0,00000	0,00000	1,86940	0,00000
GAT	1,41297	1,91504	0,76353	1,15505	0,89835	1,89138	4,70739	0,97550	3,37991	1,36097
GFRA3	0,00000	0,00000	0,00000	0,00000	0,00000	0,00000	0,00000	0,00000	0,00000	0,00000

Cell_Name	E14.5_p3	E14.6_p3	E14.7_p3	E14.8_p3	E14.9_p3	E16.1_p3	E16.2_p3	E16.4_p3	E16.6_p3	E16.7_p3
Embryo_Name	Day2-E2	Day2-E2	Day2-E2	Day2-E2	Day2-E2	Day2-E3	Day2-E3	Day2-E3	Day2-E3	Day2-E3
ADAMTS1	0,82721	2,53252	2,54389	2,86263	0,00000	3,22788	5,45845	2,69423	4,58428	3,52555
C3	0,77764	0,17612	0,61362	1,05992	0,00000	0,32453	3,09799	1,27750	2,39962	0,60125
CNR2	0,00000	0,00000	0,97174	0,00000	0,00000	0,00000	0,00000	0,00000	0,00000	0,00000
CRABP1	0,00000	0,00000	0,00000	0,00000	0,00000	0,00000	0,00000	0,00000	0,00000	0,00000
DDX3Y	4,39165	1,88401	4,34223	1,96676	2,27815	4,08089	4,72417	1,03023	6,77023	3,71865
DPPA4	5,93136	6,11973	6,26225	6,91555	0,00000	6,05283	6,18417	4,86038	7,05230	4,61686
FOLR1	0,27318	4,24988	0,00000	1,18876	0,00000	0,37949	0,00000	2,16539	1,54265	0,00000
GAT	2,19744	5,14470	4,51654	5,14905	0,00000	4,47753	5,40084	2,78680	1,54291	4,83526
GFRA3	0,00000	0,00000	0,00000	0,00000	0,00000	0,00000	0,00000	0,00000	0,00000	0,00000

Cell_Name	E16.8_p3	E16.9_p3	E11.1_p3	E11.2_p3	E11.3_p3	E11.4_p3	E11.5_p3	E11.7_p3	E11.8_p3	E14.1_p2
Embryo_Name	Day2-E3	Day2-E3	Day2-E4	Day2-E4	Day2-E4	Day2-E4	Day2-E4	Day2-E4	Day2-E4	Day2-E5
ADAMTS1	3,83780	4,83285	0,00000	0,62929	0,00000	5,20208	3,19320	0,00000	4,41607	6,34888
C3	0,00000	0,00000	0,00000	0,00000	2,10601	2,96821	1,48396	0,00000	0,00000	2,37994
CNR2	0,00000	0,00000	0,00000	0,00000	0,00000	0,00000	0,00000	0,00000	0,00000	0,29132
CRABP1	0,00000	0,00000	0,00000	0,00000	0,00000	0,00000	0,00000	0,00000	0,00000	0,00000
DDX3Y	3,11726	1,65611	0,80916	3,41053	3,90145	4,10960	2,22732	0,92325	1,66842	4,09717
DPPA4	6,48644	5,29320	4,04890	5,03916	1,83668	7,66637	7,07255	0,00000	2,61320	7,65590
FOLR1	0,92325	0,78762	0,00000	0,85692	0,00000	1,52134	0,00000	0,00000	0,00000	1,83237
GAT	3,69344	3,93132	0,31504	4,41607	4,40102	4,24848	1,60626	0,00000	0,76644	6,71081
GFRA3	0,00000	0,00000	0,00000	0,00000	0,00000	0,00000	0,00000	0,00000	0,00000	0,00000

Cell_Name	E14.2_p2	E14.3_p2	E14.4_p2	E14.5_p2	E14.6_p2	E14.7_p2	E14.8_p2	E15.1_p2	E15.2_p2	E15.3_p2
Embryo_Name	Day2-E5	Day2-E5	Day2-E5	Day2-E5	Day2-E5	Day2-E5	Day2-E5	Day2-E6	Day2-E6	Day2-E6
ADAMTS1	4,47776	0,63934	0,94515	1,26432	4,75155	2,03963	0,00000	0,93555	2,28929	3,67430
C3	1,64752	1,64048	0,00000	0,00000	0,00000	2,21709	0,00000	0,00000	2,21397	0,67110
CNR2	1,19126	0,00000	0,00000	0,00000	3,32379	0,00000	0,00000	0,00000	0,00000	0,00000
CRABP1	0,00000	0,00000	0,00000	0,00000	0,00000	0,00000	0,00000	0,00000	0,00000	0,00000
DDX3Y	3,96357	3,91751	2,50499	2,58218	3,96147	2,48248	0,89044	1,82775	4,79069	1,60341
DPPA4	8,54127	6,06040	4,67942	5,32334	6,83289	6,19007	1,14362	1,91473	9,31342	6,80194
FOLR1	1,43290	0,00000	0,24529	0,00000	1,18505	0,00000	0,00000	0,00000	1,96664	0,00000
GAT	1,51946	1,70985	2,21739	2,28929	4,30936	6,77997	0,91107	2,57863	3,52382	4,64526
GFRA3	0,00000	0,00000	0,00000	0,00000	0,00000	0,00000	0,00000	0,00000	0,00000	0,00000

Supplementary Table S7 (continued). Gene normalised log2(UMI+1) values.

Cell_Name	E15.4_p2	E15.5_p2	E15.6_p2	E2.1_p1	E2.2_p1	E2.3_p1	E2.4_p1	E2.5_p1	E2.6_p1	E2.7_p1
Embryo_Name	Day2-E6	Day2-E6	Day2-E6	Day3-E1	Day3-E1	Day3-E1	Day3-E1	Day3-E1	Day3-E1	Day3-E1
ADAMTS1	0,00000	1,46513	3,99287	1,95017	4,08884	4,33652	1,77073	1,14426	3,89069	0,79630
C3	0,00000	0,00000	0,00000	1,49838	0,00000	0,34573	0,00000	0,00000	0,00000	0,00000
CNR2	0,00000	0,00000	0,00000	0,00000	0,00000	0,00000	0,00000	0,00000	0,00000	0,00000
CRABP1	0,00000	0,00000	0,00000	1,78821	0,00000	0,00000	0,00000	0,00000	0,00000	0,60678
DDX3Y	0,00000	2,26937	2,67429	3,71644	0,40216	3,95818	1,95350	0,00000	1,36455	4,76104
DPPA4	1,91241	3,24919	6,45215	6,74763	5,38728	2,89599	4,84360	4,35993	4,18404	4,92475
FOLR1	0,00000	0,00000	0,00000	3,89622	0,00000	0,00000	0,00000	1,00000	0,89320	0,00000
GAT	3,40622	0,00000	0,68272	3,89069	0,57637	1,09211	2,17380	0,00000	0,00000	1,85997
GFRA3	0,00000	0,00000	0,00000	0,00000	0,00000	0,00000	0,00000	0,00000	0,00000	0,00000

Cell_Name	E2.9_p1	E2.10_p1	E2.11_p1	E2.12_p1	E2.13_p1	E2.14_p1	E2.15_p1	E3.1_p1	E3.2_p1	E3.3_p1
Embryo_Name	Day3-E1	Day3-E1	Day3-E1	Day3-E1	Day3-E1	Day3-E1	Day3-E1	Day3-E2	Day3-E2	Day3-E2
ADAMTS1	1,70652	2,17380	2,52517	0,20664	1,85997	1,78217	1,19530	1,05067	0,00000	0,00000
C3	0,56618	0,00000	0,00000	0,00000	0,00000	1,20672	0,00000	0,00000	0,00000	0,00000
CNR2	0,00000	0,46716	0,00000	0,00000	0,00000	0,00000	0,00000	0,00000	0,00000	0,00000
CRABP1	0,00000	0,00000	0,00000	0,00000	0,00000	0,00000	0,00000	0,66911	0,00000	0,00000
DDX3Y	5,90762	4,69118	0,85387	0,91629	3,16993	1,44073	1,14362	3,71031	7,79258	1,25233
DPPA4	4,23326	5,77717	5,41114	0,00000	2,44475	5,05658	7,75389	5,48468	6,78392	1,63374
FOLR1	0,00000	0,00000	1,91246	0,00000	0,00000	0,00000	0,00000	0,00000	0,00000	0,00000
GAT	2,55554	2,62287	0,77764	0,00000	0,33919	0,00000	0,00000	0,32453	2,25735	0,00000
GFRA3	0,00000	0,00000	0,00000	0,00000	0,00000	0,00000	0,00000	1,20672	1,54772	0,00000

Cell_Name	E3.5_p1	E3.6_p1	E3.7_p1	E3.8_p1	E3.9_p1	E3.10_p1	E3.11_p1	E3.12_p1	E3.13_p1	E3.14_p1
Embryo_Name	Day3-E2	Day3-E2	Day3-E2	Day3-E2	Day3-E2	Day3-E2	Day3-E2	Day3-E2	Day3-E2	Day3-E2
ADAMTS1	1,66432	0,73619	0,00000	0,00000	0,00000	2,39962	0,60125	0,00000	2,97292	2,84016
C3	0,00000	0,00000	0,00000	0,00000	1,80685	0,00000	0,00000	0,29132	0,00000	0,00000
CNR2	0,21505	1,29530	0,00000	0,00000	0,00000	0,00000	0,00000	0,00000	0,48920	0,00000
CRABP1	0,00000	3,19320	0,00000	0,00000	3,46774	0,00000	0,00000	0,00000	0,76644	0,00000
DDX3Y	6,13153	5,81517	5,30807	5,37088	4,55931	3,31293	4,93356	4,20484	5,71509	4,04883
DPPA4	6,18611	7,48980	4,03056	2,27131	5,35014	8,58115	5,64034	1,56187	8,39554	7,79700
FOLR1	0,00000	1,15505	0,00000	0,00000	0,00000	0,00000	0,00000	0,00000	0,00000	0,00000
GAT	1,64999	0,00000	0,00000	0,00000	0,00000	0,74219	0,00000	0,00000	0,00000	0,00000
GFRA3	1,14578	0,28137	1,14311	2,17148	0,00000	0,00000	2,58438	0,00000	0,00000	0,53358

Cell_Name	E3.16_p1	E10.1_p1	E10.2_p1	E10.3_p1	E10.4_p1	E10.5_p1	E10.6_p1	E10.7_p1	E10.8_p1	E10.9_p1
Embryo_Name	Day3-E2	Day3-E3	Day3-E3	Day3-E3	Day3-E3	Day3-E3	Day3-E3	Day3-E3	Day3-E3	Day3-E3
ADAMTS1	0,00000	2,10601	0,00000	0,00000	2,78388	0,00000	0,00000	1,27617	0,00000	0,00000
C3	0,00000	0,00000	0,00000	0,00000	0,00000	0,00000	0,00000	0,00000	2,16539	0,00000
CNR2	0,00000	0,00000	0,00000	0,00000	0,00000	0,00000	0,00000	0,77318	1,87314	0,00000
CRABP1	0,00000	0,00000	0,00000	0,00000	0,00000	0,00000	0,00000	1,80193	0,00000	0,00000
DDX3Y	3,56555	5,05763	4,41229	4,92009	3,41588	2,19744	8,10857	6,40778	7,73907	4,39095
DPPA4	5,54957	2,83468	1,51136	4,63438	8,63919	2,97179	6,01336	5,45593	4,78498	6,80227
FOLR1	0,00000	2,75428	1,03674	0,00000	0,00000	0,00000	0,00000	0,00000	0,00000	0,00000
GAT	0,82119	0,76627	0,00000	0,00000	1,22866	0,85846	0,00000	0,00000	0,00000	1,17720
GFRA3	0,00000	0,00000	0,00000	0,00000	3,32379	0,00000	0,00000	0,00000	0,00000	0,00000

Supplementary Table S7 (continued). Gene normalised log2(UMI+1) values.

Cell_Name	E10.10_p1	E10.11_p1	E10.12_p1	E10.13_p1	E10.14_p1	E10.15_p1	E10.16_p1	E16.1_p1	E16.2_p1	E16.3_p1
Embryo_Name	Day3-E3	Day3-E3	Day3-E3	Day3-E3	Day3-E3	Day3-E3	Day3-E3	Day3-E4	Day3-E4	Day3-E4
ADAMTS1	1,39298	1,20278	1,77948	0,00000	0,50636	2,21397	0,00000	0,00000	0,00000	0,00000
C3	0,00000	0,00000	0,00000	0,00000	0,00000	0,00000	0,00000	0,00000	0,00000	0,00000
CNR2	1,65544	0,69917	0,00000	0,00000	0,00000	0,00000	0,47210	0,00000	0,97550	1,62813
CRABP1	4,33652	0,85127	0,68272	0,00000	1,15558	1,70652	0,00000	1,13034	0,00000	0,90818
DDX3Y	7,12210	7,48980	4,13115	1,24307	6,27648	9,13454	3,48323	2,80305	5,38188	9,06169
DPPA4	9,11988	2,84058	6,46009	2,75026	6,26823	8,72613	1,77073	1,77427	0,00000	1,78888
FOLR1	0,00000	0,00000	3,46774	0,00000	0,00000	0,00000	0,00000	0,00000	0,00000	0,00000
GAT	0,00000	0,00000	0,00000	0,00000	1,46194	1,49838	0,00000	0,00000	0,00000	0,00000
GFRA3	0,00000	0,00000	0,49486	0,00000	0,00000	0,00000	0,00000	0,00000	0,00000	0,00000

Cell_Name	E16.4_p1	E16.5_p1	E16.6_p1	E16.7_p1	E16.8_p1	E16.9_p1	E16.10_p1	E16.11_p1	E16.12_p1	E16.13_p1
Embryo_Name	Day3-E4	Day3-E4	Day3-E4	Day3-E4	Day3-E4	Day3-E4	Day3-E4	Day3-E4	Day3-E4	Day3-E4
ADAMTS1	0,00000	0,00000	0,00000	0,79088	0,00000	0,00000	0,00000	0,00000	0,00000	0,00000
C3	0,00000	0,00000	0,00000	0,00000	0,00000	0,00000	0,00000	0,00000	0,00000	0,00000
CNR2	1,50080	2,80277	6,12416	1,49776	4,18915	4,06814	4,29410	5,80731	1,12446	0,00000
CRABP1	3,42731	1,19530	0,44060	0,00000	0,33919	1,05992	0,73619	0,58930	2,73805	0,00000
DDX3Y	7,24418	3,04423	4,03767	4,86010	8,63919	4,42863	8,09567	5,39466	6,01138	3,91837
DPPA4	5,66681	3,66755	4,76343	1,34479	3,63189	4,52073	5,56796	3,08754	2,90538	0,33919
FOLR1	0,00000	0,00000	0,00000	0,00000	0,91107	0,00000	0,96697	0,00000	5,30037	0,00000
GAT	0,00000	0,00000	0,00000	1,16539	0,00000	0,00000	0,00000	0,00000	0,00000	0,00000
GFRA3	1,69725	0,00000	0,00000	0,00000	0,00000	0,24529	0,00000	2,60759	1,13506	0,00000

Cell_Name	E6.1_p3	E6.2_p3	E6.3_p3	E6.4_p3	E6.5_p3	E6.6_p3	E6.7_p3	E6.8_p3	E6.9_p3	E6.10_p3
Embryo_Name	Day3-E5	Day3-E5	Day3-E5	Day3-E5	Day3-E5	Day3-E5	Day3-E5	Day3-E5	Day3-E5	Day3-E5
ADAMTS1	1,32528	1,32149	0,00000	0,00000	1,21990	0,00000	0,00000	1,14311	1,46071	0,00000
C3	0,00000	0,00000	0,00000	0,00000	0,00000	0,00000	0,00000	0,00000	0,00000	0,00000
CNR2	0,00000	0,00000	0,00000	0,45003	0,00000	0,00000	0,00000	0,00000	0,00000	0,00000
CRABP1	1,04905	0,00000	0,00000	2,97292	0,00000	0,00000	2,92790	1,24192	0,00000	0,00000
DDX3Y	7,26164	4,14799	5,59246	4,10689	5,04581	8,02373	3,17647	3,41719	7,79650	2,62211
DPPA4	4,28110	4,47928	0,81293	3,38582	2,58127	5,00026	1,84203	5,87633	4,93963	6,68168
FOLR1	0,00000	0,00000	0,31678	1,09211	0,00000	0,00000	0,00000	0,00000	0,00000	1,19530
GAT	2,54859	0,71900	0,00000	0,00000	0,95719	0,56313	2,58438	0,00000	0,00000	0,00000
GFRA3	0,00000	0,00000	0,00000	0,00000	0,25927	0,00000	0,00000	0,00000	0,00000	0,00000

Cell_Name	E6.11_p3	E6.12_p3	E6.13_p3	E6.14_p3	E6.15_p3	E6.16_p3	E6.17_p3	E13.1_p3	E13.2_p3	E13.3_p3
Embryo_Name	Day3-E5	Day3-E5	Day3-E5	Day3-E5	Day3-E5	Day3-E5	Day3-E5	Day3-E6	Day3-E6	Day3-E6
ADAMTS1	0,00000	0,00000	0,00000	0,00000	0,29132	2,61685	1,21841	0,00000	0,00000	4,24988
C3	0,00000	0,00000	0,00000	0,00000	0,00000	0,00000	0,00000	0,00000	0,00000	0,00000
CNR2	0,00000	0,00000	0,00000	0,93555	0,00000	0,00000	0,00000	0,91107	0,00000	1,54075
CRABP1	0,00000	0,00000	0,00000	0,00000	0,00000	0,00000	0,00000	1,14829	0,55796	1,42322
DDX3Y	1,70652	5,43112	4,04075	1,05517	3,00241	5,32305	3,65643	4,36765	2,88016	4,03323
DPPA4	1,22985	4,59306	0,00000	5,52133	3,29727	2,91041	2,72397	3,14091	1,64048	3,80602
FOLR1	0,00000	0,00000	0,00000	0,00000	0,00000	0,32453	0,00000	0,77576	0,00000	0,00000
GAT	0,00000	1,21841	0,00000	0,27318	3,32379	0,85679	0,00000	0,62097	0,00000	0,00000
GFRA3	0,00000	0,00000	0,00000	0,00000	0,00000	0,00000	0,00000	0,00000	0,00000	0,00000

Supplementary Table S7 (continued). Gene normalised log2(UMI+1) values.

Cell_Name	E13.4_p3	E13.5_p3	E13.6_p3	E13.7_p3	E13.8_p3	E13.9_p3	E13.10_p3	E13.11_p3	E13.12_p3	E13.13_p3
Embryo_Name	Day3-E6	Day3-E6	Day3-E6	Day3-E6	Day3-E6	Day3-E6	Day3-E6	Day3-E6	Day3-E6	Day3-E6
ADAMTS1	0,00000	1,83153	1,05368	0,00000	1,38160	0,00000	2,53187	1,15545	0,92325	1,29422
C3	0,00000	0,00000	0,00000	0,00000	0,47210	1,76252	0,00000	1,62813	0,00000	1,13458
CNR2	3,70179	0,00000	1,38304	0,00000	3,89069	0,31678	2,73537	0,00000	0,00000	1,05517
CRABP1	3,04945	1,75239	5,00083	0,62007	3,91057	2,21739	3,30978	0,62442	2,42780	3,41799
DDX3Y	4,75680	6,10662	3,65580	5,89742	5,49665	6,43518	5,06922	3,94819	7,40937	9,23290
DPPA4	4,41464	6,18191	4,79510	2,51649	4,12084	6,96281	4,03559	8,62168	4,09665	7,32810
FOLR1	0,00000	0,00000	0,00000	0,00000	0,00000	0,00000	0,00000	1,30554	0,00000	0,00000
GAT	2,99490	1,54265	0,00000	0,92325	1,29422	0,00000	1,77968	2,81711	0,00000	2,85510
GFRA3	0,00000	0,00000	0,00000	0,00000	0,00000	0,00000	0,00000	0,00000	0,00000	1,03250

Cell_Name	E13.14_p3	E6.1_p2	E6.2_p2	E6.3_p2	E6.4_p2	E6.6_p2	E6.7_p2	E6.8_p2	E6.9_p2	E6.10_p2
Embryo_Name	Day3-E6	Day3-E7	Day3-E7	Day3-E7	Day3-E7	Day3-E7	Day3-E7	Day3-E7	Day3-E7	Day3-E7
ADAMTS1	1,48054	0,00000	0,00000	0,00000	1,80497	0,00000	0,00000	0,89044	0,00000	1,41544
C3	0,00000	0,00000	0,00000	0,00000	2,65979	0,00000	0,00000	0,00000	0,00000	0,00000
CNR2	0,00000	3,42731	0,00000	0,00000	0,17612	0,33919	0,00000	0,73619	1,59363	2,25735
CRABP1	0,96561	1,58924	0,00000	0,00000	0,00000	0,00000	0,00000	0,00000	0,00000	0,73522
DDX3Y	4,40473	6,69286	2,69165	4,97964	5,97301	4,03009	4,09759	2,18742	4,72315	3,24925
DPPA4	4,77958	6,49444	1,05878	1,67372	5,55521	2,93927	1,58924	0,97174	4,20173	3,99245
FOLR1	0,00000	0,00000	0,00000	0,00000	0,00000	0,00000	0,00000	0,00000	0,00000	0,00000
GAT	0,00000	0,00000	0,00000	0,00000	0,00000	0,45486	1,28098	1,18505	0,67639	0,00000
GFRA3	0,00000	0,00000	0,00000	0,00000	2,75428	0,00000	0,00000	0,00000	0,00000	0,00000

Cell_Name	E6.11_p2	E6.12_p2	E6.13_p2	E6.14_p2	E6.15_p2	E6.16_p2	E7.1_p2	E7.2_p2	E7.3_p2	E7.5_p2
Embryo_Name	Day3-E7	Day3-E7	Day3-E7	Day3-E7	Day3-E7	Day3-E7	Day3-E8	Day3-E8	Day3-E8	Day3-E8
ADAMTS1	0,24529	1,28098	1,18505	1,13506	0,81481	0,00000	0,00000	1,28485	0,00000	0,00000
C3	2,34289	0,00000	0,00000	0,00000	0,00000	0,00000	0,00000	0,00000	0,00000	0,00000
CNR2	0,77576	0,00000	0,00000	0,00000	2,97292	2,02875	1,59366	3,82985	1,89928	0,00000
CRABP1	0,00000	0,28137	0,00000	1,46071	0,00000	0,56313	3,08684	1,48233	1,55009	0,00000
DDX3Y	4,61941	5,58092	4,07165	3,38458	4,68969	7,39589	4,55502	8,95796	5,16599	3,52716
DPPA4	3,43133	3,24727	3,67504	5,30273	4,38815	4,85255	1,75224	4,16349	3,90876	1,27040
FOLR1	0,00000	0,00000	0,00000	0,00000	0,00000	0,00000	0,00000	0,00000	0,00000	0,00000
GAT	0,11153	0,00000	0,00000	0,00000	0,00000	0,00000	0,00000	0,00000	0,00000	0,00000
GFRA3	0,00000	0,00000	0,00000	0,16610	3,48211	4,40102	1,39891	0,24595	0,94515	0,00000

Cell_Name	E7.6_p2	E7.7_p2	E7.8_p2	E7.9_p2	E7.10_p2	E7.11_p2	E7.12_p2	E7.13_p2	E7.14_p2	E7.15_p2
Embryo_Name	Day3-E8	Day3-E8	Day3-E8	Day3-E8	Day3-E8	Day3-E8	Day3-E8	Day3-E8	Day3-E8	Day3-E8
ADAMTS1	1,90650	0,00000	1,29913	0,48920	0,00000	0,00000	0,00000	0,00000	0,00000	2,67432
C3	0,00000	0,00000	0,00000	0,00000	0,00000	0,00000	0,00000	0,00000	0,00000	3,40622
CNR2	1,97948	0,46825	1,26407	1,94258	0,00000	0,00000	1,39298	3,51281	1,14829	1,53045
CRABP1	1,83473	1,21841	0,00000	0,00000	0,00000	0,85679	0,00000	1,38611	0,20218	2,07421
DDX3Y	5,19347	8,46429	4,61818	6,50470	5,85599	5,06170	6,52990	4,75234	6,43901	5,03658
DPPA4	2,52975	6,42557	4,49365	5,23414	2,45291	4,24559	5,56808	5,74629	3,40458	8,26415
FOLR1	0,00000	1,16539	0,00000	0,00000	0,00000	0,00000	0,00000	0,00000	0,00000	0,28137
GAT	0,00000	0,00000	0,00000	0,00000	0,00000	0,00000	0,00000	0,77318	1,22135	0,00000
GFRA3	0,00000	0,00000	0,00000	2,47590	0,72074	1,74559	3,57161	1,46513	0,00000	1,54075

Supplementary Table S7 (continued). Gene normalised log2(UMI+1) values.

Cell_Name	E7.16_p2									
Embryo_Name	Day3-E8									
ADAMTS1	0,00000									
C3	0,00000									
CNR2	1,17720									
CRABP1	0,00000									
DDX3Y	5,64210									
DPPA4	4,35503									
FOLR1	0,00000									
GAT	0,00000									
GFRA3	3,17122									
